# Pyrazolone-based ERO1 inhibitors in ERO1-driven triple-negative breast cancer and SEPN1-related myopathy: Structure–activity relationship and therapeutic potential

**DOI:** 10.1016/j.phrs.2025.108037

**Published:** 2025-11-19

**Authors:** Michele Retini, Alessandro Cherubini, Alice Marrazza, Ersilia Varone, Serena Germani, Giorgia Maria Renna, Adriano Recchia, Andrea Guidarelli, Stefano Fumagalli, Chiara Grasselli, Michele Mari, Giovanni Piersanti, Giovanni Bottegoni, Roberto Tonelli, Marco Gobbi, Bo-Ram Jin, Jaehyung Cho, Orazio Cantoni, Ester Zito

**Affiliations:** a Istituto di Ricerche Farmacologiche Mario Negri IRCCS, Milan, Italy; b Department of Biomolecular Sciences, University of Urbino Carlo Bo, Urbino, Italy; c Department of Molecular and Developmental Medicine, University of Siena, Siena, Italy; d Institute of Clinical Sciences, University of Birmingham, Edgbaston, Birmingham B15 2TT, UK; e Respiratory Intermediate Care Unit, Department of Surgical and Medical Sciences, University Hospital of Modena, Laboratory of Experimental Pneumology, University of Modena and Reggio Emilia, Italy; f Division of Hematology, Department of Medicine and Department of Pathology and Immunology, Washington University School of Medicine, St. Louis, USA

**Keywords:** ERO1A, Stress of the endoplasmic reticulum (ER stress), UPR (Unfolded protein response), Breast cancer, SEPN1-related Myopathy

## Abstract

Endoplasmic reticulum oxidoreductin 1 alpha (ERO1A) is a disulfide oxidase that facilitates oxidative protein folding by reoxidizing protein disulfide isomerase (PDI), a process essential for maintaining endoplasmic reticulum (ER) homeostasis. Under ER stress, ERO1A expression is upregulated via the unfolded protein response (UPR), promoting cell survival. However, sustained ERO1A activity can impair proteostasis and contribute to disease. Notably, ERO1A is overexpressed in triple-negative breast cancer (TNBC), where it supports tumor growth and adaptation to hypoxia, and in SEPN1-related myopathy, a rare congenital muscle disorder linked to ER and oxidative stress. To investigate ERO1A as a therapeutic target, we conducted a structure–activity relationship (SAR) study of EN460-based pyrazolone inhibitors. Forty derivatives and three EN460 salts were synthesized to optimize potency and solubility. In vitro and cell-based assays revealed that effective inhibition required covalent binding to Cys397, interactions with Arg287 and Trp200, and distortion of the phenyl ring. While sulfonic acid substitution improved solubility, it abolished activity by disrupting key interactions. The most potent compound, I29, featuring a mono ortho-fluorine substitution, demonstrated improved inhibitory activity (IC_50_ = 2.6 μM) and efficacy in preclinical models of TNBC and SEPN1-related myopathy. These findings highlight ERO1A’s pathological role in cancer and congenital muscle disease and support its inhibition as a promising therapeutic strategy for conditions characterized by chronic ER and oxidative stress.

## 1. Introduction

Endoplasmic reticulum oxidoreductin 1 alpha (ERO1A, hereafter referred to as ERO1) is a flavin-dependent disulfide oxidase localized in the endoplasmic reticulum (ER), where it plays a central role in oxidative protein folding [1,2]. By reoxidizing protein disulfide isomerase (PDI), ERO1 facilitates the formation of disulfide bonds in newly synthesized proteins. This redox reaction, in which molecular oxygen serves as the final electron acceptor, is coupled with the production of hydrogen peroxide (H_2_O_2_)—a reactive oxygen species that, if not adequately disposed, can contribute to oxidative stress and cellular damage.

ERO1 is also involved in the oxidation of key cysteine residues on calcium regulators such as sarco-endoplasmic reticulum calcium ATPase (SERCA) pumps, the ryanodine receptor (Ryr), and inositol 1,4,5-triphosphate receptor (IP3r) thereby modulating calcium fluxes, mitochondrial function, cellular bioenergetics, muscle contraction and eventually stress-induced apoptosis [3–6].

Under conditions of endoplasmic reticulum (ER) stress, ERO1 expression is upregulated as part of the unfolded protein response (UPR)—a tightly regulated, multilayered adaptive pathway aimed at restoring proteostasis by attenuating protein synthesis, enhancing protein folding capacity, and promoting degradation of misfolded proteins [7,8]. While transient ERO1 activation supports ER recovery by promoting oxidative protein folding, persistent or excessive ERO1 activity has been implicated in various pathological conditions [9–11]. In cancer, elevated ERO1 levels enhance tumor cell survival under hypoxic or nutrient-deprived conditions, promote angiogenesis, glycolysis and contribute to increased invasiveness and resistance to therapy [12–21]. Accordingly, ERO1 genetic deletion has been shown to slow tumor growth by impairing Vascular endothelial growth factor A (VEGFA) signaling, altering its N-glycosylation [14,22] and activating anti-tumor immunity [23,24]. In certain congenital myopathies ERO1 levels are upregulated [25,26], and in Selenoprotein N1 (SEPN1)-related myopathy, one of these congenital myopathies, ERO1 levels have been associated with maladaptive UPR signaling, worsening disease progression and muscle dysfunction. Consistently, genetic inhibition of ERO1 in preclinical models of SEPN1-RM improved muscle phenotype [27].

These findings have spurred interest in developing small-molecule ERO1 inhibitors as potential therapeutic agents for diseases marked by ERO1 overexpression or dysregulation. EN460, a covalent inhibitor of ERO1, has served as a valuable tool compound for probing ERO1 function [28]. However, its limited aqueous solubility and moderate potency present challenges for further pharmacological development and clinical translation.

In this study, we performed a comprehensive structure–activity relationship (SAR) analysis of EN460-based ERO1 inhibitors. By chemically modifying key pharmacophoric groups of the EN460 scaffold, we synthesized a library of 43 novel derivatives with the goal of improving both inhibitory potency and aqueous solubility. Using a kinetic in vitro assay that employed recombinant ERO1 and its partner PDIA1 to recapitulate the electron transfer between the two proteins, we assessed ERO1 activity and identified structural features essential for its inhibition. Furthermore, we evaluated the compounds’ uptake and efficacy both in cells and *in vivo*. The most promising molecule, I29—featuring an IC_50_ twofold lower than EN460—was further tested for efficacy in models of breast cancer and SEPN1-related myopathy. Our findings offer important insights into the chemical and biological features required for effective ERO1 inhibition *in vivo* and support its therapeutic relevance in disease contexts.

## 2. Materials and methods

### 2.1. EN460 and analogues I23–I40

EN460 and I1–I23 analogues were previously synthesized [29]. EN460 derivatives I24–I40 and EN460 salts were synthesized for this essay in the laboratory of Pharmaceutical Chemistry of Uniurb. The experimental procedures for synthesis of I24–I40, the NMR spectra, HPLC analysis, high-resolution mass spectrometry (ESI-TOF), yield, and purity of compounds are attached in the Supplementary Methods. All EN460 derivatives were dissolved in DMSO (at 25 μM) and diluted at the final concentrations indicated for *in vitro* and *in cell* assays. EN460 salts (Lysine, Arginine and Dietanoleamine) were dissolved in aqueous solution (at 2,5 μM) and diluted at the final concentrations indicated *in cell* assays.

### 2.2. ERO1A and PDIAI recombinant proteins

A GST-SMT3-mouse ERO1A (residues 43–464) and His-tagged PDIA1 [30] were expressed in the Rosetta DE3 bacteria (Novagen) and purified as described in [29,31]. The GST-SMT3 and His tags were cleaved using Ulp protease (Z03691, GenScript) and thrombin protease (T6884, Merck), respectively. Successful tag removal was confirmed by a shift to a lower molecular weight for ERO1A on SDS-PAGE and by the absence of signal in anti-His immunoblotting for PDIA1.

### 2.3. Reagents

Bovine insulin, EDTA, and dithiothreitol were purchased from Sigma Aldrich (St. Louis, MO, USA). Isoquercetin, a flavonoid anti-oxidant PDI inhibitor, was purchased from Cayman Chemical (Ann Arbor, MI, USA). Human monoamine oxidase A (Mao-A), clorgyline (a Mao-A inhibitor), and Mao-A activity assay kit were obtained from Sigma-Aldrich (St. Louis, MO, USA).

### 2.4. PDI activity assay

PDI activity was assessed using an insulin transhydrogenase assay. Bovine insulin (1 mg/mL) in sodium phosphate buffer, pH 7.0, containing 2 mM EDTA, was incubated with 1.4 μM recombinant PDI in the presence of vehicle (1 % DMSO) or various concentrations of isoquercetin (30 or 60 μM), EN460, I2, I26, or I29 (1, 5, or 25 μM) for 15 min. The turbidimetric assay of insulin disulfide reduction by PDI was initiated by adding 1 mM dithiothreitol, and the reaction was monitored at 650 nm for 80 min. PDI activity is presented as a percentage of the vehicle control.

### 2.5. Mao-A activity assay

Mao-A activity was measured using a commercial assay kit according to the manufacturer’s instructions. Mao-A (0.837 μM) was incubated with vehicle (1 % DMSO), Clorgyline (50 μM), or various concentrations (1, 5, or 25 μM) of EN460, I2, I26, or I29 for 10 min. Mao-A catalyzes the oxidative deamination of p-tyramine, generating H_2_O_2_, which was quantified fluorimetrically (λ_ex_= 530; λ_em_= 585 nm). Mao-A activity is presented as a percentage of the vehicle control.

### 2.6. Chemoinformatics analysis to compare known Mao-A and Mao-B inhibitors to the structure of EN460

Molecules annotated for activity against monoamine oxidase A (Mao-A, UniProt ID: P21397) and monoamine oxidase B (Mao-B, UniProt ID: P27338) were retrieved from the ChEMBL database [32]. Compounds with pChEMBL values ≥ 6 (corresponding to bioactivity ≤ 1 μM) and assay confidence scores ≥ 8 were selected to ensure the inclusion of high-quality and well-annotated activity data. The resulting dataset was exported as a comma-separated values (CSV) file containing canonical SMILES representations for all compounds. All similarity calculations were conducted in Python (v3.11) using the pandas and RDKit libraries. Molecular fingerprints were generated as Extended-Connectivity Fingerprints (ECFP4) using the GetMorganFingerprintAsBitVect function (radius = 2, nBits = 2048), corresponding to circular fingerprints with a diameter of four bonds.

The reference compound EN460 was represented by the SMILES string:

O═C(C1 =C(Cl)C=CC(N2N═C(/C(C2 =O)=C\C3 =CC=C(O3) C4 =CC=CC=C4)C(F)(F)F)=C1)O. For each compound in the MAO dataset, an ECFP4 fingerprint was generated from its canonical SMILES, and the Tanimoto similarity between each molecule and EN460 was computed using RDKit’s DataStructs.TanimotoSimilarity function.

### 2.7. AUR-based kinetic assay of ERO1A activity

The kinetic assay of ERO1A activity is based on a reaction involving recombinant ERO1A and PDIA1 proteins, horseradish peroxidase (Worthington), and 5 μM Amplex Ultra Red, as detailed in [29]. The effect of ERO1A inhibitors was evaluated by preincubating the compounds with ERO1A and measuring the inhibition of the linear reaction rate, and the corresponding IC_50_ values were determined using Prism 10 (GraphPad).

### 2.8. Tumor cell lines

Cells were kept in culture for no more than two weeks and routinely tested for mycoplasma infection. MDAMB231, ERO1A KO MDAMB231 human breast cancer cells, E0771 and ERO1A KO E0771 murine breast cancer cells were described in [29].

### 2.9. Effect of inhibitors on ERO1A redox status in vivo

MDAMB231 and E0771 cells were maintained in Dulbecco’s Modified Eagle’s Medium supplemented with 1 % fetal bovine serum and seeded at 70 % confluency in six-well plates one day prior to the experiment. Cells were treated with DTT (10 mM) and/or ERO1A inhibitors for 1–5 h. At the time of collection, phosphate-buffered saline containing 10 mM NEM was added, and cells were incubated on ice for 10 min in lysis buffer (50 mM Tris-HCl, pH 7.4; 150 mM NaCl; 1 % Triton X-100; 0.1 % SDS; 1 % sodium deoxycholate; protease inhibitors; and 10 mM NEM). Protein concentrations were determined using the BCA assay, and equal amounts of protein were separated by non-reducing SDS-PAGE followed by immunoblotting with a polyclonal antibody against ERO1A [33].

### 2.10. MTS

Six thousand cells (MDAMB231 and E0771) per well were seeded in 96-well plates and cultured in medium containing 1 % FBS. Cells were treated with varying concentrations of compounds and, after 24 h, incubated with MTS [3-(4,5-dimethylthiazol-2-yl)-5-(3-carboxymethoxyphenyl)-2-(4 sulfophenyl)2Htetrazolium]and PMS (phenazine methosulfate), following the instructions of the CellTiter 96^®^ Aqueous Non-Radioactive Cell Proliferation Assay (Promega). Measurements were performed using a TECAN Infinite M200 plate reader with excitation at 490 nm.

### 2.11. Animals

Female C57BL/6 J mice, aged 8–10 weeks, were obtained from Charles River Laboratories (Calco, Italy) and housed under specific-pathogen-free conditions. These mice were injected with E0771 cells. All procedures were conducted in compliance with Italian legislative decree D.lgs. 26/2014 (authorization 19/2008-A issued on 6 March 2008 by the Ministry of Health; authorization 896/2023-PR to E. Zito), the Mario Negri Institutional Regulations and Policies (including internal authorization for personnel conducting animal experiments, Quality Management System Certificate—UNI EN ISO 9001:2008, registration number 6121), EU directives (EEC Council Directive 2010/63/EU), and the *Guidelines for the Welfare and Use of Animals in Cancer Research* [15].

Wild-type (WT) and SEPN1 knockout (KO) C57BL/6 male mice, aged 4 months, were fed either a selenium-deficient diet or a diet containing 0,4 ppm selenium for four months (cod. 10105101, Charles River). Experimental protocols followed D.lgs. 26/2014 (authorization 19/2008-A; authorization 465/2023-PR to E. Zito). All animal experiments were performed in accordance with the ARRIVE guidelines and complied with relevant national and international laws, regulations, and policies governing the care and use of laboratory animals, including Italian law.

### 2.12. Formulation of I29 for studies in mice

A 1:1 mixture of polyethylene glycol 400 (PEG400) (Merck, 8.17003.1000) and Cremophor EL (Sigma, C5135–500G) was used to dissolve I29 at a concentration of 10 mM and served as the vehicle control. Initially, I29 was suspended in PEG400, after which Cremophor EL was added to the solution. The mixture was then heated to 80 °C until the compound was fully dissolved. Both the compound and vehicle were diluted 1:10 in physiological saline immediately prior to administration to mice at a dose of 10 mg/kg.

### 2.13. Breast tumor model and treatment

E0771 cells (1 × 10^6^) were injected subcutaneously into both flanks of 12 female C57BL/6 J mice. Once the primary tumors reached a volume of 100–200 mm^3^ (palpable), the mice were randomized into two groups and treated with either I29 or vehicle (Cremophor and PEG400). I29 was administered intraperitoneally (i.p.) once daily for 10 consecutive days at a dose of 10 mg/kg. Twenty-four hours after the final injection, the mice were euthanized. Tumors were excised, weighed, and processed for flow cytometry analysis of immune cells and immune checkpoints, including PD-1 and PD-L1, as well as for VEGFA quantification by ELISA.

### 2.14. Flow cytometry analysis of breast tumors

The procedure was described in detail in [29]. Briefly, primary subcutaneous tumors derived from E0771 cells were minced into small fragments and enzymatically digested with 0.5 mg/mL Collagenase IV (Sigma) and 40 μg/mL DNase I in RPMI 1640 medium for 30 min at 37°C. The resulting cells were resuspended in HBSS containing 0.5 % FBS and incubated at 4°C for 20 min with the following antibodies (BioLegend): CD45 BV650 (clone 104; 0.6/100), CD11b PerCP-Cy5.5 (clone M1/70; 0.3/100), Ly6C BV421 (clone HK1.4; 0.6/100), Ly6G AF700 (clone 1A8; 0.6/100), F4/80 PE-Cy7 (clone BM8; 0.3/100), PD-L1 APC (clone 10 F.9G2; 1/100), CD3 BV605 (clone 145–2C11; 0.6/100), CD4 AF700 (clone GK1.5; 0.6/100), CD8 BV785 (clone 53–6.7; 0.6/100), and PD1 (clone 29.F1A12; 0.6/100). Cell viability was determined using the LIVE/DEAD^™^ Fixable Near IR Viability Kit (Invitrogen, L34981). Samples were acquired using a Cytoflex LX flow cytometer with CytExpert software, and data were analyzed with Kaluza 2.0 software (Beckman Coulter).

Within the CD45 + leukocyte population, monocytic myeloid-derived suppressor cells (M) MDSCs (CD11b+Ly6G−Ly6Chi), polymorphonuclear granulocytic (PMN−) MDSCs (CD11b+Ly6G+Ly6Clo), and tumor-associated macrophages (TAMs; CD11b+Ly6Clo/−F4/80 +) were identified. Total T lymphocytes (CD3 +) were gated within CD45 + population, and frequencies of CD3 +CD4 + and CD3 +CD8 +T cells subsets were quantified. PD1 expression was evaluated on CD4 + and CD8 + T cells, while PD-L1 expression was assessed on MDSCs and TAMs. Expression levels are reported as geometric mean fluorescence intensity (gMFI).

### 2.15. VEGFA ELISA

For VEGFA measurements in E0771 tumors, tumors were weighed, and 50 mg of each was isolated and lysed in PBS containing protease inhibitors but no EDTA. Following homogenization, samples were frozen overnight and then subjected to repeated freeze-thaw cycles the next day. Insoluble debris was removed by high-speed centrifugation, and the supernatant was quantified using the BCA assay. A total of 190 μg of protein was then analyzed using a mouse VEGF Quantikine ELISA Kit (MMV00, R&D Systems).

### 2.16. Treatment with I29 in WT and SEPN1 KO Mice

Wild-type (WT) and SEPN1 knockout (KO) male mice, previously fed a selenium-deficient diet or a diet containing 0.4 ppm selenium, were randomized to receive intraperitoneal (i.p.) injections of I29 or vehicle alone. Treatments were administered once daily for five consecutive days at a dose of 10 mg/kg. Twenty-four hours after the final injection, mice underwent whole-body plethysmography (Whole Body Plethysmography by emkaTechnologies) under baseline conditions in ambient air to assess several respiratory parameters reflecting ventilatory efficiency. In particular, tidal volume (TV), breathing frequency (f), peak inspiratory flow (PIF), and mean inspiratory flow (TV/inspiratory time [TI]) were measured. Both intra- and inter-subject coefficients of variation (CV) for TV and f were also computed.

### 2.17. WT and SEPN1 KO Myotubes

Muscle stem cells were isolated from muscles of 1-month-old wild-type (WT) and SEPN1 knockout (KO) mice using a tissue dissociation protocol with the gentleMACS^™^ Octo Dissociator with Heaters (Miltenyi Biotec, Bergisch Gladbach, Germany), followed by magnetic depletion. To generate myotubes, cells were cultured on Matrigel-coated Ibidi μ-Slide 8 well chambers (Cat. No. 80826, Ibidi GmbH, Gräfelfing, Germany) in DMEM (EuroClone, Pero, Milan, Italy) supplemented with 20 % fetal bovine serum (EuroClone), 3 % chick embryo extract (Cat. 11485015, Fisher Scientific SAS), 10 ng/mL basic fibroblast growth factor (Cat. 13256029, Life Technologies Europe BV), 1 % GlutaMAX (Gibco; Thermo Fisher Scientific, Waltham, MA, USA), and 1 % penicillin-streptomycin (EuroClone).

After 48 h of proliferation, cells were cultured for an additional 2 days in medium containing 2 % horse serum (EuroClone) to induce differentiation, then harvested for analysis. For ERO1 redox state, cells were treated for 3 h with either EN460 or I29, harvested and followed by a non-reducing ERO1 Immunoblot.

### 2.18. TMRM staining

After 16 h of treatment with EN460 or I29 in differentiation medium, the culture medium was removed and replaced with the TMRM staining solution for the assessment of mitochondrial membrane potential. Thus, the staining solution was prepared with 10 nM final of TMRM (MitoProbe^™^ TMRM Assay, Cat. No. M20036, Thermo Fisher Scientific, Waltham, MA, USA) in cell growth medium. TMRM fluorescence was acquired at λ_exc_ 561 nm / λ_em_ 595 nm by a Nikon A1 confocal system equipped with an Okolab stage incubator (CO_2_ 5 %, 37°C, 90 % humidity) immediately after TMRM and over a 10-minute baseline period with 65 s time interval. Then, oligomycin (Cat. LIFJ61898.MA, Life Technology Europe BV) was added at 1 mg/mL in culture media and acquired for 25 min at 65 s time interval. Carbonyl cyanide trifluoromethoxy Phenylhydrazone (FCCP) (Cat. MRKC2920, MERK life Science SRL) was then added at 4 mM and the signal acquired over 20 min with 65 s time interval. We used a 20x NA 0.50 objective lens to obtain 0.61 μm/pixel microphotographs sized 625×625×7 μm, step size 2.3 μm. Gray value range was kept below oversaturation. Data were analyzed using ImageJ. Briefly, background noise was set to NaN and the remaining signal quantified as mean pixel gray values. Data were expressed as ΔF/F_0_ (F_0_= mean of baseline signal).

### 2.19. Real-time quantitative RT-PCR analysis

RNA from WT and SEPN1KD C2C12 was reverse-transcribed and analyzed using the Applied Biosystems’ real-time PCR System and the ΔΔCt method. Relative gene expression in cells was normalized to Actin mRNA levels as in [27].

### 2.20. Super resolution microscopy

Structured illumination microscopy (SIM) was done on a Nikon SIM system with a 100 × 1.49 objective. Briefly, randomly selected cells were imaged at laser excitation of 405 nm (nuclei), 561 nm (ER), and 640 nm (mitochondria) with a 3D-SIM acquisition protocol. SIM images were quantified with ImageJ. After background normalization, the ER signals was segmentated to calculate the volume of the ER expressed as fraction of the cell. The segmentated mitochondrial signal was followed by the skeletonize function of ImageJ to perform mitochondria network analysis. We then applied the Analyze Skeleton 2D/3D plugin of ImageJ to measure each cell total branch length, expressed as μm.

### 2.21. Statistics

Data were analyzed using Prism 10 (GraphPad Software). The statistical tests used, as well as sample sizes (*N*), are indicated in the figure legends, except for dot plots. Comparisons between two normally distributed groups were performed using an unpaired two-tailed *t*-test. For comparisons among multiple groups, one-way ANOVA followed by Tukey’s post hoc test was used. For grouped analyses, two-way ANOVA with Holm–Sidak correction for multiple comparisons was applied. Results are presented as mean ± SEM or mean ± SD, as specified in the figures. A *p*-value < 0.05 was considered statistically significant.

## 3. Results

### 3.1. Chemical substitution of EN460-based ERO1 inhibitors

**Fig. 1.**
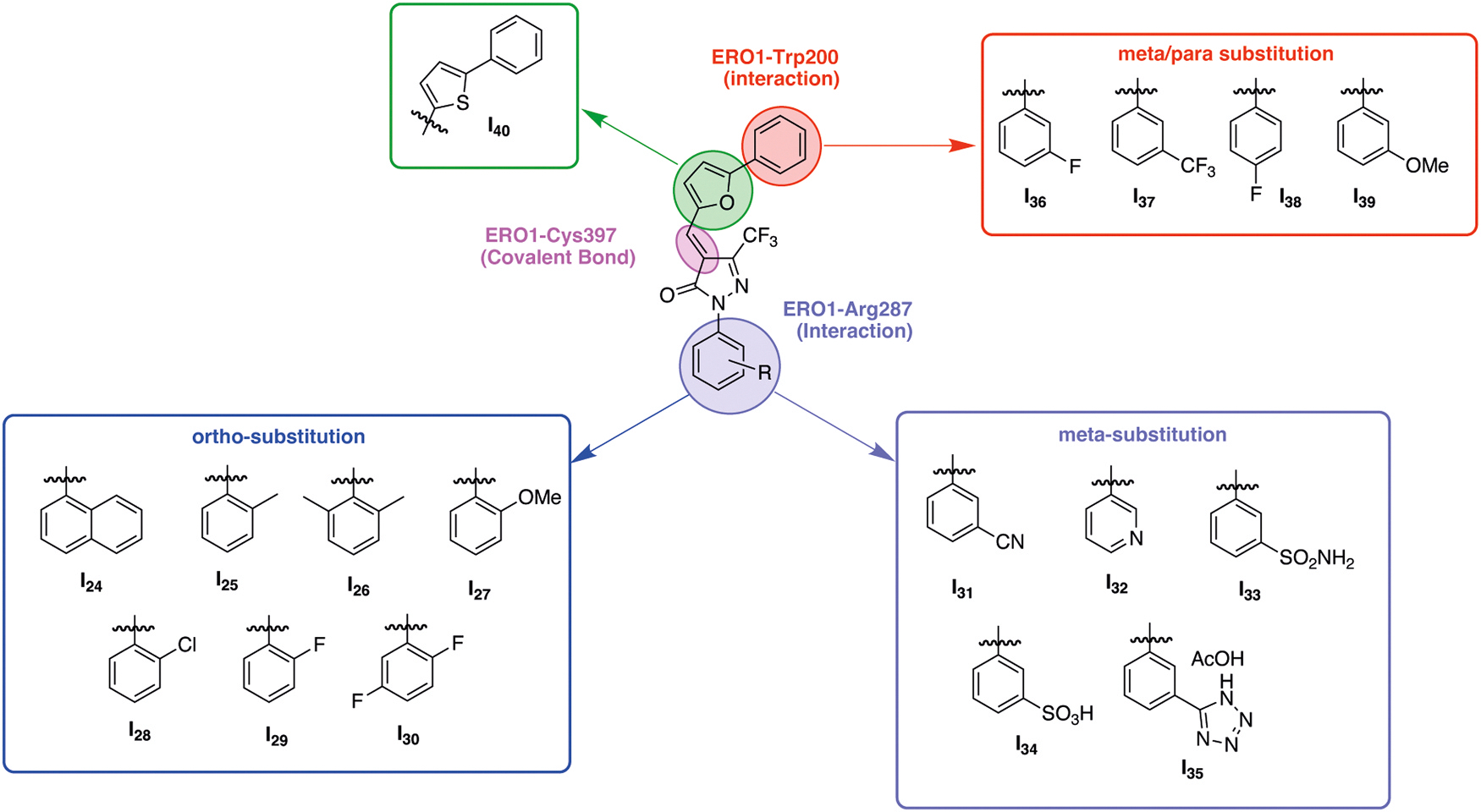
Chemical structure of EN460 derivatives, Chemical structure of EN460. The different pharmacophoric groups are highlighted with colored boxes, and the amino acids of ERO1 that interact with these groups are color-coded to match: violet indicates the phenyl ring that interacts with Arg287 of ERO1, red highlights the phenyl furan moiety that interacts with Trp200, and purple marks the Michael acceptor that covalently binds to Cys397. Analogs I24–I30 contain various ortho substitutions on the phenyl ring (blue box), while I31–I35 feature substituents at the meta position of the same ring (violet box). Analogs I36–I39 exhibit modifications on the phenyl furan moiety (red box), and analog I40 includes a thiophene ring replacing the furan ring (green box).

**Fig. 2.**
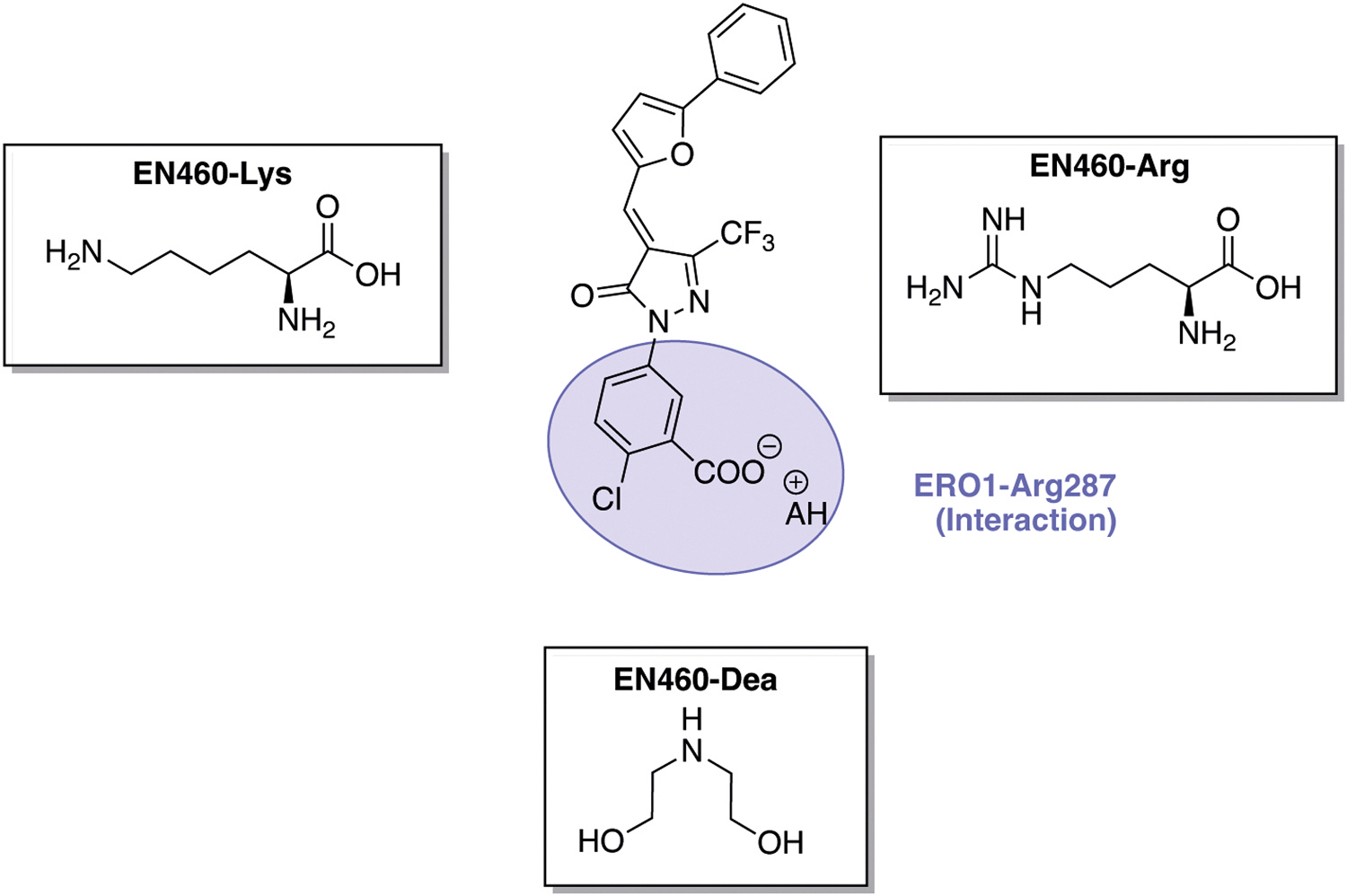
Chemical structure of EN460 salts, Chemical structure of EN460. The counterions associated with the carboxylate anion on the phenyl ring of EN460 are highlighted in purple and may include lysine (Lys), arginine (Arg), or diethanolamine (Dea). The interaction with Arg287 of ERO1—also shown in purple—is depicted as well.

In addition to the initial set of 23 EN460 analogs that we previously designed and analyzed by modifying the Michael acceptor, phenyl and phenyl furan moieties [29], we synthesized 16 additional compounds to assess whether chemical modifications of the phenyl and phenyl furan moieties and three salt forms of EN460 (with arginine, lysine, and diethanolamine) could enhance both inhibitory potency and aqueous solubility (Figs. 1 and 2). Here, we conducted a structure–activity relationship (SAR) study on previously synthesized compounds (I1–I23) [29] to better understand how specific chemical substitutions affect ERO1 inhibitory activity.

From the SAR analysis of these 23 compounds, we determined that several structural features are critical for activity: (1) the presence of a Michael acceptor is essential for covalent interaction with Cys397 of ERO1; (2) the phenyl ring distortion (non-planar structure) is essential for ERO1 inhibition and can be achieved by the interaction between the carboxyl group of EN460 and Arg287 of ERO1 or by an ortho-substituent on the phenyl group; (3) the phenyl furan moiety plays an important role in the non-covalent interaction with Trp200 [29]. Based on these findings, we designed two different series of new compounds by introducing bioisosteric groups of carboxylic acid or by hydrogen bond donor/acceptor in meta position to enhance Arg287 interaction (I31–I35, shown in purple) or by ortho-substitution (I24–I30, shown in blue) that disrupt molecular planarity of the phenyl ring (Fig. 1). The second group of pharmacophoric modifications concerned introduction of different electron donating (EDG) or electron withdrawing group (EWG) in the phenyl furan moiety (I36–39 highlighted in red) and was aimed at maximizing the interaction with Trp200 to improve the potency (Fig. 1). Finally, I40 (in green) was designed to determine the effect of oxygen replacement in the furan ring with sulfur for ERO1 inhibition (Fig. 1). To improve aqueous solubility of EN460 we designed three salts of EN460: namely, a Lysine, Arginine and diethanolamine salt (Fig. 2).

### 3.2. Inhibition of ERO1 activity by EN460-derivatives

**Fig. 3.**
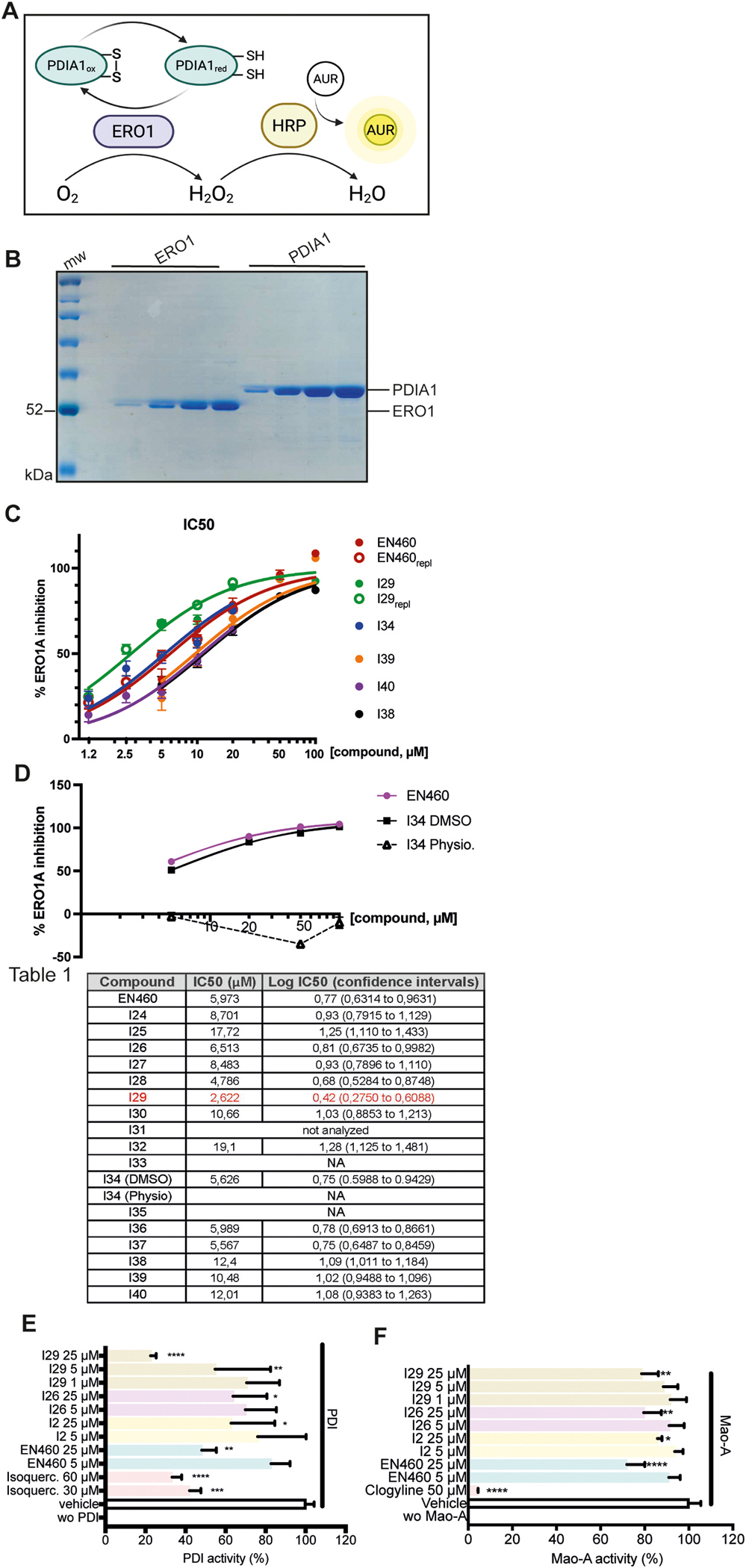
Effects of EN460 derivatives on *in vitro* ERO1 activity,A) Assay scheme: The activity of recombinant ERO1A protein is measured using a fluorescence-based assay that detects H_2_O_2_ production during the oxidation of recombinant reduced PDIA1. Horseradish peroxidase (HRP) utilizes the H_2_O_2_ generated by ERO1 to oxidize Amplex UltraRed (AUR) into a fluorescent product. B) Coomassie-stained SDS-PAGE showing different concentrations of ERO1 cleaved from the upstream GST_SUMO tag using ULP protease (loaded amounts: 110, 460, 920, and 1380 ng), and reduced PDIA1 cleaved from the upstream His tag using thrombin (loaded amounts: 250, 1000, 2000, and 3000 ng). C) Inhibition plots of the indicated compounds: The percentage of ERO1 inhibition is plotted against the logarithm of compound concentration (replic. stands for replicates). D) Comparison of I34 dissolved in either DMSO or physiological solution (aqueous solution). Table 1 shows the IC_50_ values of the compounds, their logarithmic IC_50_ values, and corresponding confidence intervals (NA stands for not active, I31 was not analyzed for toxicity issues due to CN). E) Bar graph showing PDI activity measured using the insulin turbidity assay (N = 3; mean ± SD; one-way ANOVA). PDI activity was normalized to 100 % for the vehicle-treated sample, and all other samples were compared to the vehicle-treated group (Isoquerc. = Isoquercetin). F) Bar graph showing MAO-A activity (N = 3; mean ± SD; one-way ANOVA). MAO-A activity was normalized to 100 % for the vehicle-treated sample, and all other samples were compared to the vehicle-treated group.

EN460 and 16 newly synthesized derivatives (I24–I40) (except for I31, which was not evaluated due to potential toxicity associated with its CN group) were evaluated for their ability to inhibit ERO1-dependent AUR fluorescence using a kinetic assay with recombinant ERO1 and PDIA1, as previously described [29] (Figs. 3A and 3B). Raw kinetic data showing AUR fluorescence over time in reactions containing reduced ERO1, PDIA1 and EN460-derivatives are presented in Fig. Sup.1 A and Sup. 1B. Reaction rates, measured in presence or absence of varying concentrations of each compound, were used to calculate IC_50_ values, defined as the concentration required to inhibit 50 % of ERO1 enzymatic activity (Figs. 3C, 3D and Fig. Sup1C).

The IC_50_ values of the ortho-substituted phenyl ring derivatives (I24–I30) were generally similar, with the exception of the methyl substituted compound (I25), which exhibited the lowest potency, and the fluorine-substituted compound (I29), which exhibited the highest potency, displaying an IC_50_ more than two times lower than EN460, at around 2.6 μM. In contrast, meta substitutions on the phenyl ring (I31–I35) largely abolished inhibitory activity, except for I34, which carries a sulfonic acid group (Table 1in Fig. 3 and Fig. Sup 1B). Notably, I34 retained activity when dissolved in DMSO, with an IC_50_ comparable to EN460, but was completely inactive in aqueous solution (physiological solution) (Fig. 3D, Table 1in Fig. 3 and Fig. Sup1B).

Regarding modifications of the phenyl furan moiety, electron-withdrawing groups at the meta position of the phenyl ring (I36 and I37) did not significantly affect inhibitory activity compared to EN460. However, para-fluorine substitution (I38), a meta methoxy group (I39), or replacement of the furan ring with thiophene (I40) all resulted in a loss of inhibitory activity (Table 1in Fig. 3). Altogether, these findings indicate that ortho fluorine substitution on the phenyl ring (as in I29) yields the most potent inhibitor among the derivatives tested. Meta substitutions were generally dispensable, except for acidic moieties such as in I34, which were necessary for activity. The loss of I34 activity in aqueous solution is likely due to sulfonic acid deprotonation and salt formation, which disrupts its interaction with Arg287. Overall, modifications on the phenyl furan moiety were either neutral (I36 and I37) or detrimental (I38, I39, and I40) to inhibitory activity (Table 1 in Fig. 3).

Additionally, the three salt forms of EN460 (lysine, arginine, and diethanolamine) were tested in a cell-based assay. Inhibition of ERO1 in cells leads to the accumulation of a reduced isoform that is unable to exchange electrons with PDI [28,29]. Therefore, we assessed the redox state of ERO1 in MDA-MB-231 breast cancer cells treated with EN460 or its three salts at 20 μM using non-reducing immunoblotting (Fig. Sup. 2 A). EN460 efficiently reduced ERO1 and this effect was comparable to that of its arginine salt. In contrast, the lysine and diethanolamine salts showed weaker ERO1 reduction. A dose-response experiment (5–20μM) further compared EN460 and its arginine salt in MDA-MB-231. At all concentrations tested, the arginine salt gave a weaker ERO1 reduction. Furthermore, while EN460 achieved complete ERO1 reduction at 15 μM, the arginine salt only partially reduced ERO1 at the highest tested concentration (20 μM) indicating lower potency relative to the parent compound (Fig. Sup. 2B).

To assess whether EN460 and its derivatives act as PDI inhibitors, EN460 and its derivatives I2, I26, and I29 were evaluated at concentrations of 1, 5, and 25 μM using the classical insulin turbidity assay [34], with quercetin included as a known reference inhibitor. All compounds inhibited PDI activity at 25 μM (Fig. 3E).

To evaluate the potential of EN460 to inhibit Mao-A and Mao-B, we performed a chemoinformatics analysis comparing the structure of EN460 with those of known MAO-A and MAO-B inhibitors. Data were retrieved from ChEMBL [32], a publicly available database of molecules annotated for activity against literature-reported targets. Compounds strongly active against Mao-A (383 molecules), Mao-B (1812 molecules), or both (338 molecules) were compared to EN460. Among Mao-A-active compounds, the most structurally similar molecules retained the general scaffold of EN460; for example, the benzylidene-5-phenylisoxazole-3-carbohydrazide structure in CHEMBL5404145 overlaps with the phenylfuran-pyrazol-3-one core of EN460. Variations in phenyl-ring substituents, which were examined in this study, appear to play only a minor role. In contrast, the *meta*--carboxy substitution pattern seems more relevant for structural similarity to Mao-B-active compounds. However, Mao-B inhibitors are generally smaller than EN460, which may partly explain the lower affinity of EN460 for this isoform. Based on this chemoinformatics analysis comparing known Mao-A and Mao-B inhibitors to the structure of EN460, it is likely that our lead compound, I29, retains the cross-reactivity toward both Mao isoforms exhibited by its parent compound.

EN460, I2, I26, and I29 were also tested at 1, 5, and 25 μM for Mao-A inhibitory activity using clorgyline as a known Mao-A inhibitor. Compared with the vehicle control, EN460, I2, I26, and I29 at 25 μM inhibited Mao-A activity (Fig. 3F).

### 3.3. Cell-based assays to identify EN460 derivatives active as ERO1 inhibitors

**Fig. 4.**
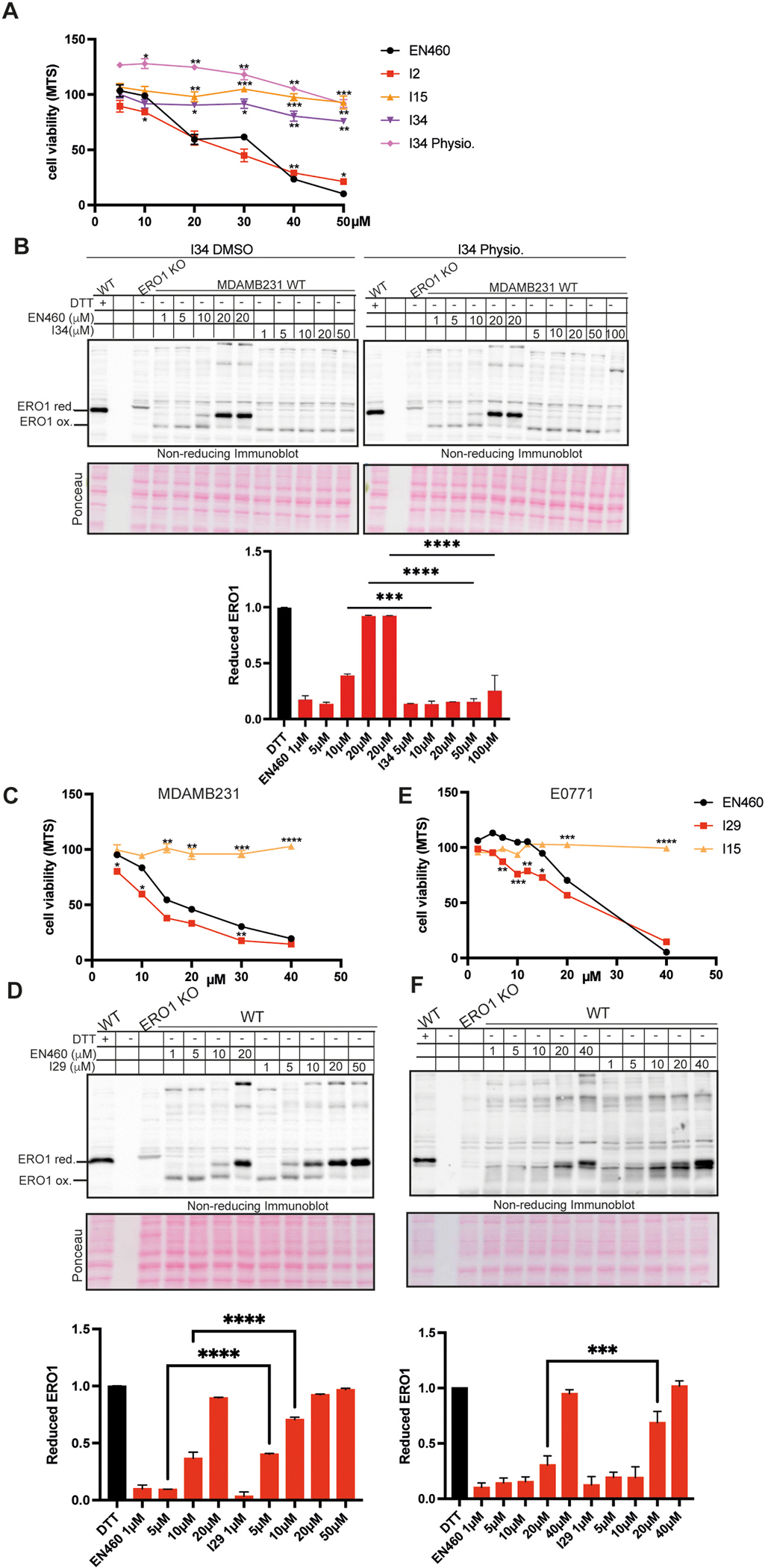
EN460 derivatives act as ERO1 inhibitors *in vivo*, A) MTS assay of MDAMB231 cells treated with EN460 or the indicated inhibitors (I). The viability of cells treated with vehicle alone was arbitrarily set to 100 %, and values for treated cells were calculated as percentages (N = 3, mean ± SD, Two-way ANOVA vs. EN460). B) Non-reducing immunoblot of endogenous ERO1 in lysates from WT and ERO1 KO MDAMB231 cells exposed to DTT, EN460, or I34 dissolved in DMSO (left) or physiological solution (right). “ERO1 red.” indicates reduced ERO1, “ERO1 ox.” indicates oxidized ERO1. Ponceau staining shows equal protein loading. Below, the bar graph represents the ratio of reduced to total ERO1 (N = 2, mean ± SD, One-Way ANOVA). C) MTS assay of MDAMB231 cells treated with EN460, I29, or I15 (N = 3, mean ± SD, Two-way ANOVA vs. EN460). D) Non-reducing immunoblot of endogenous ERO1 in lysates from WT and ERO1 KO MDAMB231 cells treated with DTT or the indicated concentrations of EN460 or I29. Ponceau staining shows equal protein loading. Below, the related bar graph represents the ratio of reduced to total ERO1 (oxidized + reduced), arbitrarily set to 1 for DTT-treated cells (N = 2, mean ± SD, One-Way ANOVA). E) MTS assay of E0771 cells treated with EN460, I29, or I15 (N = 3, mean ± SD, Two-way ANOVA vs. EN460). F) Non-reducing immunoblot of endogenous ERO1 in lysates from WT and ERO1 KO E0771 cells treated with DTT or the indicated concentrations of EN460 or I29. Ponceau staining shows equal protein loading. Below, the bar graph represents the ratio of reduced to total ERO1 (N = 2, mean ± SD, One-Way ANOVA).

To evaluate whether the *in vitro* ERO1 inhibition by new EN460 analogs translated into reduced cellular metabolic activity, we performed MTS assays in the TNBC MDA-MB-231 cells using a dose–response approach. We first tested compound I34 prepared in both physiological solution and DMSO-based solutions, and compared its effects to EN460, I2 (a previously synthesized active inhibitor [29]), and I15 (a previously synthesized compound lacking the Michael acceptor, and therefore inactive [29]) (Fig. 4A). The assay confirmed that both I2 and EN460 significantly reduced metabolic activity, while I15 and I34—whether dissolved in DMSO or physiological solution—were inactive.

To further investigate the lack of I34 activity (in DMSO or physiological solution), as we did with EN460 salts, we performed non-reducing immunoblotting to assess the redox state of ERO1 in MDA-MB-231 breast cancer cells following treatment. Cells were treated with increasing concentrations (1–20 μM) of EN460 or (1–100 μM) I34 (either in physiological solution or dissolved in DMSO), and ERO1 reduction was evaluated in a dose–response experiment (Fig. 4B). Unlike EN460, which induced near-complete reduction of ERO1 at 20 μM, I34 failed to reduce ERO1A under both conditions. These findings are consistent with the lack of an I34-dependent metabolic effect observed in the MTS assay, suggesting that although I34 (dissolved in DMSO) is active *in vitro*, it is completely inactive in cells—most likely due to poor cellular uptake.

Consistently with an in vitro assay indicating that I29 showed an IC50 two-fold lower than EN460, I29 significantly reduced metabolic activity (i.e., MTS assay) of MDAMB231 cells in a dose-dependent manner and showed higher potency than EN460. As expected, I15, which didn’t any effect in inhibiting ERO1, had no effect on the MTS assay either (Fig. 4C). Consistently, I29 reduced more efficiently ERO1 that its benchmark EN460 in MDAMB231 (Fig. 4D). The superior efficacy of I29 over EN460 was further confirmed in another breast cancer cell line, E0771, using both MTS measurements (Fig. 4E) and ERO1 redox assays (Fig. 4F). Together, these results identified I29, an EN460 analog, as the most potent ERO1 inhibitor *in vivo*.

### 3.4. Effect of I29 in breast cancer–bearing mice

**Fig. 5.**
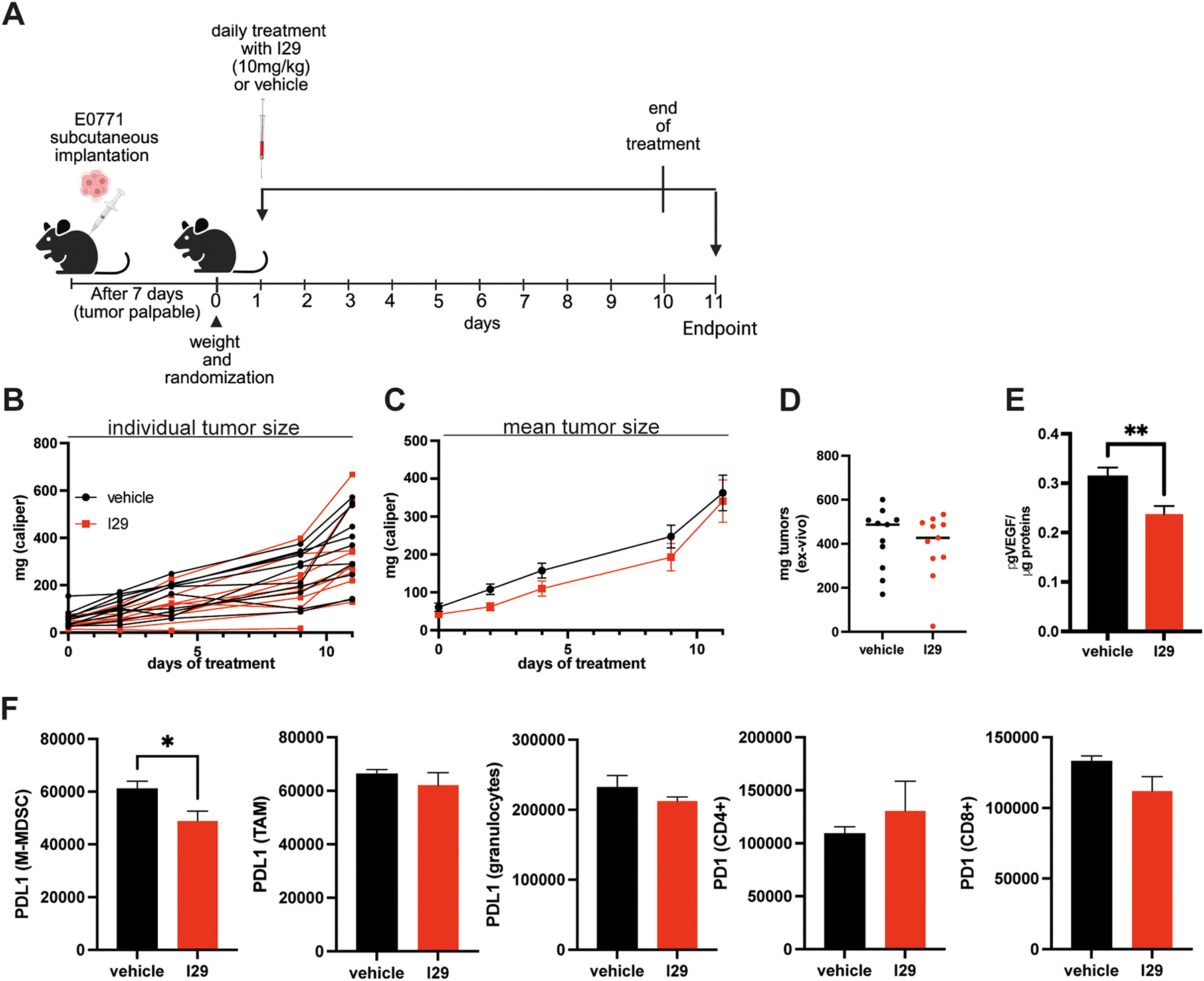
Anticancer effects of I29-mediated ERO1 inhibition in E0771 breast cancer–bearing syngeneic miceA) Schematic representation of E0771 subcutaneous implantation in C57BL/6 J mice and treatment schedule with the ERO1 inhibitor I29 or vehicle. B) Tumor growth curve based on caliper measurements of individual tumors from mice treated with I29 or vehicle (Two-Way ANOVA, N = 12 tumors per group). C) Tumor growth curve based on caliper measurements of the mean tumor size from mice treated with I29 or vehicle (Two-Way ANOVA, N = 12 tumors per group; data presented as mean ± SEM). D) Ex vivo tumor weights. E) Quantification of VEGFA levels in primary breast tumors by ELISA. F) Bar graphs showing quantification of PD-L1 and PD-1 expression (unpaired *t*-test; data presented as mean ± SEM).

Given the involvement of ERO1 in breast cancer aggressiveness and in the reduction of tumor immune surveillance [23,29], we investigated the *in vivo* effects of the novel ERO1 inhibitor, I29, in E0771 tumor–bearing syngeneic C57BL/6 J mice. Mice were implanted subcutaneously with E0771 cells, and treatment with intraperitoneally injected I29 began once tumors became palpable (i.e., after 7 days), continuing for ten days (Fig. 5A). The treatment was well tolerated, with no overt signs of toxicity or weight loss observed. Tumor volume analysis revealed no significant differences between I29-treated and vehicle-treated groups, although a slight reduction in tumor weight was observed in the I29-treated mice (Figs. 5B, 5C and 5D). However, since our previous studies demonstrated that ERO1 genetic deletion or inhibition by compound I2 reduced VEGFA levels [13–15,29], we assessed VEGFA expression in tumors from I29-treated mice using ELISA. Consistent with earlier findings, VEGFA levels were reduced in tumors from the I29-treated group (Fig. 5E). Given the evidence linking ERO1 activity to modulation of the tumor microenvironment [23,29], we further examined the impact of I29 treatment on tumor-infiltrating immune cell populations by flow cytometry. We focused on both myeloid and lymphoid subsets, particularly those involved in the PD-1/PD-L1 immune checkpoint axis [35]. I29 treatment resulted in a statistically significant reduction of PD-L1 expression in monocytic myeloid-derived suppressor cells (M-MDSCs), with a similar trend observed in tumor-associated macrophages (TAMs) and granulocytes. PD-1 expression on CD4^+^ and CD8^+^ T cells, however, remained unchanged (Fig. 5F). These findings support previous observations that intraperitoneal administration of ERO1 inhibitors is non-toxic [29]. While ERO1 inhibition had only a modest effect on tumor volume, it significantly reduced VEGFA expression and PD-L1 levels, suggesting that ERO1 inhibition may attenuate tumor aggressiveness by blunting both angiogenesis and immunosuppression within the tumor microenvironment.

### 3.5. Effect of I29 on WT and SEPN1-deficient muscle cells

We previously demonstrated that ERO1 is upregulated in preclinical models of the rare congenital myopathy SEPN1-related myopathy (SEPN1-RM) [27].

**Fig. 6.**
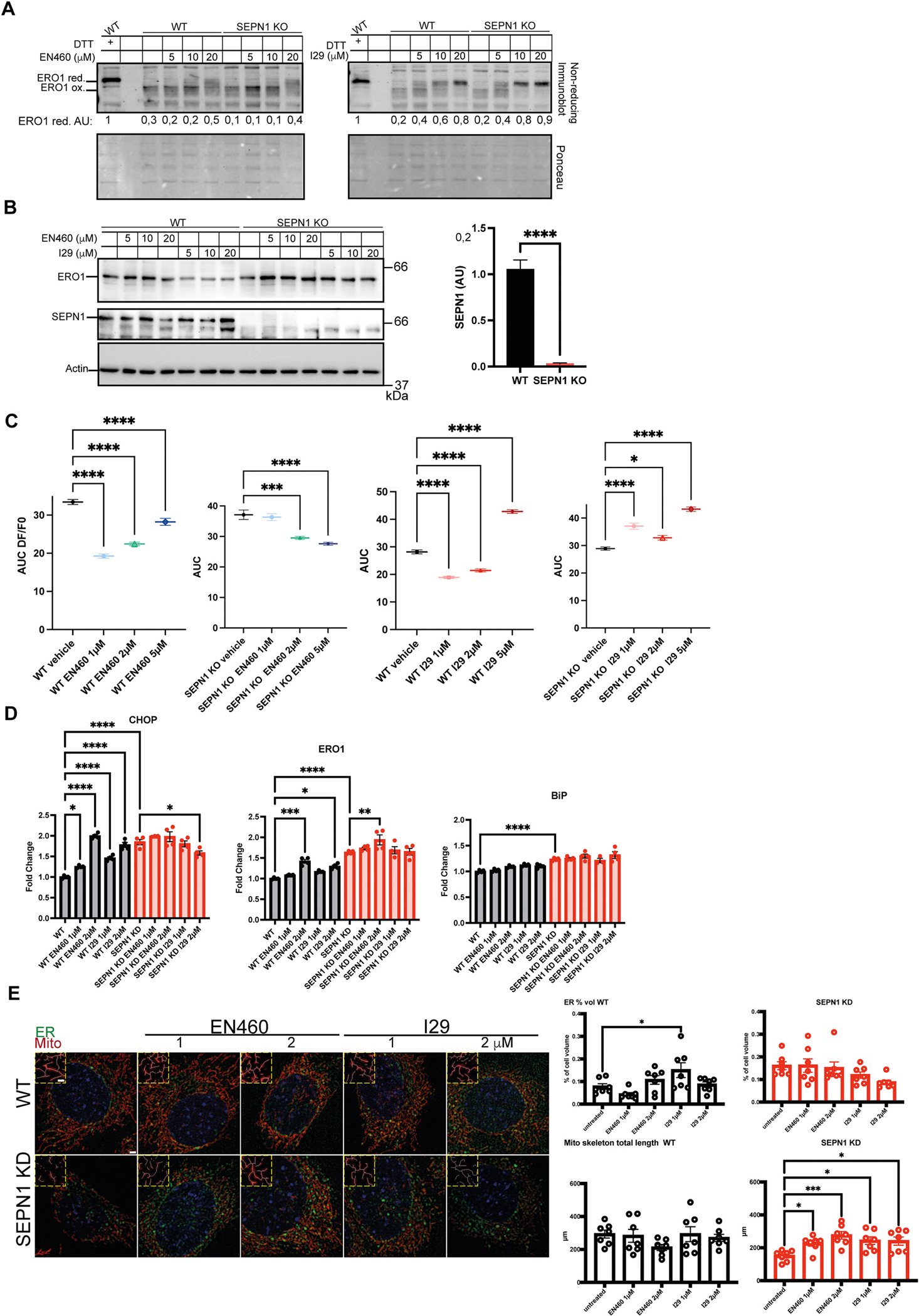
**Effect of I29 on WT and SEPN1depleted muscular cells,** A) Non-reducing immunoblots of endogenous ERO1 in lysates from WT and SEPN1 KO myotubes treated with DTT or the indicated concentrations of EN460 or I29. Below, arbitrary units (AU) represent the ratio between reduced ERO1 and total ERO1 (reduced + oxidized). Ponceau staining confirms equal protein loading. B) Reducing immunoblots of endogenous ERO1 and SEPN1 in lysates from WT and SEPN1 KO myotubes treated with the indicated concentrations of EN460 or I29. Actin immunoblot confirms equal protein loading. The bar graph on the right shows SEPN1 levels in AU in WT and SEPN1 KO myotubes. C) Area under the curve (AUC) analysis of DF/F_0_ plots of TMRM fluorescence in WT and SEPN1 KO myotubes treated with EN460 or I29 (N = 6, one-way ANOVA; data presented as mean ± SD). D) Quantitative real-time PCR analysis of cDNA from WT and SEPN1 KD C2C12 cells (N = 4; one-way ANOVA; data are presented as mean ± SEM). E) Representative images of ER (green) and mitochondria (red) trackers in WT and SEPN1 KD C2C12 cells (scale bar, 2 μm). Insets show magnified views of skeletonized mitochondria (scale bar, 1 μm). On the right, dot plots display the ER volume (vol) and the total mitochondrial branch length (quantified using the skeletonized mitochondrial signal) (N = 7; one-way ANOVA; data are presented as mean ± SEM).

To investigate the cellular effects of I29 in cellular models of SEPN1-RM, we tested its effect in primary myotubes derived from WT and SEPN1 KO mice and compared it to the known inhibitor EN460. I29 proved to be more effective than EN460 in promoting the reduced state of ERO1, as demonstrated by non-reducing ERO1 immunoblot analysis (Fig. 6A). Reducing immunoblot analysis of ERO1 showed a uniform band across all treated samples, indicating consistent ERO1 expression. In contrast, the SEPN1 blot confirmed the absence of SEPN1 protein in the KO cells, as evidenced by the lack of an immunoreactive band (Fig. 6B). We further assessed mitochondrial membrane potential—an important indicator of mitochondrial function—using TMRM (tetramethylrhodamine methyl ester) fluorescence in WT and SEPN1 KO myotubes treated with EN460 or I29 (1–5 μM) [36,37]. Notably, treatment of SEPN1 KO myotubes with I29—but not with EN460—resulted in a significant increase in TMRM fluorescence at all tested concentrations, suggesting a restoration of mitochondrial membrane potential and a likely overall improvement in mitochondrial function (Fig. 6C). EN460 and I29 (1 and 2 μM) were also evaluated for their ability to regulate ER stress in WT and SEPN1 KD C2C12 muscle cells [27]. To this end, the expression levels of the ER stress/response markers BIP, CHOP, and ERO1 were analyzed by quantitative real-time PCR. As expected, we confirmed higher levels of all three markers in SEPN1 KD cells. Notably, only I29 at 2 μM was able to downregulate CHOP expression (Fig. 6D).

Furthermore, we assessed ER volume and mitochondrial distribution in WT and SEPN1 KD cells treated with EN460 or I29, using a combination of endoplasmic reticulum- and mitochondria-specific trackers together with super-resolution microscopy. As previously observed [38], SEPN1 KD cells displayed an impaired mitochondrial network, quantified by skeletonizing the mitotracker signal. Treatment with EN460 or I29 selectively improved the mitochondrial network in SEPN1 KD cells, whereas ER volume did not change substantially (Fig. 6E).

Collectively, these data support the conclusion that pharmacological inhibition of ERO1 by I29 has beneficial effects in SEPN1-RM muscular cells and may represent a viable therapeutic approach for this disease.

### 3.6. Effect of I29 on whole-body plethysmography in WT and SEPN1 Knockout (KO) mice

**Fig. 7.**
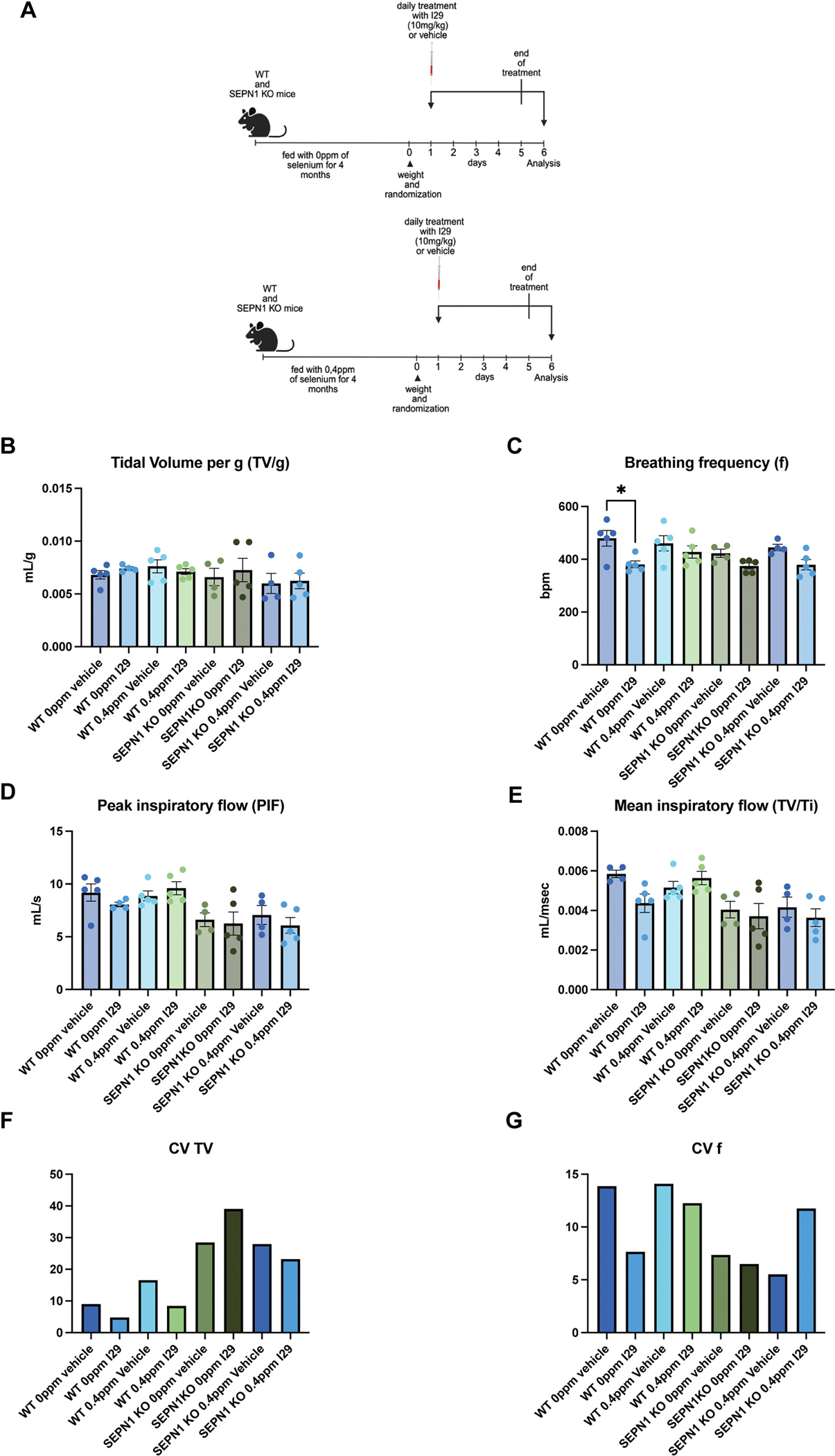
Effect of I29 on whole-body plethysmography in WT and SEPN1 KO mice, A) Schematic representation of the treatment protocol: WT and SEPN1 KO mice were treated with the ERO1A inhibitor I29 or vehicle while being fed a selenium-deficient diet (0 ppm) or a diet containing 0.4 ppm selenium. B) Bar graph showing tidal volume normalized to body weight (mL/g). C) Bar graph showing breathing frequency (breaths per minute). D) Bar graph showing peak inspiratory flow (mL/s). E) Bar graph showing mean inspiratory flow (mL/msec.) (N = 4–5 per group; one-way ANOVA). F) Bar graph showing the coefficient of variation (CV) for tidal volume in each group, indicating inter-subject variability. CV was calculated as the standard deviation divided by the group mean. G) Bar graph showing the CV of breathing frequency per group, indicating inter-subject variability. CV was calculated as the standard deviation divided by the group mean.

Genetic inhibition of ERO1 has been shown to improve calcium handling, enhance endoplasmic reticulum (ER)–mitochondria contact sites, and increase diaphragmatic muscle strength in SEPN1 KO mice [27]. Based on these findings, we tested the effect of the ERO1 inhibitor I29, administered via intraperitoneal injection, in SEPN1 knockout (KO) mice—a well-established preclinical model of SEPN1-related myopathy (SEPN1-RM). To further exacerbate the SEPN1 KO phenotype, the mice were maintained on a selenium-deficient diet (0 ppm selenium) for four months. This cohort was compared to a control group that received a diet containing 0.4 ppm selenium, slightly above the levels typically found in standard animal chow (Fig. 7A).

Whole-body plethysmography showed that selenium deficiency did not exacerbate respiratory dysfunction in either WT or SEPN1 KO mice (Fig. 7B–G). Vehicle-treated SEPN1 KO mice on both selenium diets exhibited increased variability in tidal volume (CV TV) and reduced variability in respiratory frequency (CV f), indicative of impaired ventilatory efficiency and irregular respiratory rhythm, compared to their WT counterparts (Fig. 7F and G). Notably, the selenium-deficient and selenium-supplemented (0.4 ppm) diets did not result in significant differences in the severity of respiratory impairment among SEPN1 KO mice (Fig. 7F and G).

In WT mice, I29 induced a mild improvement in respiratory parameters, independent of the selenium content in the diet (Fig. 7F and G). However, I29 treatment failed to improve respiratory function in SEPN1 KO mice and was associated with distinct ventilatory patterns depending on selenium status. In selenium-deficient KO mice, I29 increased CV TV, reflecting irregular and unstable breathing. In contrast, selenium-supplemented KO mice exhibited a consistently reduced CV TV following I29 administration, which, along with a concomitant reduction in CV f, suggests a more rigid and non-adaptive breathing pattern (Fig. 7F and G).

The mechanisms underlying the lack of efficacy of I29 in this disease model remain unclear and will be further investigated through a detailed pharmacokinetic analysis of I29 distribution in the diaphragm and accessory respiratory muscles of SEPN1 KO mice.

## 4. Discussion

ERO1 is a disulfide oxidase of the endoplasmic reticulum (ER) that facilitates oxidative protein folding by transferring electrons through its partner protein PDI. This process introduces disulfide bonds into newly synthesized client proteins, promoting proteostasis [39]. In this redox relay, molecular oxygen serves as the final electron acceptor and is rapidly reduced to hydrogen peroxide (H_2_O_2_), a reactive oxidant that, at high concentrations, can contribute to oxidative stress and maladaptive cellular response [2].

The H_2_O_2_ generated by ERO1 activity can be utilized by PRDX4, an ER-localized peroxidase that couples H_2_O_2_ reduction to PDI re-oxidation. This not only supports ERO1 activity but also prevents excessive accumulation of H_2_O_2_ [40–42] and also explain why, in mammals, ERO1 loss of function results in only modest defects [43]. However, ERO1 is upregulated in several disease contexts—including different cancer types [12,44], cardiac diseases [4] and congenital myopathies [25,27,45] — where it contributes to the pathogenesis. Its limited impact on healthy tissue, combined with its upregulation and mechanistic involvement in disease, makes ERO1 an ideal candidate pharmacological target for a so-called ‘magic bullet’ [46].

**Fig. 8.**
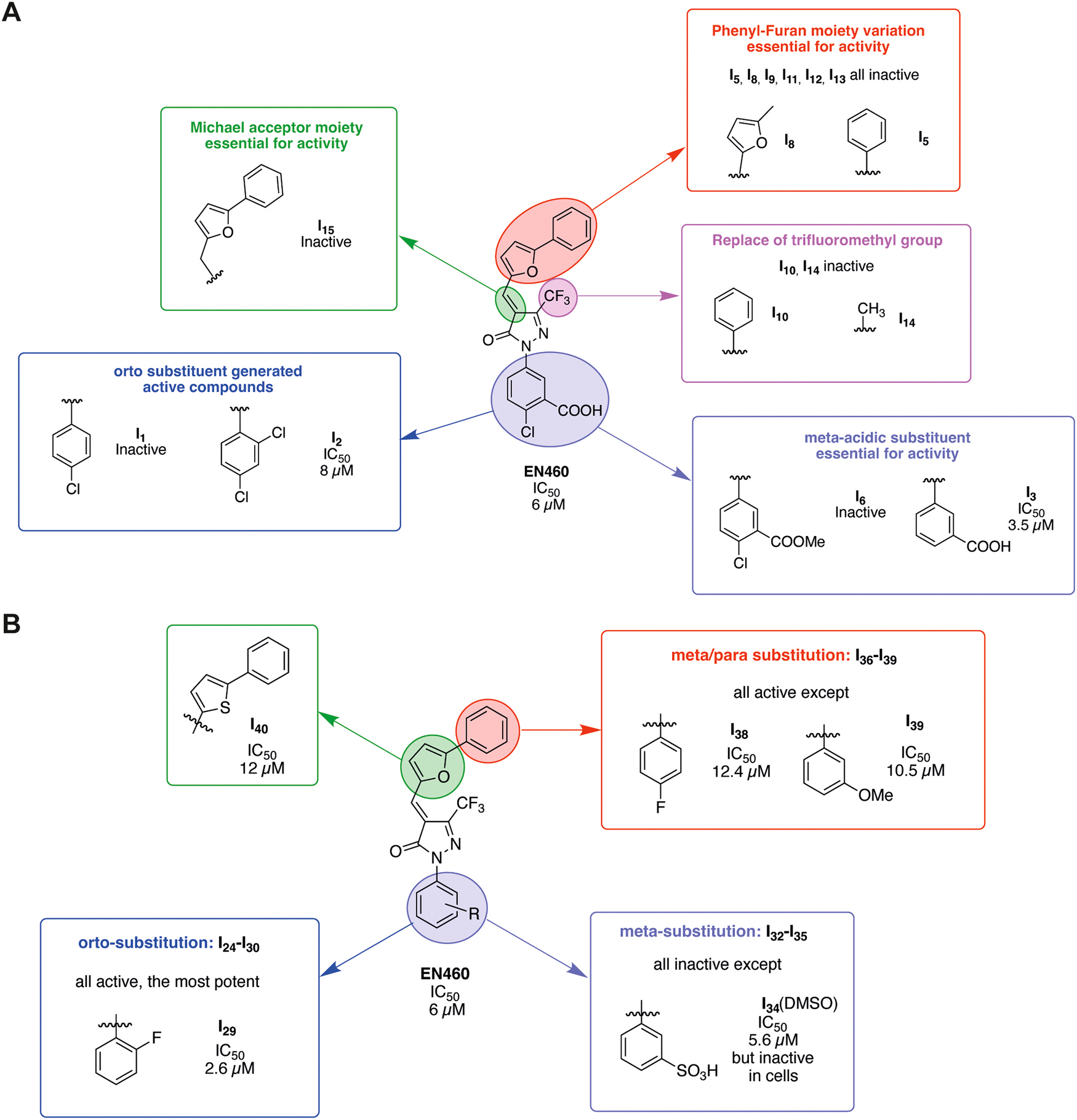
SAR of EN460 derivatives (I1–I40), A) Chemical structure of EN460, as shown in Fig. 1. The structure–activity relationship (SAR) analysis of compounds I1–I23 is presented. B) The structure–activity relationship (SAR) analysis of compounds I24–I40 is presented.

One of the first ERO1 inhibitors identified was EN460, a pyrazolone-containing compound [47], which covalently targets Cys397 of ERO1 via an electrophilic Michael acceptor moiety, displacing the FAD cofactor and suppressing ERO1 activity [28]. More recently, compound B12–5 was discovered as a novel ERO1 inhibitor; although structurally distinct, it similarly binds Cys397 of ERO1 thereby displacing the FAD cofactor and like EN460, suffers from poor aqueous solubility [30]. Early attempts to improve EN460’s potency using azide derivatives or natural compounds met with limited success [48,49].To address these limitations, we initiated a structure–activity relationship (SAR) campaign aimed at optimizing both potency and water solubility of EN460 derivatives. Our docking studies indicated EN460 binds to ERO1 through a covalent interaction between its electrophile Michael acceptor and Cys397 and through key non-covalent interactions: the phenyl furan moiety engages a π-stacking interaction with Trp200, the carboxylate group on the phenyl ring interacts with Arg287, and the trifluoromethyl group occupies a sub-pocket near the backbone of Glu186 (Fig. 1) [29]. Guided by this model, we chemically and systematically modified these groups to improve potency and water solubility. Overall, we synthesized 40 new EN460 derivatives (I1–I40) and three salt forms, systematically modifying key pharmacophoric groups: the Michael acceptor, the trifluoromethyl group, the phenyl ring, and the phenyl furan moiety. Our findings confirmed that the Michael acceptor is essential for inhibitory activity, and its removal (e.g., compound I15) resulted in complete loss of inhibition (Fig. 8A). SAR and docking analyses further highlighted the importance of non-covalent interactions between the inhibitor’s phenyl ring and ERO1 residue Arg287, and between the phenyl furan moiety and Trp200 [29].

EN460’s inhibitory activity can be achieved by the interaction between an acidic group in meta position and Arg287, or a mono ortho substituent of the phenyl ring that leads to its distortion (Fig. 8A). For instance, compound I1 (with a para-chlorine) was inactive, while I2 (with an ortho-chlorine) and I3 (with a meta-carboxylate) retained activity. Several other ortho-substituted derivatives (I24–I30) showed good potency (Fig. 8B), with I29—bearing a mono ortho-fluorine—emerging as most potent, exhibiting approximately twofold higher activity than EN460 (IC_50_ 2,6μM).

The meta-acidic moiety on the phenyl ring, as in I34, was essential for maintaining activity in DMSO, but the compound became inactive in water. This was likely due to sulfonic acid deprotonation and salt formation, which disrupted interaction with Arg287 (Fig. 8B). Similarly, salt forms of EN460 improved water solubility but lost activity, likely for the same reason.

Replacing the trifluoromethyl group with a methyl (I14) or phenyl (I10) abolished the activity (Fig. 8A). The phenyl furan moiety is critical for Trp200 interaction. While introducing electron-withdrawing groups in the meta position of the phenyl furan (I36, I37) maintained the potency, para substitution with fluorine (I38) and meta substitution with a methoxy group (I39) resulted in loss of activity, likely due to weakened interactions with Trp200. Furthermore, replacing the furan ring with a thiophene (I40) also reduced activity, likely due to impaired interaction with the backbone of Glu186 (Fig. 8B).

From these SAR studies, we drew several key conclusions: (1) The Michael acceptor is essential for ERO1 inhibition; (2) salt formation or acidic sulfonate substitution of phenyl ring, while improving solubility, abolishes biological activity; and thus, it is unlikely that this chemical class can be optimized to yield water-soluble compounds without sacrificing potency; (3) phenyl furan moiety is essential for the activity (5) the phenyl distortion is necessary for the activity and can be enhanced by a mono ortho-fluorine substitution. In conclusion, the mono ortho-fluorine substitution in I29 enhanced phenyl ring distortion and emerged as our lead compound, which displayed a twofold increase in potency compared to EN460 (IC_50_ 2,6 μM versus 6 μM), both *in vitro* and *in vivo*. Although the precise mechanism underlying this improvement remains unclear, similar effects have been observed in other ortho-fluorinated compounds [50].

Despite efforts to lower the IC_50_ of EN460 derivatives, I29’s potency remains in the micromolar range—a relatively high for a covalent inhibitor. However, this may be acceptable, as other covalent inhibitors targeting Thioredoxin Reductase 1 also function in this range *in vivo* [51].

Notably, I29 was not only more potent in *in vitro* in the AUR kinetic assays of ERO1 activity but also demonstrated efficacy in cells and *in vivo*. It showed therapeutic benefit in two preclinical models: (1) triple-negative breast cancer (TNBC), an aggressive subtype where ERO1 is upregulated and involved in VEGF folding and immune modulation [12]; and (2) SEPN1-related myopathy (SEPN1-RM), a rare congenital disorder lacking effective treatments, where ERO1 overexpression contributes to maladaptive ER stress responses [26, 27]. In both models, I29 produced beneficial effects, indicating its promise as a therapeutic candidate. However, further investigation into the pharmacokinetic properties, dosing strategies, and tissue-specific engagement of ERO1 inhibitors is necessary to clarify their potential for translation to clinical settings.

Finally, targeting cysteine residues remains a widely validated and effective strategy in covalent enzyme inhibition. Recent FDA approvals of covalent drugs have dispelled concerns over electrophilic compounds’ promiscuity [52,53]. Thus, EN460 derivatives, represent a valuable addition to the pharmacological arsenal of cysteine-targeting covalent inhibitors, with a therapeutic potential for diseases involving ERO1 upregulation.

### 4.1. Limitations of the study

While the EN460 derivatives described in this work were developed with the primary goal of improving potency toward ERO1, we acknowledge that some of these compounds also display partial inhibitory activity against PDI, a functional partner of ERO1 in the oxidative protein folding pathway. Although the IC_50_ values for PDI inhibition are substantially higher than those observed for ERO1, this cross-reactivity represents a limitation of the present study. Future structure–activity optimization efforts will focus on enhancing the selectivity of these inhibitors toward ERO1 while minimizing off-target effects on PDI and/or Mao-A. Nevertheless, from a therapeutic standpoint, the potential dual inhibition of ERO1 and PDI may not be disadvantageous. Both enzymes are frequently co-upregulated in certain cancers and contribute to tumor progression; thus, simultaneous inhibition of these functionally coupled enzymes could produce synergistic effects and improve antitumor efficacy.

## Supplementary Material

Supplementary material

## Data Availability

Data will be made available on request.

## Appendix A. Supporting information

Supplementary data associated with this article can be found in the online version at doi:10.1016/j.phrs.2025.108037.
