# Supplementary material for: Pyrazolone-based ERO1 inhibitors in ERO1-driven triple-negative breast cancer and SEPN1-related myopathy: Structure–activity relationship and therapeutic potential
Source: Pharmacol Res. Author manuscript; Available in PMC 2026 May 24. (PMC13198932; doi:10.1016/j.phrs.2025.108037)

Table of Contents

1. **General information** **S2**
2. **Synthetic procedure and characterization data of inhibitors I_{24}-I_{34}** **S3**
3. **Synthetic procedure and characterization data of inhibitors I_{36}-I_{40}** **S15**
4. **Synthetic procedure and characterization data of inhibitor I_{35}** **S21**
5. **Synthetic procedure and characterization data of EN460 salts** **S22**
6. **References** **S25**
7. **Copies of ^{1}H NMR and ^{13}C NMR spectra** **S26**
8. **Supplementary figure legends and Figures**

Experimental Section

1. General information

All reactions were conducted in air unless otherwise noted. Column chromatography purifications were performed in flash chromatography conditions using Merck 230-400 mesh silica gel. Analytical thin layer chromatography (TLC) was carried out on Merck silica gel plates (Silica Gel 60 F254), that were visualized by exposure to ultraviolet light and an aqueous solution of $KMnO_4$. ^{1}H NMR and ^{13}C NMR spectra were recorded on a Bruker Avance 600 spectrometer using DMSO-d_6, $CDCl_3$ or CD_3OD as solvent. ^{1}H NMR chemical shifts (δ scale) are reported in parts per million (ppm) relative to the central peak of the solvent, DMSO-d_6 (δ = 2.50 ppm), $CDCl_3$ (δ = 7.26 ppm), CD_3OD (δ = 3.31 ppm). Data are reported as follows: chemical shift, multiplicity (s = singlet, brs = broad singlet, d = doublet, t = triplet, m = multiplet), coupling constants (Hz) and integration. ^{13}C NMR chemical shifts are reported in ppm (δ) relative to DMSO-d_6 (δ = 39.52 ppm), $CDCl_3$ (δ = 77.16 ppm), CD_3OD (δ = 49.0 ppm). Yields refer to isolated material. All the synthesized inhibitors tested (**I_{24}-I_{30}**) had purity greater than 95%, judged by ^{1}H NMR spectroscopy and high-pressure liquid chromatography. HPLC analyses were performed on a Waters HPLC/UV/MS system (separation module Alliance HT2795, photo diode array detector 2996, mass detector Micromass ZQ, using column Phenomenex C6-phenyl 150 mm × 4.60 mm × 5 mm. The mobile phase consisted of acetonitrile and water (e containing 0.1% formic acid). A linear gradient of 70% to 100% acetonitrile over 8 minutes was used with a 10-minute run time at a flow rate of 1 mL/min. High-resolution mass spectrometry (HRMS) analysis was performed using a Q-TOF micro TM mass spectrometer.
Starting materials: 4,4,4-trifluoroacetoacetate was purchased from the best-known commercial suppliers and used without further purification. All the aromatic hydrazines were purchased from the best-known commercial suppliers and used without further purification. All the aromatic aldehydes were purchased from the best-known commercial suppliers and used without further purification. All the other chemicals and solvents were purchased from Merck Sigma-Aldrich and used without further purification.

2. **Synthetic procedure and characterization data of inhibitors I_{24}-I_{34}**

Inhibitors **I_{24}-I_{34}** were prepared according to the previously reported [1] procedure depicted in **Scheme 1.**

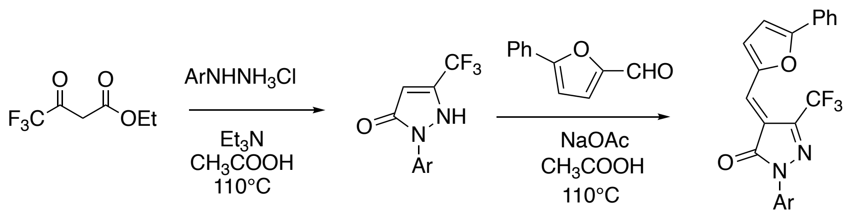

Scheme 1

To a solution of appropriate Hydrazine hydrochloride (1 equiv.) and 4,4,4-trifluoroacetoacetate (1 equiv.), in acetic acid (1.0 M), was added triethylamine (1 equiv.). The resulting mixture was heated at reflux for 16h. The excess of acetic acid was evaporated under reduced pressure, resulting in viscous oil. The crude product was partitioned between water and dichloromethane, the phases were separated, and the organic layer was dried with sodium sulfate and evaporated under reduced pressure to afford the corresponding pyrazolone that was used in the next step without further purification.

To a solution of the above obtained pyrazolone derivatives (1 equiv.) and 5-phenylfuran-2-carbaldehyde (1 equiv.), in acetic acid (0.5 M), was added a catalytic amount of sodium acetate (0.1 equiv). The resulting mixture was heated to reflux for 4 h and the excess of acetic acid was evaporated under reduced pressure and then co-evaporated with toluene (5 mL x3). The crude mixture was purified by crystallization with a mixture of water-acetone (5:1). The precipitate formed was collected by filtration, washed three times with water and dried.

(E)-2-(naphthalen-1-yl)-4-((5-phenylfuran-2-yl)methylene)-5-(trifluoromethyl)-2,4-dihydro-3H-pyrazol-3-one (I_{24})

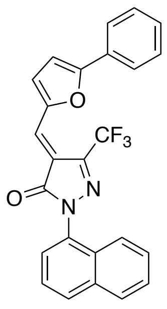

I_{24}

The reactions were carried out following the general procedure using 2-(naphthalen-1-yl)hydrazin-1-ium chloride (194.6 mg, 1 mmol) and 4,4,4-trifluoroacetoacetate (0.146 mL, 1 mmol, 1 equiv.) for the preparation of pyrazolone core, and using 5-phenylfuran-2-carbaldehyde (172.18 mg, 1 mmol, 1 equiv.) for the last step of inhibitor synthesis. The crude mixture was purified by column chromatography (cyclohexane : ethyl acetate 9:1) to afford the title compound **I_{24}** as a red solid with 70% Yield (302 mg, 0.7 mmol).

^{1}H NMR (600 MHz, $CDCl_3$) δ 9.04 (brs, 1H), 7.98-7.89 (m, 4H), 7.63 (s, 1H), 7.81-7.78 (m, 1H), 7.64-7.55 (m, 4H), 7.53-7.47 (m, 3H), 7.085 (d, J = 6 Hz).

^{13}C NMR (150 MHz, $CDCl_3$) δ 162.8, 162.5, 150.8, 140.2 (q, J = 60 Hz), 134.5, 133.0, 131.3, 130.6, 129.7, 129.6, 129.2, 128.5, 128.5, 128.4, 127.0, 126.5, 125.7, 125.2, 125.1, 124.2, 123.2, 120.1 (q, J = 405 Hz), 113.6, 111.7.

LC-MS retention time 2.27 min; LRMS m/z $[M+H]^+$: 433.4.

HRMS (ESI-TOF) m/z calcd. for $C_{25}H_{16}F_3N_2O_2$ $[M+H]^+$: 433.1158; found 433.1150.

(E)-4-((5-phenylfuran-2-yl)methylene)-2-(o-tolyl)-5-(trifluoromethyl)-2,4-dihydro-3H-pyrazol-3-one (I_{25})

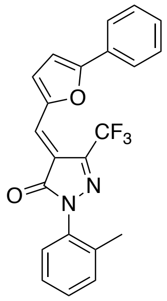

I_{25}

The reactions were carried out following the general procedure using 2-(o-tolyl)hydrazin-1-ium chloride (158.6 mg, 1 mmol) and 4,4,4-trifluoroacetoacetate (0.146 mL, 1 mmol, 1 equiv.) for the preparation of pyrazolone core, and using 5-phenylfuran-2-carbaldehyde (172.18 mg, 1 mmol, 1 equiv.) for the last step of inhibitor synthesis. The crude mixture was purified by column chromatography (cyclohexane : ethyl acetate 9:1) to afford the title compound $\mathbf{I_{25}}$ as a red solid with 50% Yield (198 mg, 0.5 mmol).

^{1}H NMR (600 MHz, $CDCl_3$) δ 9.03 (brs, 1H), 7.91-7.88 (m, 2H), 7.75 (s, 1H), 7.53-7.44 (m, 3H), 7.39-7.30 (m, 4H), 7.085 (d, J = 6 Hz), 2.31 (s, 3H).

^{13}C NMR (150 MHz, $CDCl_3$) δ 162.3, 162.1, 150.7, 139.8 (q, J = 45 Hz), 135.6, 131.2, 130.9, 130.5, 130.4, 130.2, 129.2, 129.0, 128.5, 127.2, 126.7, 125.7, 120.1 (q, J = 270 Hz), 113.8, 111.5, 18.3.

LC-MS retention time 5.57 min; LRMS m/z $[M+H]^+$: 397.4.

HRMS (ESI-TOF) m/z calcd. for $C_{22}H_{16}F_3N_2O_2$ $[M+H]^+$: 397.1158; found 397.1150.

(E)-2-(2,6-dimethylphenyl)-4-((5-phenylfuran-2-yl)methylene)-5-(trifluoromethyl)-2,4-dihydro-3H-pyrazol-3-one (I_{26})

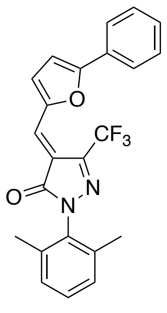

I_{26}

The reactions were carried out following the general procedure using 2-(2,6-dimethylphenyl)hydrazin-1-ium chloride (172.6 mg, 1 mmol) and 4,4,4-trifluoroacetoacetate (0.146 mL, 1 mmol, 1 equiv.) for the preparation of pyrazolone core, and using 5-phenylfuran-2-carbaldehyde (172.18 mg, 1 mmol, 1 equiv.) for the last step of inhibitor synthesis. The crude mixture was purified by column chromatography (cyclohexane : ethyl acetate 9:1) to afford the title compound **I_{26}** as a red solid with 70% Yield (287 mg, 0.7 mmol).

^{1}H NMR (600 MHz, DMSO-d$_6$) δ 8.75 (brs, 1H), 8.09-8.07 (m, 2H), 7.86 (s, 1H), 7.63-7.51 (m, 4H), 7.37-7.33 (m, 1H), 7.28-7.22 (m, 2H), 2.11 (s, 6H).

^{13}C NMR (150 MHz, DMSO-d$_6$) δ 162.7, 161.7, 150.6, 149.8, 139.4 (q, J = 45 Hz), 137.1, 135.0, 131.4, 130.2, 130.0, 129.8, 128.8, 128.6, 128.5, 126.3, 120.1 (q, J = 270 Hz), 118.5, 113.0, 112.7, 18.00.

LC-MS retention time 5.77 min; LRMS m/z $[M+H]^+$: 411.5.

HRMS (ESI-TOF) m/z calcd. for $C_{23}H_{18}F_3N_2O_2$ $[M+H]^+$: 411.1315; found 411.1309.

(E)-2-(2-chlorophenyl)-4-((5-phenylfuran-2-yl)methylene)-5-(trifluoromethyl)-2,4-dihydro-3H-pyrazol-3-one (I_{27})

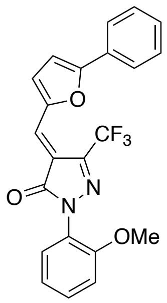

I_{27}

The reactions were carried out following the general procedure using 2-(2-methoxyphenyl)hydrazin-1-ium chloride (213.48 mg, 1 mmol) and 4,4,4-trifluoroacetoacetate (0.146 mL, 1 mmol, 1 equiv.) for the preparation of pyrazolone core, and using 5-phenylfuran-2-carbaldehyde (172.18 mg, 1 mmol, 1 equiv.) for the last step of inhibitor synthesis. The crude mixture was purified by crystallization with a mixture of water-acetone (5:1) and the precipitate formed was collected by filtration, washed three times with water and dried. The title compound **I_{27}** was isolated as a red solid with 64% Yield over two steps (263 mg, 0.64 mmol).

^{1}H NMR (600 MHz, DMSO-d$_6$) δ 8.71 (brs, 1H), 8.07-8.05 (m, 2H). 7.78 (s, 1H), 7.60-7.49 (m, 5H), 7.425 (dd, J = 12 Hz, 6 Hz,1H), 7.24 (d, J = 12 Hz, 1H), 7.11-7.07 (m, 1H), 3.80 (s, 3H).

^{13}C NMR (150 MHz DMSO-d$_6$) δ 162.3, 162.0, 155.7, 150.5, 138.4 (q, J = 45 Hz), 135.1, 131.4, 131.2, 129.80, 129.77, 129.7, 128.5, 126.2, 125.5, 121.0, 120.5 (q, J = 270 Hz), 113.1, 112.9, 109.3, 56.4.

LC-MS retention time 4.94 min; LRMS m/z $[M+H]^+$: 413.4.

HRMS (ESI-TOF) m/z calcd. for $C_{22}H_{16}F_3N_2O_3$ $[M+H]^+$: 413.1108; found 413.1116.

(E)-2-(2-chlorophenyl)-4-((5-phenylfuran-2-yl)methylene)-5-(trifluoromethyl)-2,4-dihydro-3H-pyrazol-3-one (I_{28})

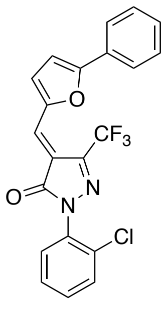

I_{28}

The reactions were carried out following the general procedure using 2-(2-chlorophenyl)hydrazin-1-ium chloride (179 mg, 1 mmol) and 4,4,4-trifluoroacetoacetate (0.146 mL, 1 mmol, 1 equiv.) for the preparation of pyrazolone core, and using 5-phenylfuran-2-carbaldehyde (172.18 mg, 1 mmol, 1 equiv.) for the last step of inhibitor synthesis. The crude mixture was purified by column chromatography (cyclohexane : ethyl acetate 9:1) to afford the title compound **I_{28}** as a red solid with 45% Yield (187 mg, 0.45 mmol).

^{1}H NMR (600 MHz, DMSO-d$_6$) δ 8.71 (brs, 1H), 8.08 (d, J = 12 Hz), 7.85 (s, 1H), 7.725 (dd, J = 6 Hz, 1 Hz,1H), 7.675 (dd, J = 6 Hz, 1 Hz,1H), 7.625 (d, J = 6 Hz, 1H), 7.60-7.53 (m, 5H).

^{13}C NMR (150 MHz, DMSO-d$_6$) δ 162.8, 150.5, 139.6 (q, *J* = 45 Hz), 134.5, 131.9, 131.7, 131.4, 130.8, 130.74, 130.70, 130.3, 129.9, 129.8, 128.7, 128.4, 126.3, 120.4 (q, *J* = 270 Hz), 113.08, 112.5.

LC-MS retention time 5.15 min; LRMS m/z $[M+H]^+$: 417.8.

HRMS (ESI-TOF) m/z calcd. for $C_{21}H_{13}ClF_3N_2O_2$ $[M+H]^+$: 417.0612; found 417.0619.

(E)-2-(2-fluorophenyl)-4-((5-phenylfuran-2-yl)methylene)-5-(trifluoromethyl)-2,4-dihydro-3H-pyrazol-3-one (I_{29})

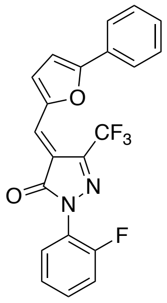

I_{29}

The reactions were carried out following the general procedure using 2-(2-fluorophenyl)hydrazin-1-ium chloride (162.6 mg, 1 mmol) and 4,4,4-trifluoroacetoacetate (0.146 mL, 1 mmol, 1 equiv.) for the preparation of pyrazolone core, and using 5-phenylfuran-2-carbaldehyde (172.18 mg, 1 mmol, 1 equiv.) for the last step of inhibitor synthesis. The crude mixture was purified by crystallization with a mixture of water-acetone (5:1) and the precipitate formed was collected by filtration, washed three times with water and dried. The title compound **I_{29}** was isolated as a red solid with 60% Yield over two steps (240 mg, 0.6 mmol).

^{1}H NMR (600 MHz, $CDCl_3$) δ 9.04 (brs, 1H), 7.92-7.88 (m, 2H), 7.76 (s, 1H), 7.55-7.44 (m, 4H), 7.43-7.39 (m, 1H), 7.30-7.29 (m, 1H), 7.26-7.24 (m, 1H), 7.095 (d, J = 6 Hz).

^{13}C NMR (150 MHz, $CDCl_3$) δ 162.6, 161.8, 157.7, 156.1, 150.7, 140.8 (q, *J* = 30 Hz), 131.3, 130.6, 130.1, 130.0, 129.2, 128.5, 127.8, 125.8, 124.6, 124.5, 120.1 (q, *J* = 270 Hz), 116.85 (d, *J* = 15 Hz), 113.2, 111.7.

LC-MS retention time 5.20 min; LRMS m/z $[M+H]^+$: 401.4.

HRMS (ESI-TOF) m/z calcd. for $C_{21}H_{13}F_4N_2O_2$ $[M+H]^+$: 401.0908; found 401.0915.

(E)-2-(2,5-difluorophenyl)-4-((5-phenylfuran-2-yl)methylene)-5-(trifluoromethyl)-2,4-dihydro-3H-pyrazol-3-one (I_{30})

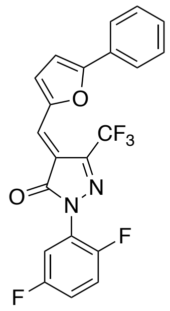

I_{30}

The reactions were carried out following the general procedure using 2-(2,5-difluorophenyl)hydrazin-1-ium chloride (180.6 mg, 1 mmol) and 4,4,4-trifluoroacetoacetate (0.146 mL, 1 mmol, 1 equiv.) for the preparation of pyrazolone core, and using 5-phenylfuran-2-carbaldehyde (172.18 mg, 1 mmol, 1 equiv.) for the last step of inhibitor synthesis. The crude mixture was purified by crystallization with a mixture of water-acetone (5:1) and the precipitate formed was collected by filtration, washed three times with water and dried. The title compound $\mathbf{I_{30}}$ was isolated as a red solid with 80% Yield over two steps (368 mg, 0.8 mmol).

^{1}H NMR (600 MHz, $CDCl_3$) δ 9.04 (brs, 1H), 7.91-7.89 (m, 2H), 7.76 (brs, 1H), 7.53-7.46 (m, 3H), 7.30 (ddd, J = 8.6 Hz, 5.7 Hz, 3.1 Hz, 1H), 7.23 (td, J = 9.3 Hz, 4.7 Hz, 1H), 7.12-7.06 (m, 2H).

^{13}C NMR (150 MHz, $CDCl_3$) δ 162.9, 161.5, 159.1, 157.5, 153.7, 152.1, 150.6, 141.3 (q, J = 45 Hz), 131.6, 130.9, 130.8, 129.3, 128.4, 125.8, 119.9 (q, J = 270 Hz), 117.65 (dd, J = 30 Hz, 15 Hz), 116.35 (dd, J = 30 Hz, 15 Hz), 114.35 (d, J = 15 Hz), 112.8, 111.8.

LC-MS retention time 2.27 min; LRMS m/z $[M+H]^+$: 419.3.

HRMS (ESI-TOF) m/z calcd. for $C_{21}H_{12}F_5N_2O_2$ $[M+H]^+$: 419.0813; found 419.0819.

(E)-3-(5-oxo-4-((5-phenylfuran-2-yl)methylene)-3-(trifluoromethyl)-4,5-dihydro-1H-pyrazol-1-yl)benzonitrile (I_{31})

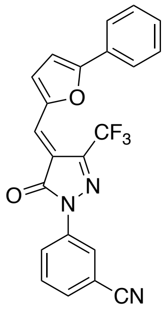

I_{31}

The reactions were carried out following the general procedure using 2-(3-cyanophenyl)hydrazin-1-ium chloride (169.6 mg, 1 mmol) and 4,4,4-trifluoroacetoacetate (0.146 mL, 1 mmol, 1 equiv.) for the preparation of pyrazolone core, and using 5-phenylfuran-2-carbaldehyde (172.18 mg, 1 mmol, 1 equiv.) for the last step of inhibitor synthesis.

The crude mixture was purified by crystallization with a mixture of water-acetone (5:1) and the precipitate formed was collected by filtration, washed three times with water and dried. The title compound **I_{31}** was isolated as a red solid with 60% Yield over two steps (244 mg, 0.6 mmol).

^{1}H NMR (600 MHz, DMSO-d$_6$) δ 8.83 (brs, 1H), 8.32 (s, 1H), 8.215 (d, J = 6 Hz, 1H), 8.1 (d, J = 12 Hz, 1H), 7.85 (s, 1H), 7.82-7.63 (m, 2H), 7.675 (d, J = 6 Hz, 1H), 7.62-7.53 (m, 3H).

^{13}C NMR (150 MHz, DMSO-d$_6$) δ 177.8, 172.1, 160.4, 158.2, 155.2, 151.7, 150.9, 139.4, 133.2, 130.6, 129.7, 129.2, 128.8, 128.5, 126.8, 125, 122.7, 120.9 (q, J = 270 Hz), 119.1, 118.1, 108.7, 107.6, 106.4, 98.0.

LC-MS retention time 5.75 min; LRMS m/z $[M+H]^+$: 408.4.

HRMS (ESI-TOF) m/z calcd. for $C_{22}H_{13}F_3N_3O_2$ $[M+H]^+$: 408.0954; found 408.0948.

(E)-4-((5-phenylfuran-2-yl)methylene)-2-(pyridin-3-yl)-5-(trifluoromethyl)-2,4-dihydro-3H-pyrazol-3-one (I_{32})

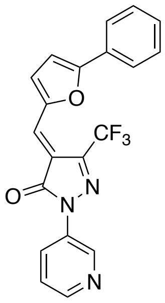

I_{32}

The reactions were carried out following the general procedure using 3-hydrazineylpyridine (109.1 mg, 1 mmol) and 4,4,4-trifluoroacetoacetate (0.146 mL, 1 mmol, 1 equiv.) for the preparation of pyrazolone core, and using 5-phenylfuran-2-carbaldehyde (172.18 mg, 1 mmol, 1 equiv.) for the last step of inhibitor synthesis.

The crude mixture was purified by column chromatography (cyclohexane : ethyl acetate 9:1) to afford the title compound **I_{32}** as a red solid with 64% Yield (245 mg, 0.64 mmol).

^{1}H NMR (600 MHz, DMSO-d$_6$) δ 9.125 (d, J = 6 Hz), 8.81 (brs, 1H), 8.545 (dd, J = 2, 7 Hz, 1H), 8.26-8.23 (m, 1H), 8.12-8.10 (m, 2H), 7.86 (s, 1H), 7.665 (d, J = 6 Hz, 1H), 7.64-7.53 (m, 4H).

^{13}C NMR (150 MHz, DMSO-d$_6$) δ 163.1, 161.6, 150.5, 147.4, 141.5, 140.6, 134.6, 131.5, 130.5, 130.0, 129.9, 128.4, 128.39, 127.6, 126.4, 124.5, 123.3, 120.3 (q, J = 270 Hz), 113.3, 113.27.

LC-MS retention time 4.84 min; LRMS m/z $[M+H]^+$: 384.4.

HRMS (ESI-TOF) m/z calcd. for $C_{20}H_{13}F_3N_3O_2$ $[M+H]^+$: 384.0954; found 384.0948.

(E)-3-(5-oxo-4-((5-phenylfuran-2-yl)methylene)-3-(trifluoromethyl)-4,5-dihydro-1H-pyrazol-1-yl)benzenesulfonamide (I_{33})

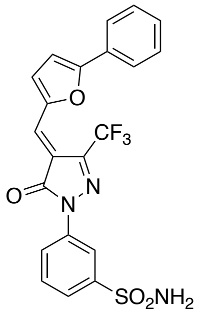

I_{33}

The reactions were carried out following the general procedure using 3-hydrazineylbenzenesulfonamide (187.2 mg, 1 mmol) and 4,4,4-trifluoroacetoacetate (0.146 mL, 1 mmol, 1 equiv.) for the preparation of pyrazolone core, and using 5-phenylfuran-2-carbaldehyde (172.18 mg, 1 mmol, 1 equiv.) for the last step of inhibitor synthesis.

The crude mixture was purified by crystallization with a mixture of water-acetone (5:1) and the precipitate formed was collected by filtration, washed three times with water and dried. The title compound **I_{33}** was isolated as a red solid with 60% Yield over two steps (277 mg, 0.6 mmol).

^{1}H NMR (600 MHz, DMSO-d$_6$) δ 8.85 (brs, 1H), 8.37-8.36 (m, 1H), 8.18-8.15 (m, 1H), 8.11-8.08 (m, 2H), 7.85 (s, 1H), 7.77-7.60 (m, 2H), 7.665 (d, J = 2 Hz, 1H), 7.61-7.54 (m, 3H), 7.52 (brs, 2H).

^{13}C NMR (150 MHz, DMSO-d$_6$) δ 163.1, 161.5, 150.5, 145.5, 140.15 (q, J = 45 Hz), 138.2, 132.6, 131.5, 130.5, 130.4, 129.9, 128.4, 126.4, 123.4, 122.6, 120.4 (q, J = 270 Hz), 116.8, 113.5, 113.4.

LC-MS retention time 4.00 min; LRMS m/z $[M+H]^+$: 462.4.

HRMS (ESI-TOF) m/z calcd. for $C_{21}H_{15}F_3N_3O_4S$ $[M+H]^+$: 462.0730; found 462.0737.

(E)-3-(5-oxo-4-((5-phenylfuran-2-yl)methylene)-3-(trifluoromethyl)-4,5-dihydro-1H-pyrazol-1-yl)benzenesulfonic acid (I_{34})

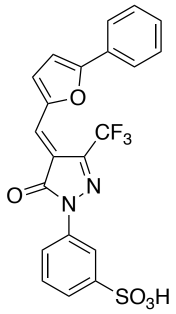

I_{34}

The reactions were carried out following the general procedure using 3-hydrazineylbenzenesulfonic acid (188.2 mg, 1 mmol) and 4,4,4-trifluoroacetoacetate (0.146 mL, 1 mmol, 1 equiv.) for the preparation of pyrazolone core, and using 5-phenylfuran-2-carbaldehyde (172.18 mg, 1 mmol, 1 equiv.) for the last step of inhibitor synthesis.

The crude mixture was purified by trituration with dichloromethane and the precipitate formed was collected by filtration, washed three times with the same solvent and dried. The title compound **I_{34}** was isolated as a red solid with 67% Yield over two steps (309 mg, 0.67 mmol).

^{1}H NMR (600 MHz, DMSO-d$_6$) δ 8.87 (brs, 1H), 8.14-8.13 (m, 1H), 8.11-8.08 (m, 2H), 7.89-7.86 (m, 1H), 7.82 (s, 1H), 7.67-7.64 (m, 1H), 7.62-7.53 (m, 4H), 7.50-7.45 (m, 1H).,

^{13}C NMR (150 MHz, DMSO-d$_6$) δ 162.8, 161.4, 150.5, 149.9, 139.55 (q, J = 45 Hz), 137.4, 132.2, 131.4, 130.2, 129.8, 129.0, 128.5, 126.3, 123.6, 120.45 (q, J = 270 Hz), 119.9, 117.4, 113.9, 113.2.

LC-MS retention time 4.1 min; LRMS m/z $[M+H]^+$: 463.3.

HRMS (ESI-TOF) m/z calcd. for $C_{21}H_{14}F_3N_2O_5S$ $[M+H]^+$: 463.0570; found 463.0578.

3. Synthetic procedure and characterization data of inhibitors I_{36}-I_{40}

Inhibitors **I_{36}-I_{40}** were prepared according to the previously reported[1] procedure depicted in **Scheme 2.**

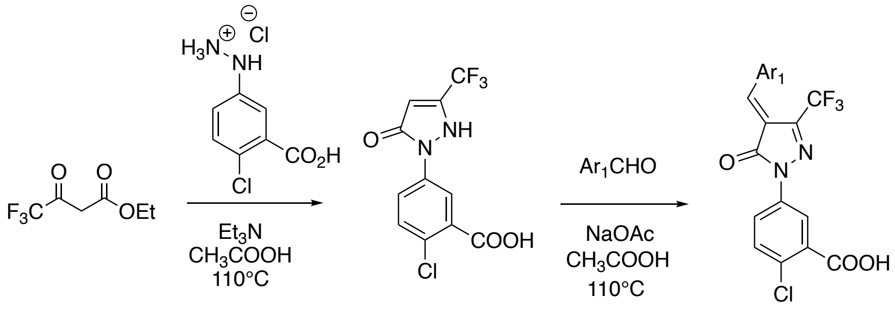

1

Scheme 2

STEP1: preparation of intermediate 1

To a solution of 2-chloro-5-hydrazinobenzoic acid hydrochloride (2235 mg, 10 mmol) and 4,4,4-trifluoroacetoacetate (1.46 mL, 10 mmol, 1 equiv.), in acetic acid (1.0 M), was added triethylamine (1 equiv.). The resulting mixture was heated at reflux for 16h. The excess of acetic acid was evaporated under reduced pressure, resulting in viscous oil. The crude product was partitioned between water and dichloromethane, the phases were separated, and the organic layer was dried with sodium sulfate and evaporated under reduced pressure to afford 2-chloro-5-(5-oxo-3-(trifluoromethyl)-2,5-dihydro-1H-pyrazol-1-yl) benzoic acid **1** (2140 mg, 7 mmol, 70% yield), that was used as a common intermediate for the preparations of inhibitors **I_{36}-I_{40}** without further purification.

STEP2: general procedure for the preparation of inhibitors I_{36}-I_{40}

To a solution of the above obtained 2-chloro-5-(5-oxo-3-(trifluoromethyl)-2,5-dihydro-1H-pyrazol-1-yl)benzoic acid **1** (1 equiv.) and the appropriate aldehyde (1 equiv.), in acetic acid (0.5 M), was added a catalytic amount of sodium acetate (0.1 equiv). The resulting mixture was heated to reflux for 4 h and the excess of acetic acid was evaporated under reduced pressure and then co-evaporated with toluene (5 mL x3). The crude mixture was purified by crystallization with a mixture of water-acetone (5:1). The precipitate formed was collected by filtration, washed three times with water and dried.

(E)-2-chloro-5-(4-((5-(3-fluorophenyl)furan-2-yl)methylene)-5-oxo-3-(trifluoromethyl)-4,5-dihydro-1H-pyrazol-1-yl)benzoic acid (I_{36})

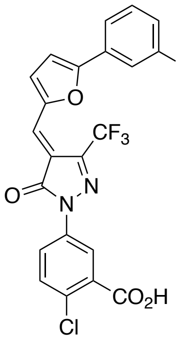

I_{36}

The reactions were carried out following the general procedure, using 2-chloro-5-(5-oxo-3-(trifluoromethyl)-2,5-dihydro-1H-pyrazol-1-yl)benzoic acid **1** (153.3mg, 0.5 mmol, 1 equiv.) and 5-(3-fluorophenyl)furan-2-carbaldehyde (95.6, 0.5 mmol, 1 equiv.).

The crude mixture was purified by crystallization with a mixture of water-acetone (5:1) and the precipitate formed was collected by filtration, washed three times with water and dried. The title compound $\mathbf{I_{36}}$ was isolated as a red solid with 59% Yield over two steps (282 mg, 0.59 mmol).

^{1}H NMR (600 MHz, DMSO-d$_6$) δ 13.66 (brs, 1H), 8.78 (brs, 1H), 8.355 (d, J = 6 Hz, 1H), 8.05 (dd, J = 12 Hz, 6 Hz, 1H), 7.96-7.92 (m, 2H), 7.86 (s, 1H), 7.74-7.59 (m, 3H), 7.41-7.36 (m, 1H).

^{13}C NMR (150 MHz, DMSO-d$_6$) δ 166.5, 163.8, 162.2, 161.3, 161.2, 150.6, 140.1 (q, *J* = 45 Hz), 136.7, 132.1, 132.0, 130.6, 130.5, 128.9, 123.2, 122.4, 121.5, 120.2 (q, *J* = 270 Hz), 118.1 (d, *J* = 30 Hz), 114.2, 114.0, 112.85 (d, *J* = 15 Hz), 110.4.

LC-MS retention time 5.47 min; LRMS m/z $[M+H]^+$: 479.8.

HRMS (ESI-TOF) m/z calcd. for $C_{22}H_{12}ClF_4N_2O_4$ $[M+H]^+$: 479.0416; found 479.0420.

(E)-2-chloro-5-(5-oxo-3-(trifluoromethyl)-4-((5-(3-(trifluoromethyl)phenyl)furan-2-yl)methylene)-4,5-dihydro-1H-pyrazol-1-yl)benzoic acid (I_{37})

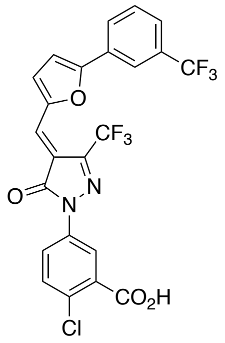

I_{37}

The reactions were carried out following the general procedure, using 2-chloro-5-(5-oxo-3-(trifluoromethyl)-2,5-dihydro-1H-pyrazol-1-yl)benzoic acid **1** (153.3mg, 0.5 mmol, 1 equiv.) and 5-(3-(trifluoromethyl)phenyl)furan-2-carbaldehyde (120.09, 0.5 mmol, 1 equiv.).

The crude mixture was purified by crystallization with a mixture of water-acetone (5:1) and the precipitate formed was collected by filtration, washed three times with water and dried. The title compound $\mathbf{I_{37}}$ was isolated as a red solid with 47% Yield over two steps (248 mg, 0.47 mmol).

^{1}H NMR (600 MHz, DMSO d_6) δ 13.64 (s, 1H), 8.78 (brs, 1H), 8.41 (s, 1H), 8.36-8.33 (m, 1H), 8.03-8.01 (m, 1H), 7.89-7.84 (m, 2H), 7.81-7.77 (m, 2H), 7.70-7.66 (m, 1H), 7.26-7.23 (m, 1H), 7.18-7.13 (m, 1H).

^{13}C NMR (150 MHz, DMSO-d_6) δ 178.7, 166.5, 161.3, 160.7, 158.8, 156.7, 155.9, 152.6, 150.83, 140.1 (q, J = 45 Hz), 136.7, 132.1, 132.0, 131.0, 130.9, 130.8, 129.9, 129.4, 128.9, 127.4, 126.4, 125.2, 124.4, 123.2, 121.8, 121.5, 121.1, 120.4, 114.4 (d, J = 51 Hz), 110.8.

LC-MS retention time 5.84 min; LRMS m/z $[M+H]^+$: 529.8.

HRMS (ESI-TOF) m/z calcd. for $C_{23}H_{12}ClF_6N_2O_4$ $[M+H]^+$: 529.0384; found 529.0377.

(E)-2-chloro-5-(4-((5-(4-fluorophenyl)furan-2-yl)methylene)-5-oxo-3-(trifluoromethyl)-4,5-dihydro-1H-pyrazol-1-yl)benzoic acid (I_{38})

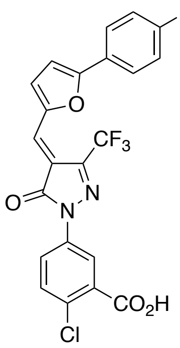

I_{38}

The reactions were carried out following the general procedure, using 2-chloro-5-(5-oxo-3-(trifluoromethyl)-2,5-dihydro-1H-pyrazol-1-yl)benzoic acid **1** (153.3mg, 0.5 mmol, 1 equiv.) and 5-(4-fluorophenyl)furan-2-carbaldehyde (95.6, 0.5 mmol, 1 equiv.).

The crude mixture was purified by crystallization with a mixture of water-acetone (5:1) and the precipitate formed was collected by filtration, washed three times with water and dried. The title compound **I_{38}** was isolated as a red solid with 51% Yield over two steps (244 mg, 0.51 mmol).

^{1}H NMR (600 MHz, DMSO-d_6) δ 13.65 (brs, 1H), 8.80 (brs, 1H), 8.335 (d, J = 6 Hz, 1H), 8.15-8.13 (m, 2H), 8.045 (dd, J = 12 Hz, 4 Hz, 1H), 7.81 (s, 1H), 7.68 (d, J = 12 Hz, 1H), 7.62 (d, J = 4 Hz, 1H), 7.43 (t, J = 6 Hz, 2H).

^{13}C NMR (150 MHz, DMSO-d_6) δ 166.5, 164.8, 163.2, 162.1, 161.4, 150.5, 140.1 (q, *J* = 45 Hz), 136.7, 132.1, 132.0, 130.5, 128.9, 128.9, 125.1, 123.2, 123.0, 121.5, 120.3 (q, *J* = 270 Hz), 117.07 (d, *J* = 21 Hz), 113.31 (d, *J* = 30 Hz).

LC-MS retention time 5.45 min; LRMS LRMS m/z $[M+H]^+$: 479.9.

HRMS (ESI-TOF) m/z calcd. for $C_{22}H_{12}ClF_4N_2O_4$ $[M+H]^+$: 479.0416; found 479.0409.

(E)-2-chloro-5-(4-((5-(3-methoxyphenyl)furan-2-yl)methylene)-5-oxo-3 (trifluoromethyl)-4,5-dihydro-1H-pyrazol-1-yl)benzoic acid (I_{39})

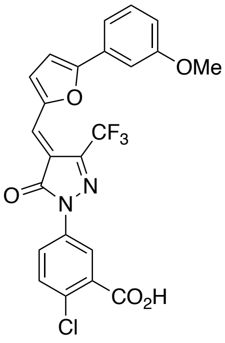

I_{39}

The reactions were carried out following the general procedure, using 2-chloro-5-(5-oxo-3-(trifluoromethyl)-2,5-dihydro-1H-pyrazol-1-yl)benzoic acid **1** (153.3mg, 0.5 mmol, 1 equiv.) and 5-(3-methoxyphenyl)furan-2-carbaldehyde (101.1, 0.5 mmol, 1 equiv.).

The crude mixture was purified by crystallization with a mixture of water-acetone (5:1) and the precipitate formed was collected by filtration, washed three times with water and dried. The title compound $\mathbf{I_{39}}$ was isolated as a red solid with 63% Yield over two steps (309 mg, 0.63 mmol).

^{1}H NMR (600 MHz, DMSO-d_6) δ 13.63 (brs, 1H), 8.78 (brs, 1H), 8.395 (d, J = 6 Hz, 1H), 8.04 (dd, J = 12 Hz, 6 Hz, 1H), 7.84 (s, 1H), 7.71-7.66 (m, 4H), 7.49 (t, J = 12 Hz, 1H), 7.13-7.10 (m, 1H), 3.89 (s, 3H).

^{13}C NMR (150 MHz, DMSO-d_6) δ 166.6, 163.0, 161.4, 160.4, 150.5, 136.8, 132.1, 132.0, 131.0, 130.9, 129.67, 128.9, 128.5, 123.3, 121.5, 118.9, 117.8, 116.1, 113.6, 113.5, 111.0, 109.6, 55.9.

LC-MS retention time 5.44 min; LRMS m/z $[M+H]^+$: 491.8.

HRMS (ESI-TOF) m/z calcd. for $C_{23}H_{15}ClF_3N_2O_5$ $[M+H]^+$: 491.0616; found 491.0620.

(E)-2-chloro-5-(5-oxo-4-((5-phenylthiophen-2-yl)methylene)-3-(trifluoromethyl)-4,5-dihydro-1H-pyrazol-1-yl)benzoic acid (I_{40})

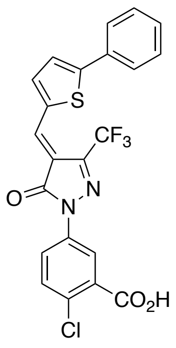

I_{40}

The reactions were carried out following the general procedure, using 2-chloro-5-(5-oxo-3-(trifluoromethyl)-2,5-dihydro-1H-pyrazol-1-yl)benzoic acid **1** (153.3mg, 0.5 mmol, 1 equiv.) and 5-phenylthiophene-2-carbaldehyde (94.1, 0.5 mmol, 1 equiv.).

The crude mixture was purified by crystallization with a mixture of water-acetone (5:1) and the precipitate formed was collected by filtration, washed three times with water and dried. The title compound $\mathbf{I_{40}}$ was isolated as a red solid with 47% Yield over two steps (248 mg, 0.47 mmol).

^{1}H NMR (600 MHz, DMSO-d$_6$) δ 13.59 (brs, 1H), 8.415 (d, J = 6 Hz, 1H), 8.20 (d, J = 6 Hz, 1H), 8.27 (s, 1H), 7.98 (dd, J = 12 Hz, 6 Hz, 1H), 7.88-7.84 (m, 3H), 7.61 (d, J = 12 Hz, 1H), 7.49-7.42 (m, 3H).

^{13}C NMR (150 MHz, DMSO-d$_6$) δ 166.6, 161.9, 160.6, 149.0, 140.8, 140.35 (q, J = 45 Hz), 136.7, 136.0, 132.8, 132.2, 132.0, 130.9, 130.0, 128.8, 127.2, 126.7, 123.0, 120.3 (q, J = 270 Hz), 113.8.

LC-MS retention time 5.40 min; LRMS m/z $[M+H]^+$: 477.4.

HRMS (ESI-TOF) m/z calcd. for $C_{22}H_{13}ClF_3N_2O_3S$ $[M+H]^+$: 477.0282; found 477.0275.

4. **Synthetic procedure and characterization data of inhibitor I_{35}**

Acetic acid, (E)-5-(3-(5-oxo-4-((5-phenylfuran-2-yl)methylene)-3-(trifluoromethyl)-4,5-dihydro-1H-pyrazol-1-yl)phenyl)-1H-tetrazol-1-ium salt (I_{35})

Inhibitor **I_{35}** was prepared according to reactions depicted in **Scheme 3.**

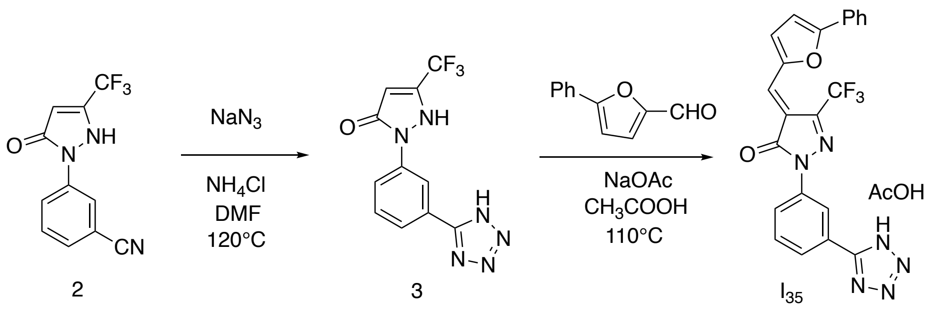

Scheme 3

To a solution pyrazolone **2**, obtained above for the synthesis of **I_{31}** (126.5 mg, 0.5 mmol, 1 equiv.) in DMF (5 ml) was added sodium azide (65 mg, 1 mmol, 2 equiv.) and ammonium chloride (26.5 mg, 0.5 mmol, 1 equiv.). The resulting mixture was heated at 120°C for 16 h. The reaction mixture was then cooled to room temperature, cold water (20 mL) was added and acidified with 2M HCl. The mixture was then extracted with EtOAc (3 x 20 mL) and the combined organic layers were washed with brine, dried over sodium sulfate, filtered and concentrated. The crude mixture was purified by column chromatography (acetonitrile : H_2O 95:5) to afford intermediate **3** with 50% Yield (74 mg, 0.25 mmol).

To a solution of the above obtained pyrazolone derivatives **3** (74 mg, 0.25 mmol, 1 equiv.) and 5-phenylfuran-2-carbaldehyde (34.4 mg, 0.2 mmol, 0.8 equiv.), in acetic acid (0.5 mL), was added a catalytic amount of sodium acetate (2 mg, 0.025 mmol, 0.1 equiv). The resulting mixture was heated to reflux for 4 h and the excess of acetic acid was evaporated under reduced pressure and then co-evaporated with toluene (5 mL x3). The crude mixture was purified by trituration with dichloromethane to afford title compound **I_{35}** as a red solid with 90% Yield (103 mg, 0.225 mmol).

^{1}H NMR (600 MHz, DMSO-d_6) δ 12.01 (brs, 1H), 8.89 (brs, 1H), 8.50-8.49 (m, 1H), 8.11-8.08 (m, 2H), 7.94-7.91 (m, 1H), 7.83-7.80 (m, 2H), 7.655 (d, J = 6 Hz, 1H), 7.62-7.50 (m, 4H), 1.92 (s, 3H).

^{13}C NMR (150 MHz, DMSO-d_6) δ 178.3, 172.7, 160.9, 158.7, 155.7, 152.2, 151.5, 139.9, 133.7, 131.1, 130.2, 129.7, 129.3, 129.0, 127.3, 125.5, 123.3, 121.1 (q, J = 270 Hz), 119.6, 118.5, 109.3, 108.1, 106.9, 98.5, 21.7.

LC-MS retention time 5.55 min; LRMS m/z $[M+H]^+$: 451.4.

HRMS (ESI-TOF) m/z calcd. for $C_{22}H_{14}F_3N_6O_2$ $[M+H]^+$: 451.1125; found 451.1131.

5. **Synthetic procedure and characterization data of EN460 salts**

EN460 salts were prepared according to the reaction depicted in **Scheme 4**.

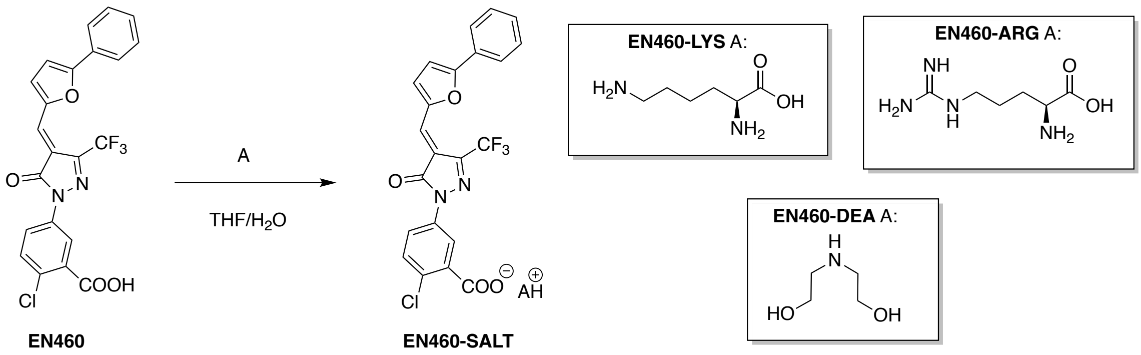

Scheme 4

General procedure for the preparation of EN460 salts

To a solution of **EN460** (1 equiv.) in THF : H_2O 10:1 (0.05 M) the appropriate base **A** (1 equiv.) was added and the resulting mixture was stirred at room temperature for 1 h. The precipitate formed was collected by filtration, washed three times with THF and dried.

(S)-5-amino-5-carboxypentan-1-aminium (E)-2-chloro-5-(5-oxo-4-((5-phenylfuran-2-yl)methylene)-3-(trifluoromethyl)-4,5-dihydro-1H-pyrazol-1-yl)benzoate (EN460-LYS)

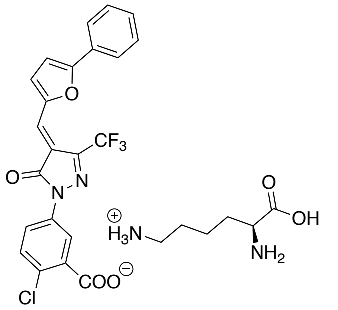

EN460-LYS

The reaction was carried out following the general procedure using **EN460** (50 mg, 0.11 mmol, 1 equiv.) and L-Lysine (16 mg, 0.11 mmol, 1 equiv.) The precipitate formed was collected by filtration, washed three times with THF and dried to obtain **EN460-LYS** (30 mg, 0.05 mmol, 45% Yield).

^{1}H NMR (600 MHz, CD_3OD) δ 8.99 (brs, 1H), 8.01 (d, J = 12 Hz, 2H), 7.91 (dd, J = 12 Hz, J = 6 Hz, 1H), 7.78 (s, 1H), 7.55-7.50 (m, 3H), 7.455 (d, J = 12 Hz, 1H), 7.375 (d, J = 6 Hz, 1H), 3.55 (t, J = 12 Hz, 1H), 2.93 (t, J = 12 Hz, 2H), 1.87-1.83 (m, 2H), 1.73-1.65 (m, 2H), 1.59-1.44 (m, 2H).

(S)-1-(4-amino-4-carboxybutyl)guanidinium (E)-2-chloro-5-(5-oxo-4-((5-phenylfuran-2-yl)methylene)-3-(trifluoromethyl)-4,5-dihydro-1H-pyrazol-1-yl)benzoate (EN460-ARG)

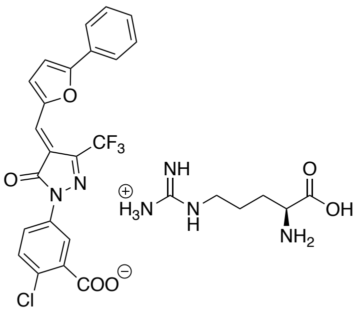

EN460-ARG

The reaction was carried out following the general procedure using **EN460** (50 mg, 0.11 mmol, 1 equiv.) and L-Arginine (19.1 mg, 0.11 mmol, 1 equiv.) The precipitate formed was collected by filtration, washed three times with THF and dried to obtain **EN460-ARG** (50 mg, 0.078 mmol, 72% Yield).

^{1}H NMR (600 MHz, CD_3OD) δ 8.99 (brs, 1H), 8.165 (d, J = 6 Hz, 1H), 8.05 (d, J = 6 Hz, 2H), 7.93 (dd, J = 12 Hz, J = 6 Hz, 1H), 7.78 (s, 1H), 7.57-7.45 (m, 4H), 7.375 (d, J = 6 Hz, 1H), 7.375 (d, J = 6 Hz, 1H), 3.60 (t, J = 12 Hz, 1H), 3.25-3.21 (m, 2H), 1.93-1.91 (m, 2H), 1.80-1.69 (m, 2H).

Bis(2-hydroxyethyl)ammonium (E)-2-chloro-5-(5-oxo-4-((5-phenylfuran-2-yl)methylene)-3-(trifluoromethyl)-4,5-dihydro-1H-pyrazol-1-yl)benzoate (EN460-DEA)

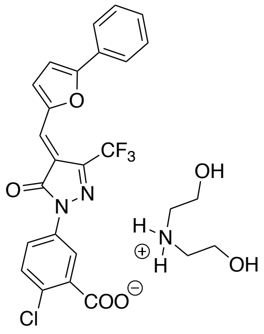

EN460-DEA

The reaction was carried out following the general procedure using **EN460** (50 mg, 0.11 mmol, 1 equiv.) and Diethanolamine (11.5 mg, 0.11 mmol, 1 equiv.) The precipitate formed was collected by filtration, washed three times with THF and dried to obtain **EN460-DEA** (35 mg, 0.062 mmol, 56% Yield).

^{1}H NMR (600 MHz, CD_3OD) δ 8.83 (brs, 1H), 8.145 (d, J = 6 Hz, 1H), 8.08 (d, J = 12 Hz, 2H), 7.91-7.88 (m, 1H), 7.80 (brs, 1H), 7.635 (d, J = 6 Hz, 1H), 7.60-7.53 (m, 4H), 3.68 (t, J = 6 Hz, 4H), 3.02 (t, J = 6 Hz, 4H).

6. References

[1] E. Varone, M. Retini, A. Cherubini, A. Chernorudskiy, A. Marrazza, A. Guidarelli, A. Cagnotto, M. Beeg, M. Gobbi, S. Fumagalli, M. Bolis, L. Guarrera, M. C. Barbera, C. Grasselli, A. Bleve, D. Generali, M. Milani, M. Mari, M. Salmona, G. Piersanti, G. Bottegoni, M. Broggini, Y. MW Janssen-Heininger, J. Cho, O. Cantoni, E. Zito; Small molecule-mediated inhibition of the oxidoreductase ERO1A restrains aggressive breast cancer by impairing VEGF and PD-L1 in the tumor microenvironment; *Cell Death and Disease,* **2025**, 16, 105.

7. NMR spectra

^{1}H-NMR and ^{13}C-NMR of (E)-2-(naphthalen-1-yl)-4-((5-phenylfuran-2-yl)methylene)-5-(trifluoromethyl)-2,4-dihydro-3H-pyrazol-3-one (I_{24})

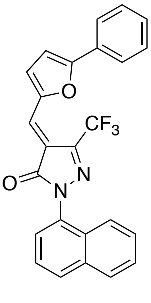

I_{24}

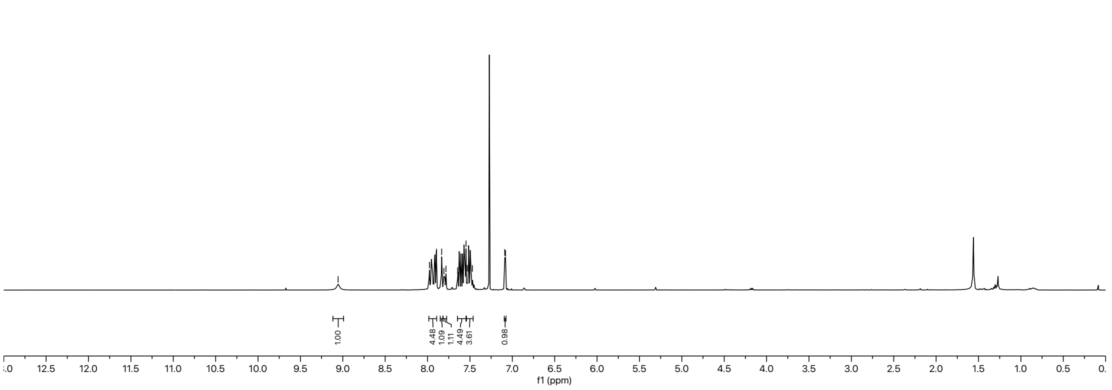

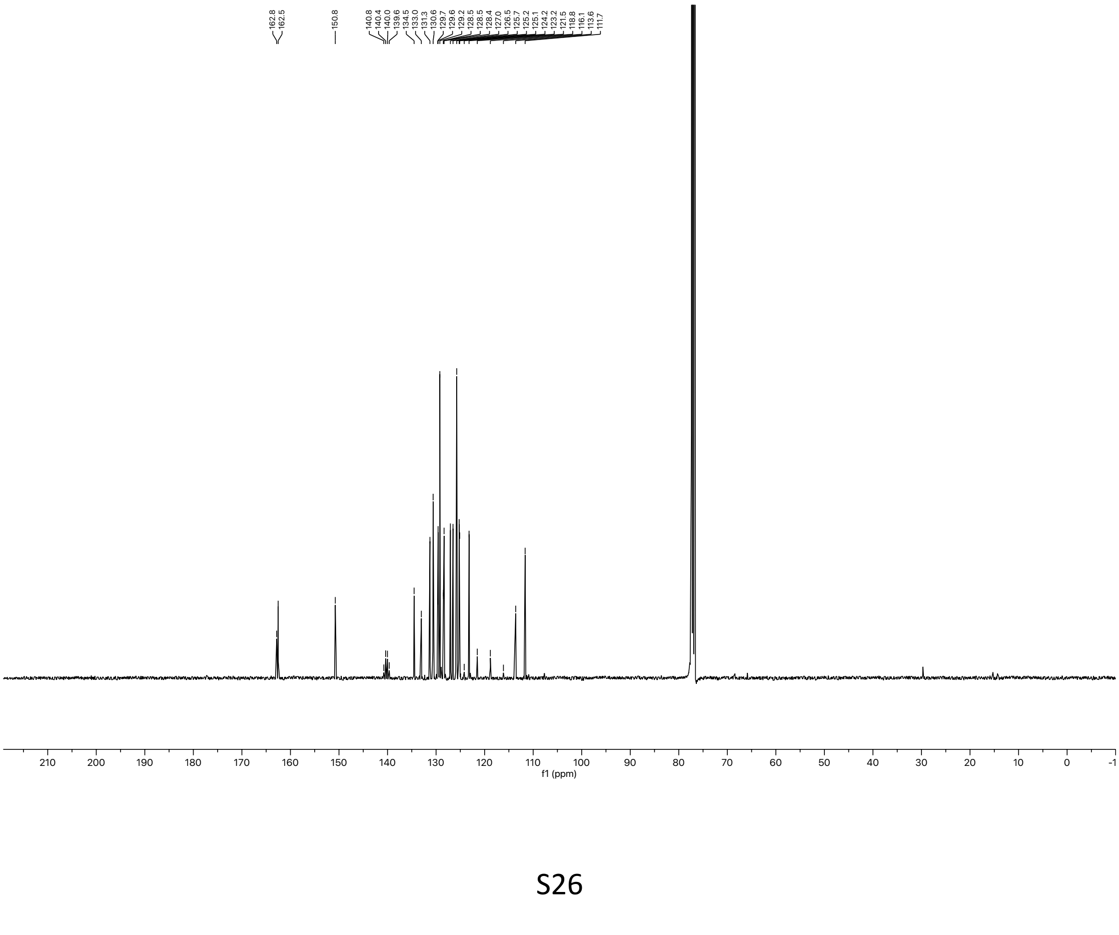

^{1}H-NMR and ^{13}C-NMR of (E)-2-(naphthalen-1-yl)-4-((5-phenylfuran-2-yl)methylene)-5-(trifluoromethyl)-2,4-dihydro-3H-pyrazol-3-one (I_{25})

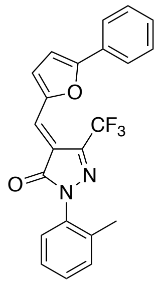

I_{25}

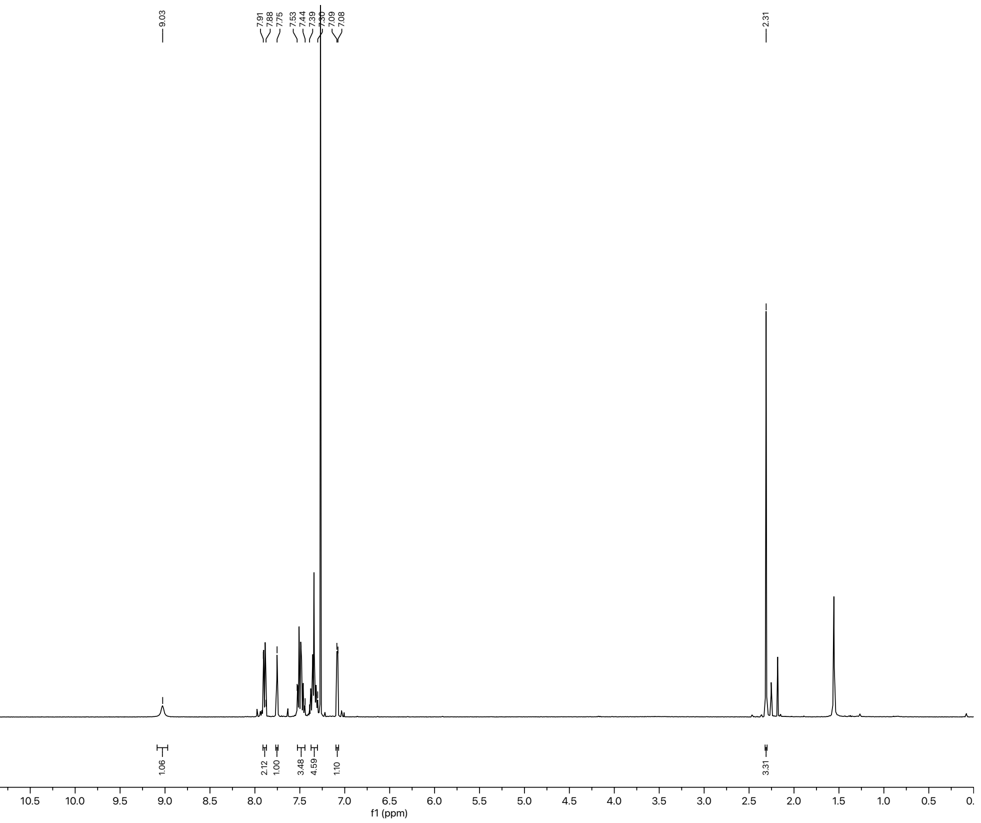

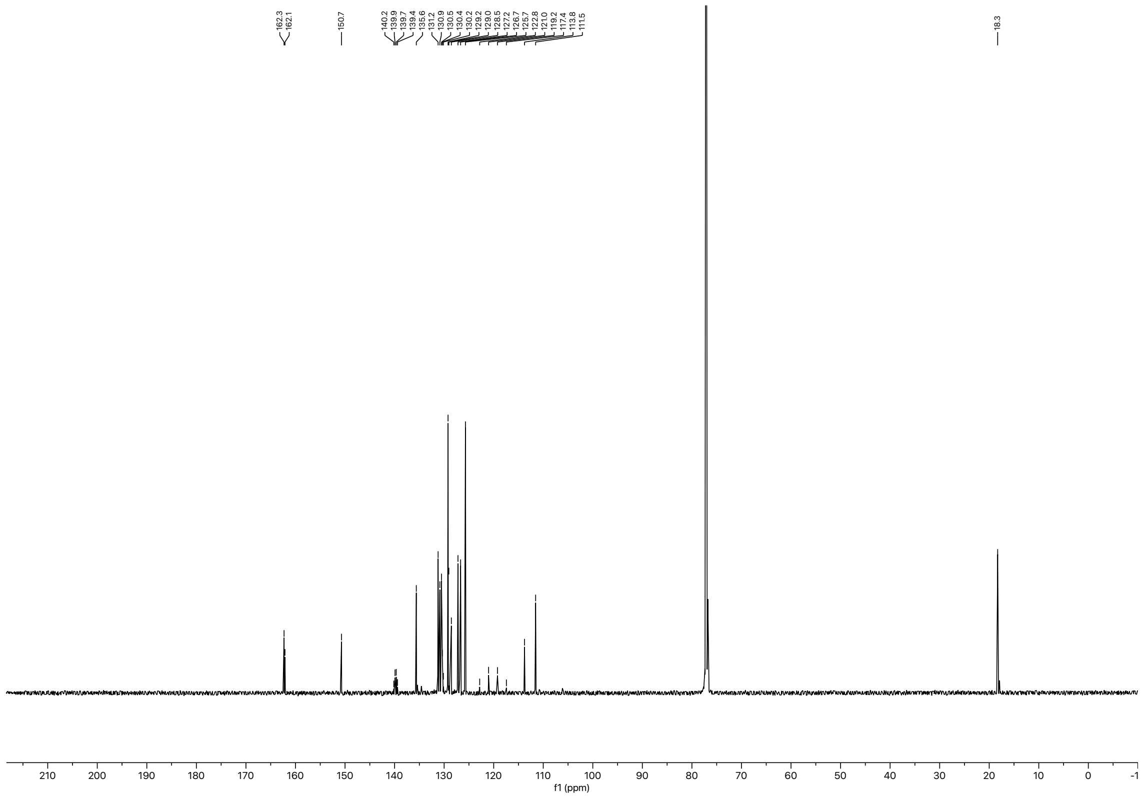

^{1}H-NMR and ^{13}C-NMR of (E)-2-(2,6-dimethylphenyl)-4-((5-phenylfuran-2-yl)methylene)-5-(trifluoromethyl)-2,4-dihydro-3H-pyrazol-3-one (I_{26})

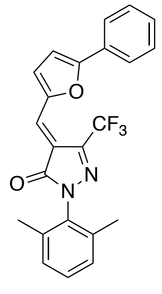

I_{26}

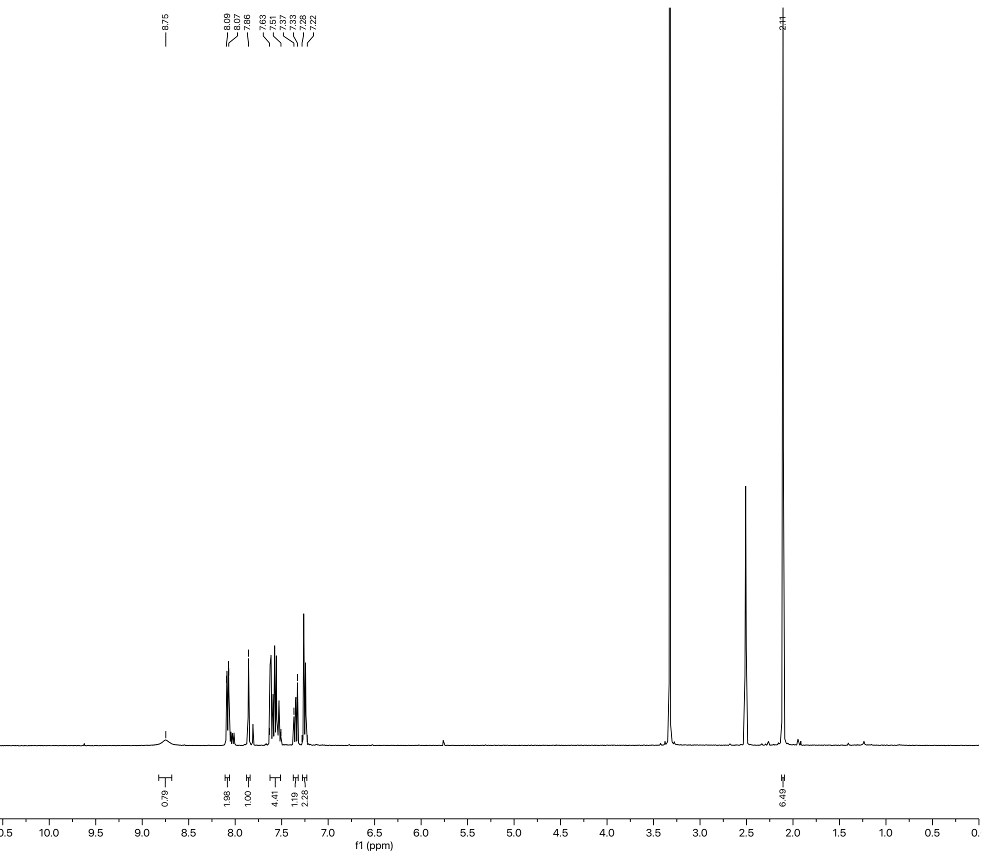

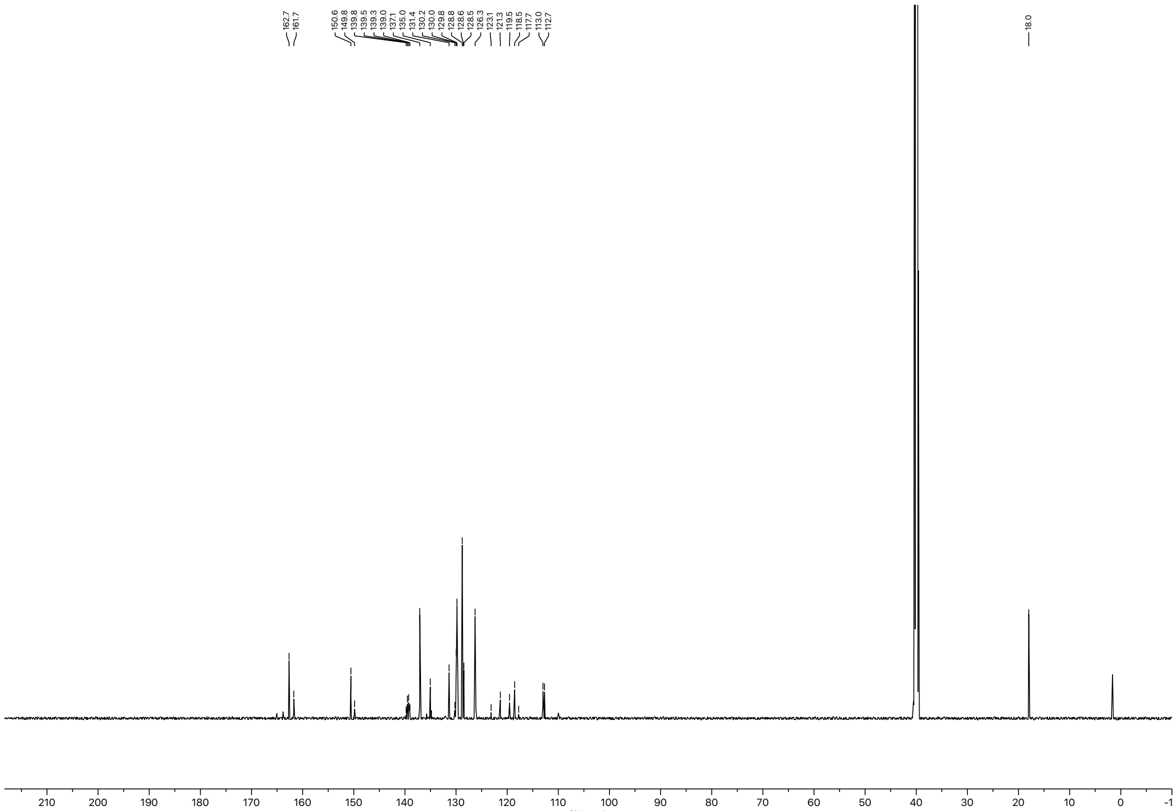

^{1}H-NMR and ^{13}C-NMR of (E)-2-(2-chlorophenyl)-4-((5-phenylfuran-2-yl)methylene)-5-(trifluoromethyl)-2,4-dihydro-3H-pyrazol-3-one (I_{27})

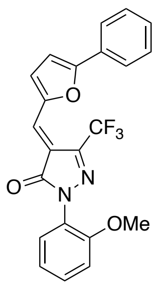

I_{27}

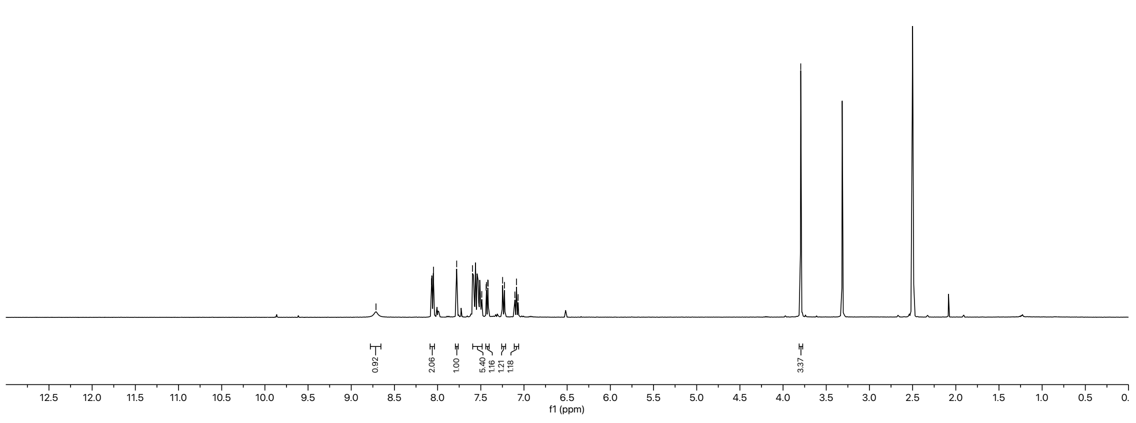

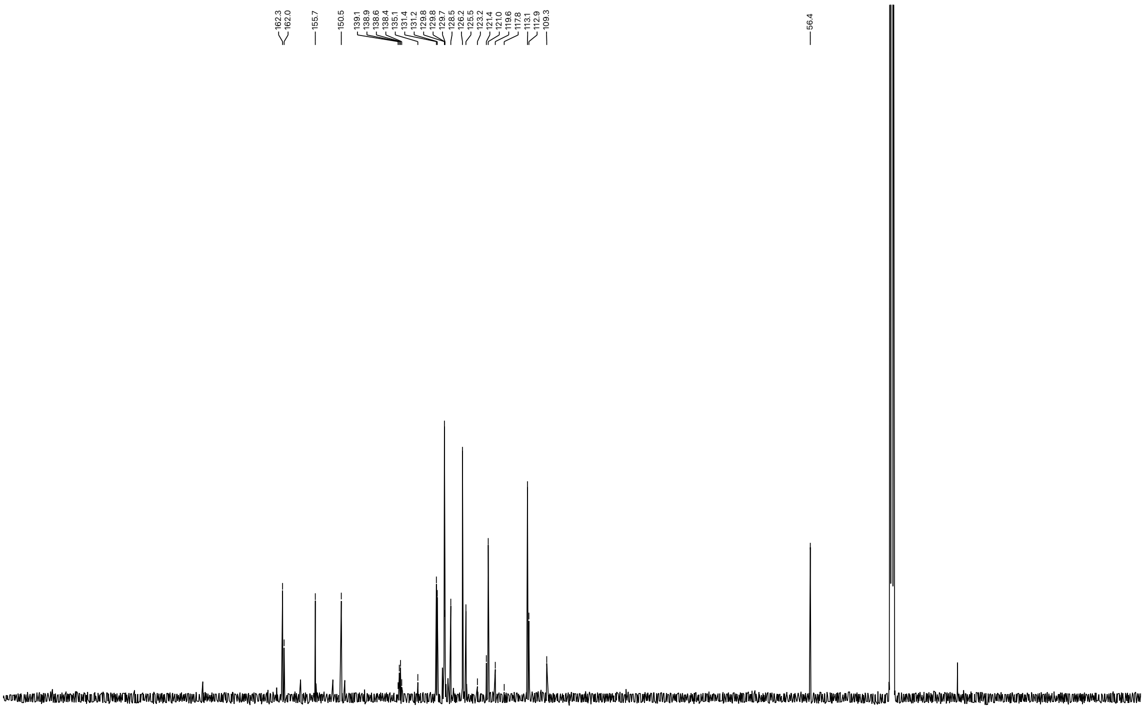

^{1}H-NMR and ^{13}C-NMR of (E)-2-(2-chlorophenyl)-4-((5-phenylfuran-2-yl)methylene)-5-(trifluoromethyl)-2,4-dihydro-3H-pyrazol-3-one (I_{28})

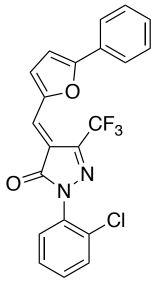

I_{28}

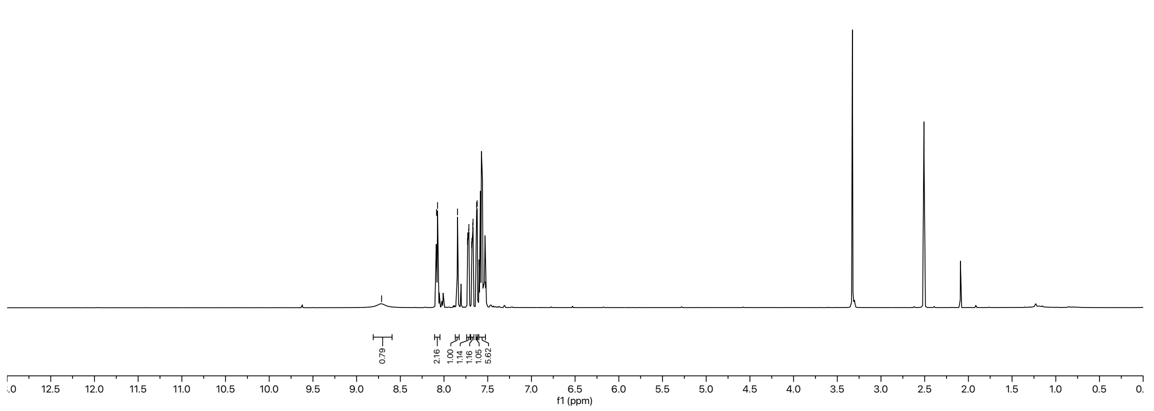

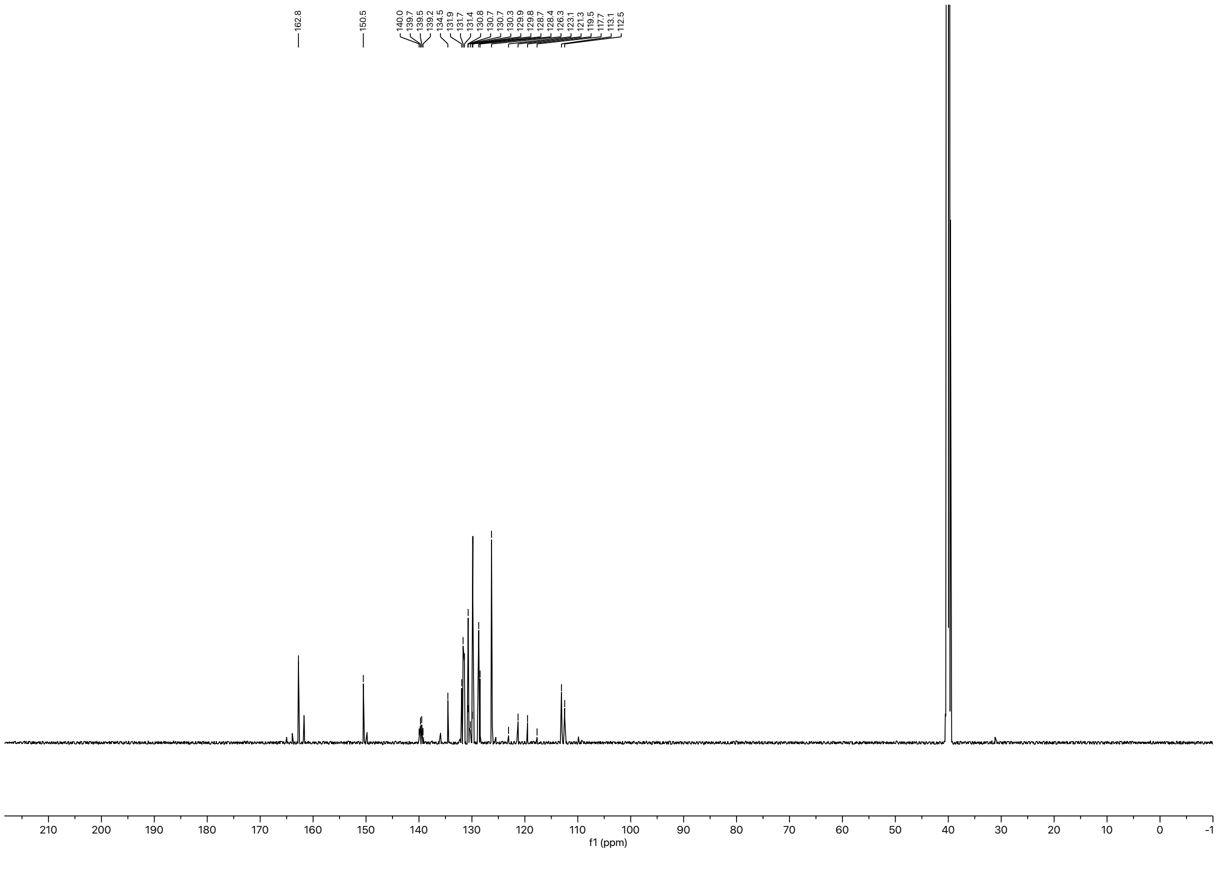

^{1}H-NMR and ^{13}C-NMR of (E)-2-(2-fluorophenyl)-4-((5-phenylfuran-2-yl)methylene)-5-(trifluoromethyl)-2,4-dihydro-3H-pyrazol-3-one (I_{29})

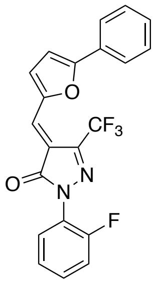

I_{29}

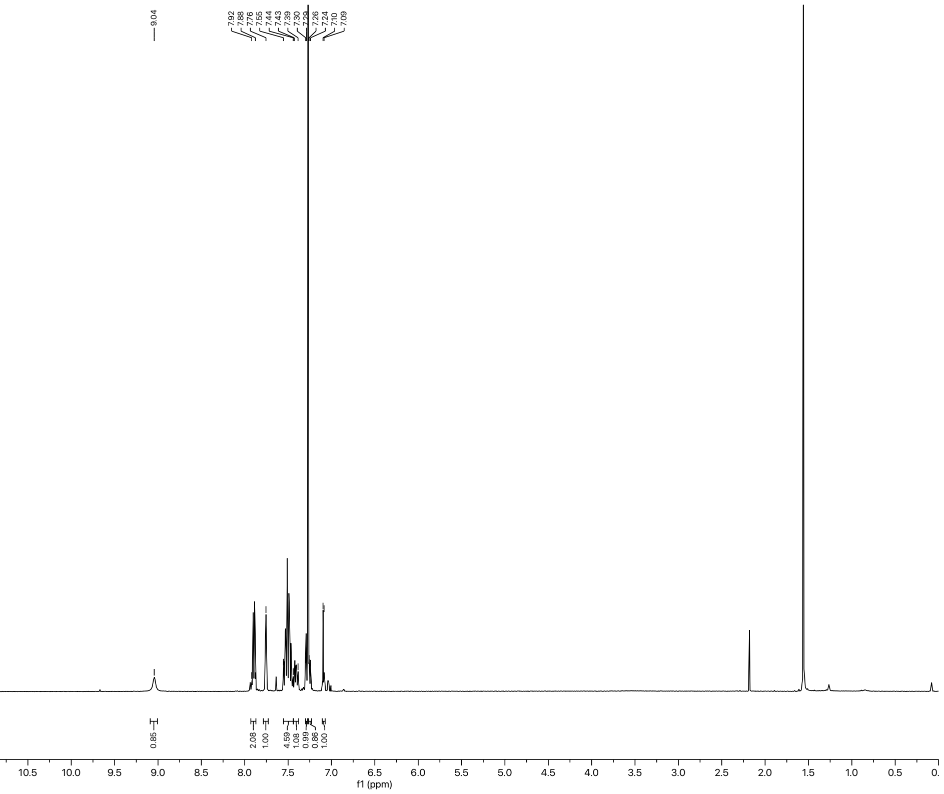

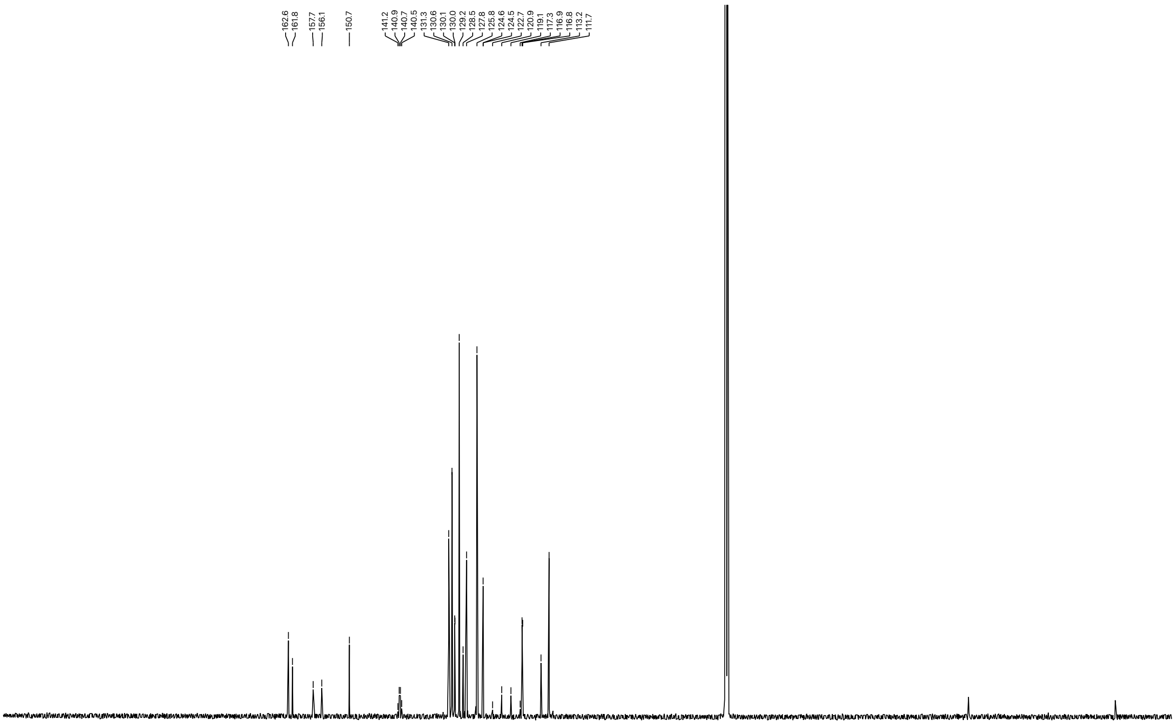

^{1}H-NMR and ^{13}C-NMR of (E)-2-(2,5-difluorophenyl)-4-((5-phenylfuran-2-yl)methylene)-5-(trifluoromethyl)-2,4-dihydro-3H-pyrazol-3-one (I_{30})

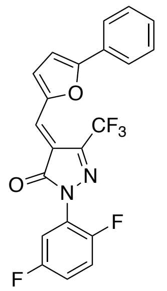

I_{30}

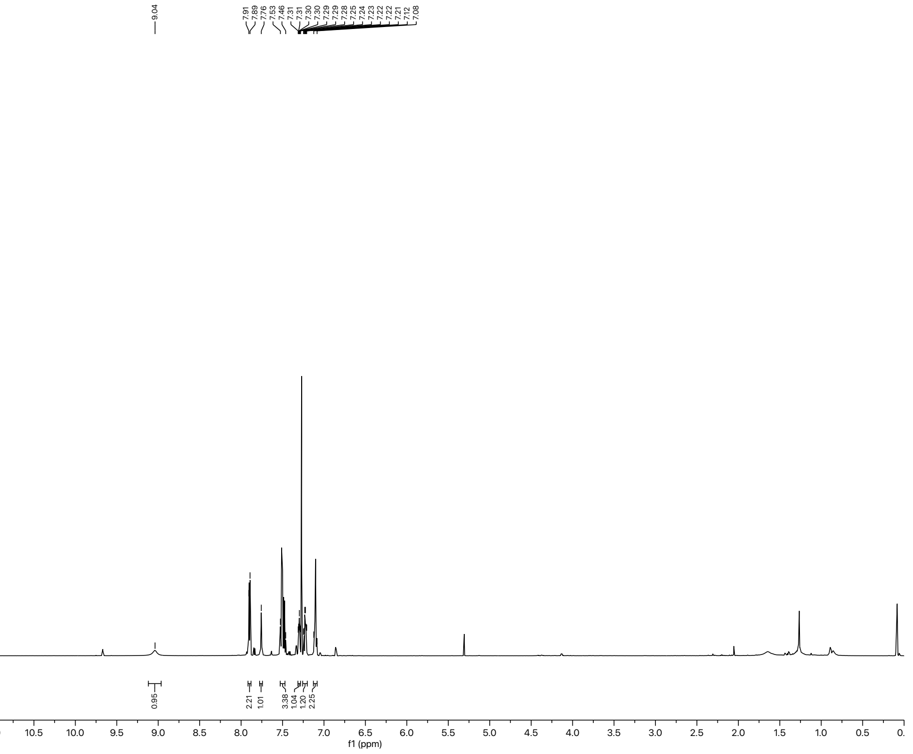

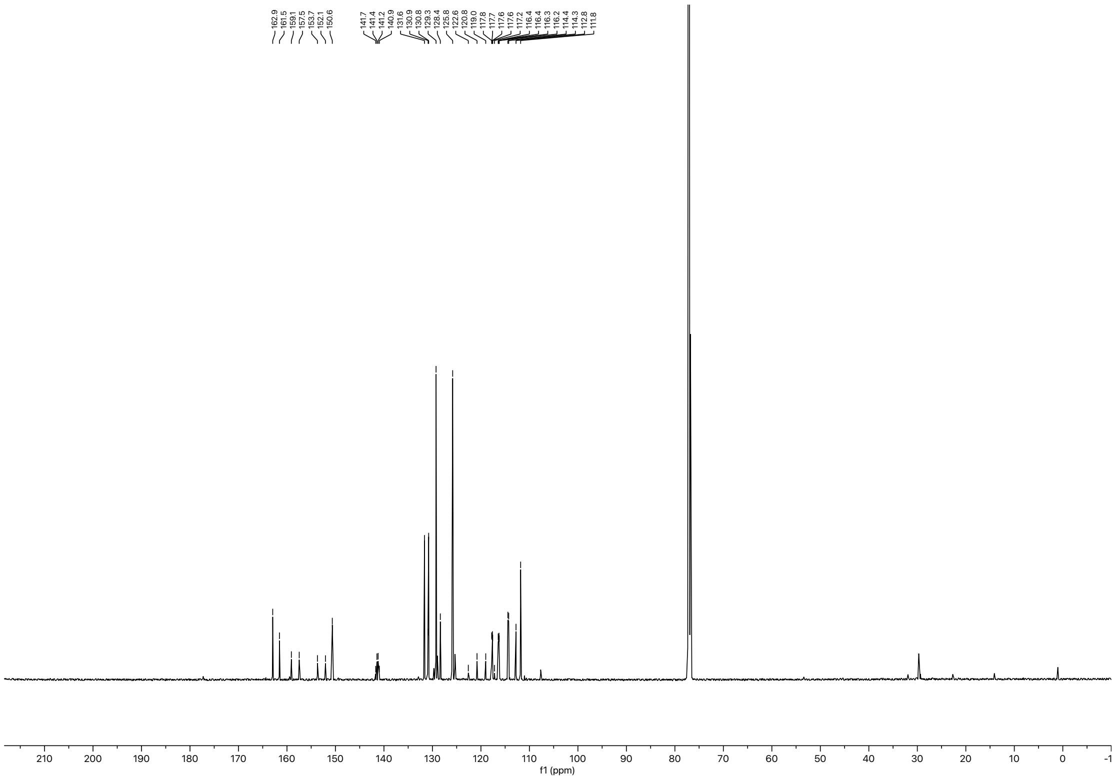

^{1}H-NMR and ^{13}C-NMR of (E)-3-(5-oxo-4-((5-phenylfuran-2-yl)methylene)-3-(trifluoromethyl)-4,5-dihydro-1H-pyrazol-1-yl)benzonitrile (I_{31})

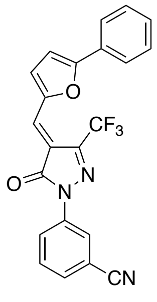

I_{31}

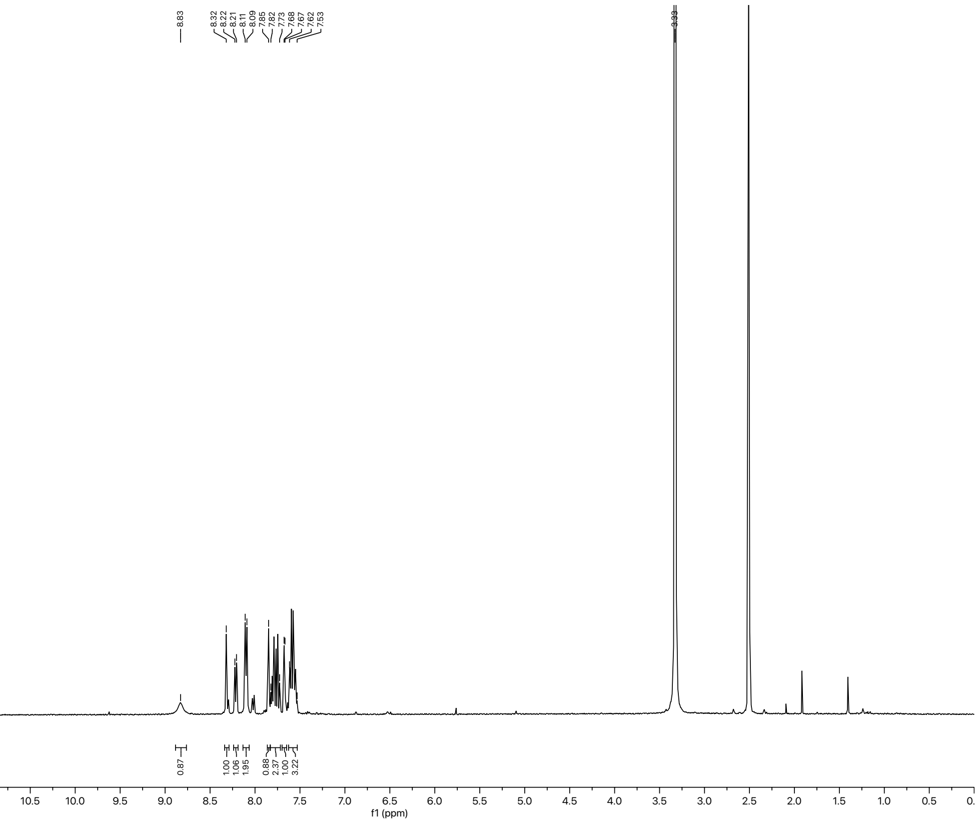

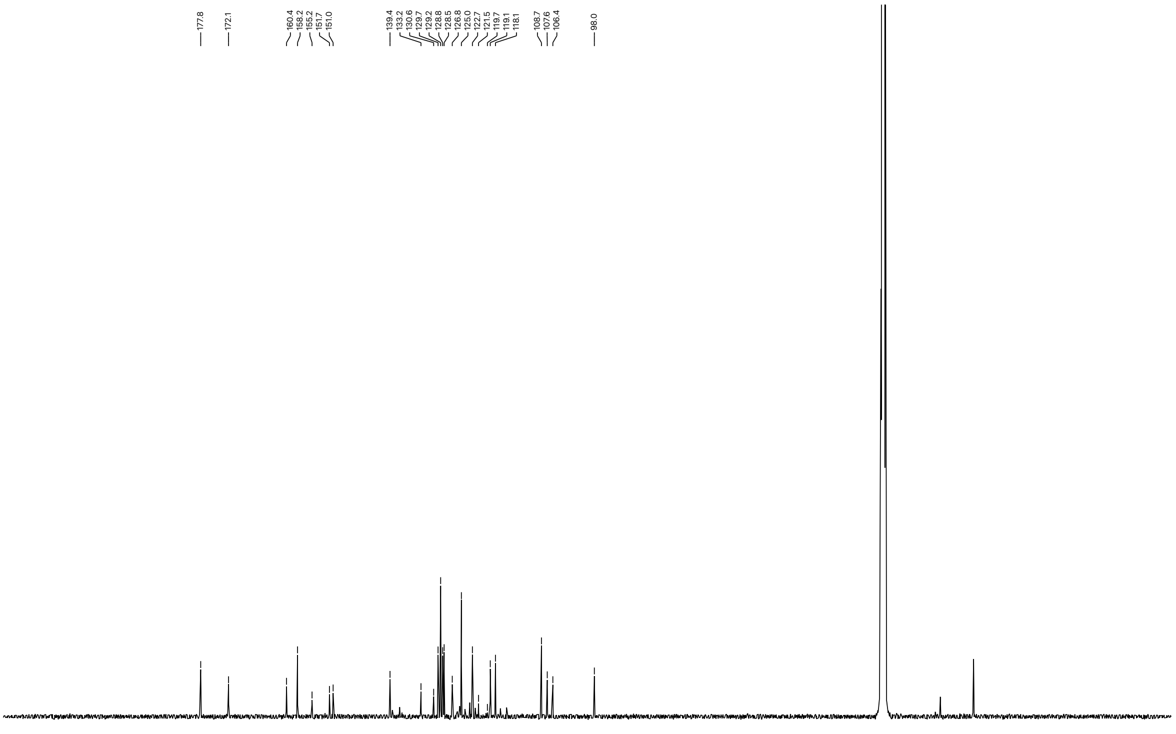

^{1}H-NMR and ^{13}C-NMR of (E)-4-((5-phenylfuran-2-yl)methylene)-2-(pyridin-3-yl)-5-(trifluoromethyl)-2,4-dihydro-3H-pyrazol-3-one (I_{32})

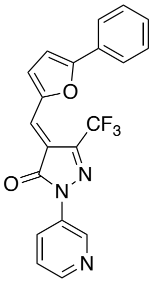

I_{32}

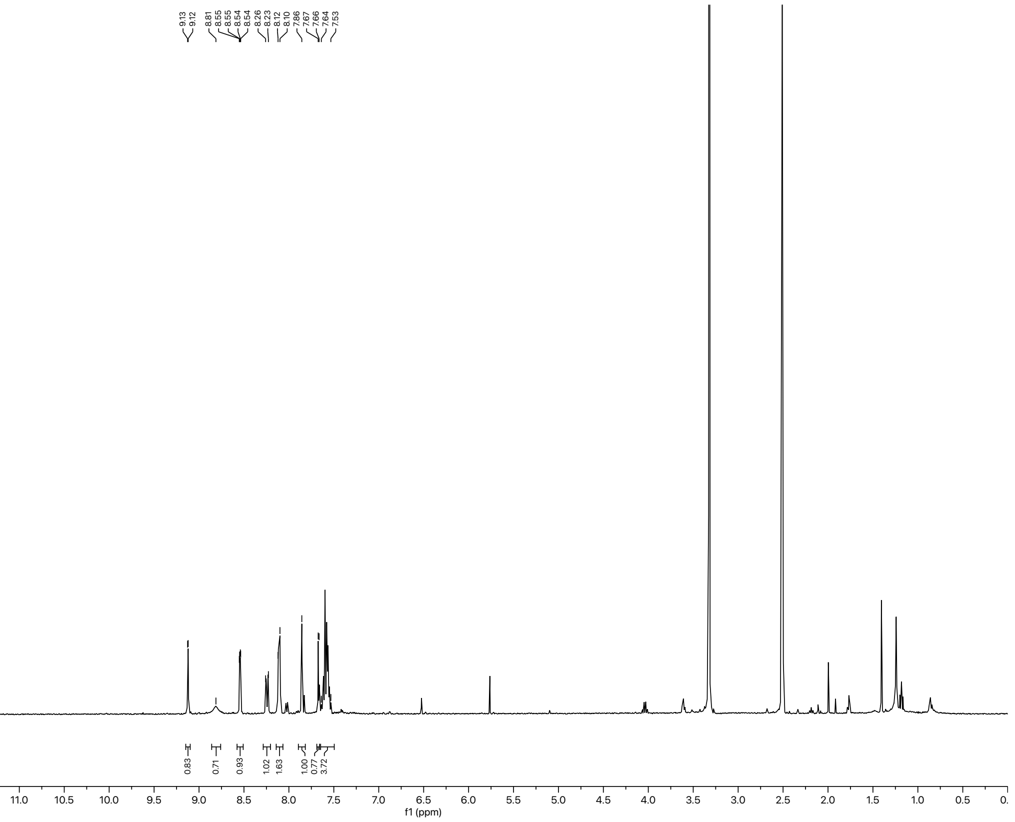

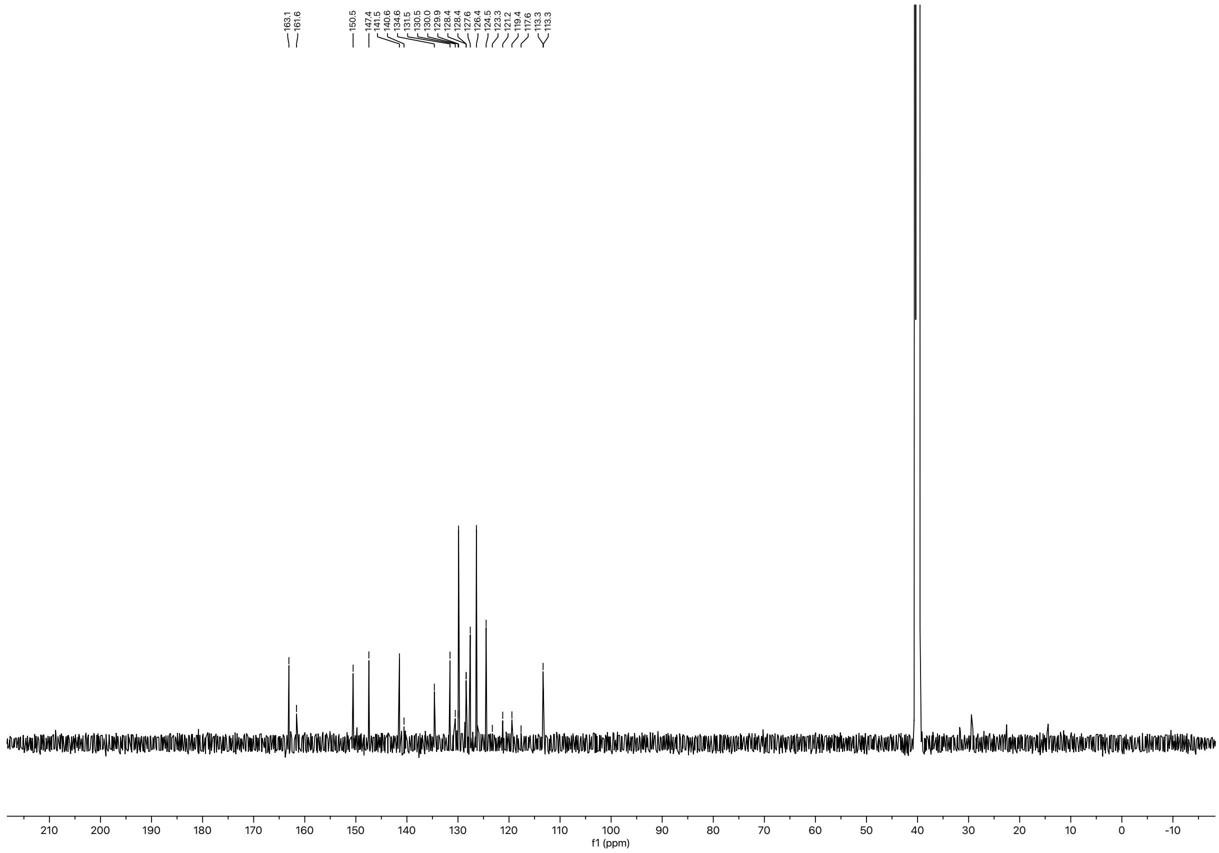

^{1}H-NMR and ^{13}C-NMR of (E)-3-(5-oxo-4-((5-phenylfuran-2-yl)methylene)-3-(trifluoromethyl)-4,5-dihydro-1H-pyrazol-1-yl)benzenesulfonamide (I_{33})

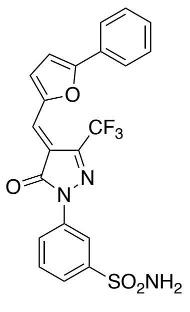

I_{33}

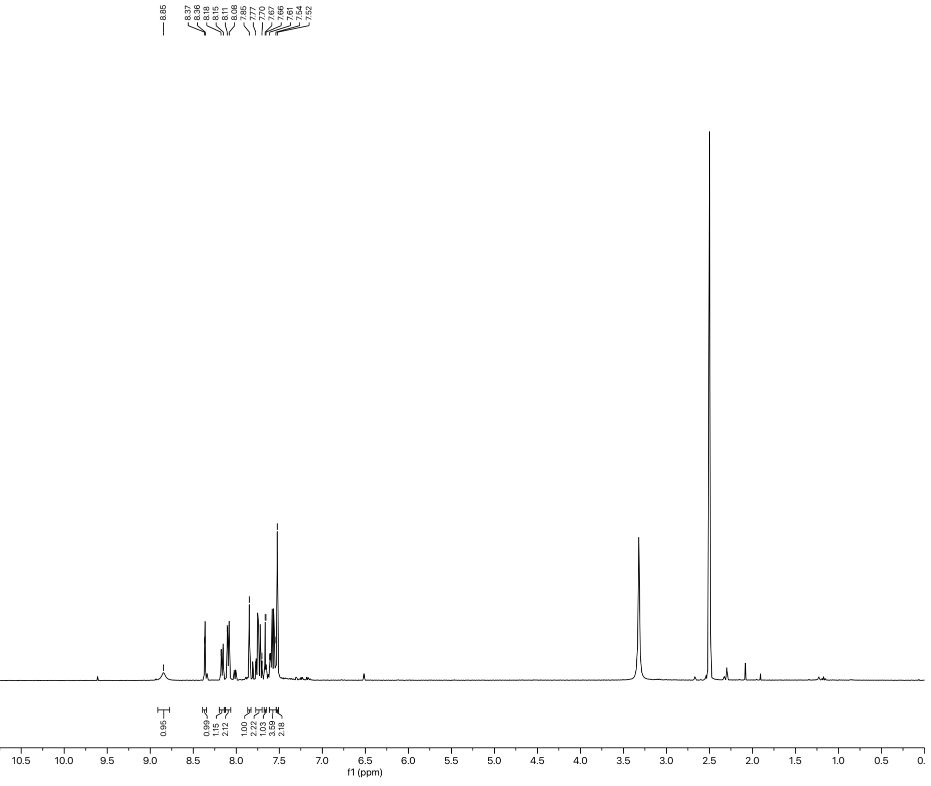

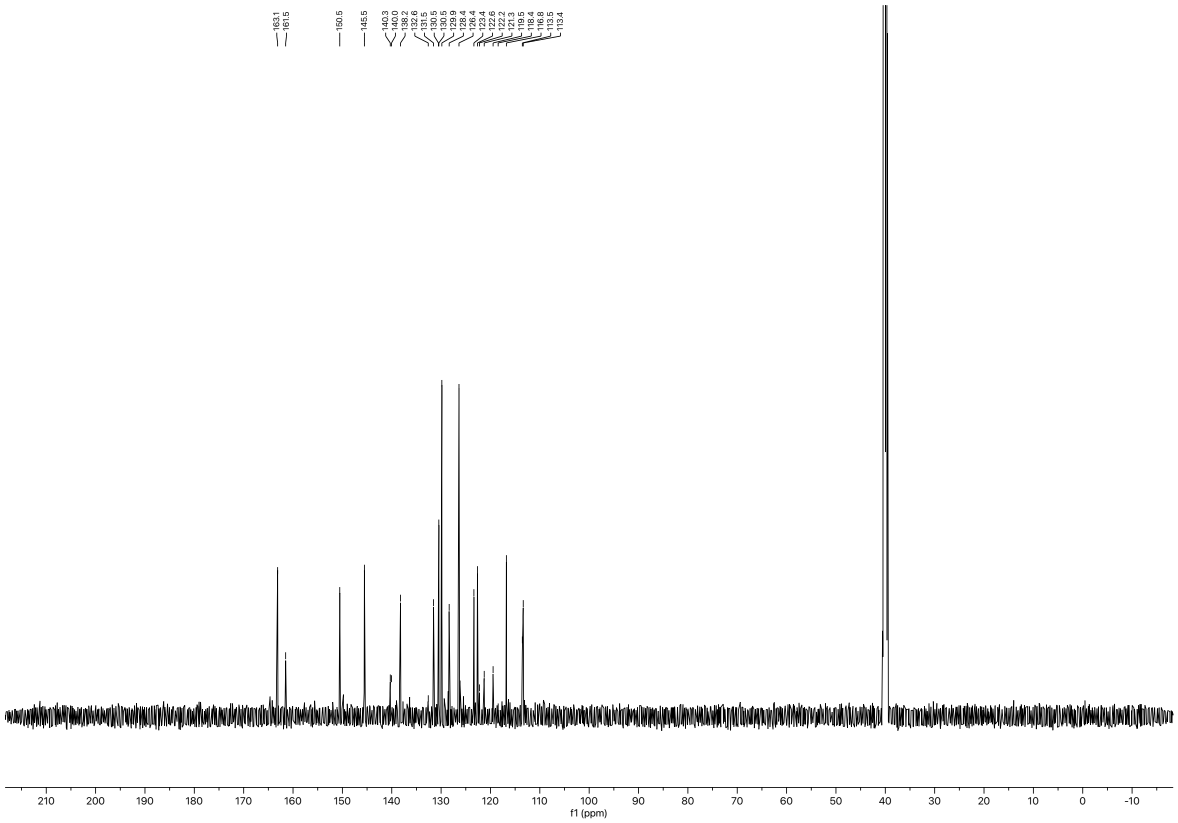

^{1}H-NMR and ^{13}C-NMR of (E)-3-(5-oxo-4-((5-phenylfuran-2-yl)methylene)-3-(trifluoromethyl)-4,5-dihydro-1H-pyrazol-1-yl)benzenesulfonic acid (I_{34})

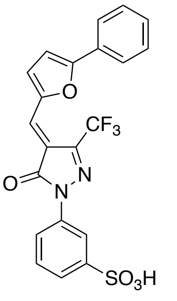

I_{34}

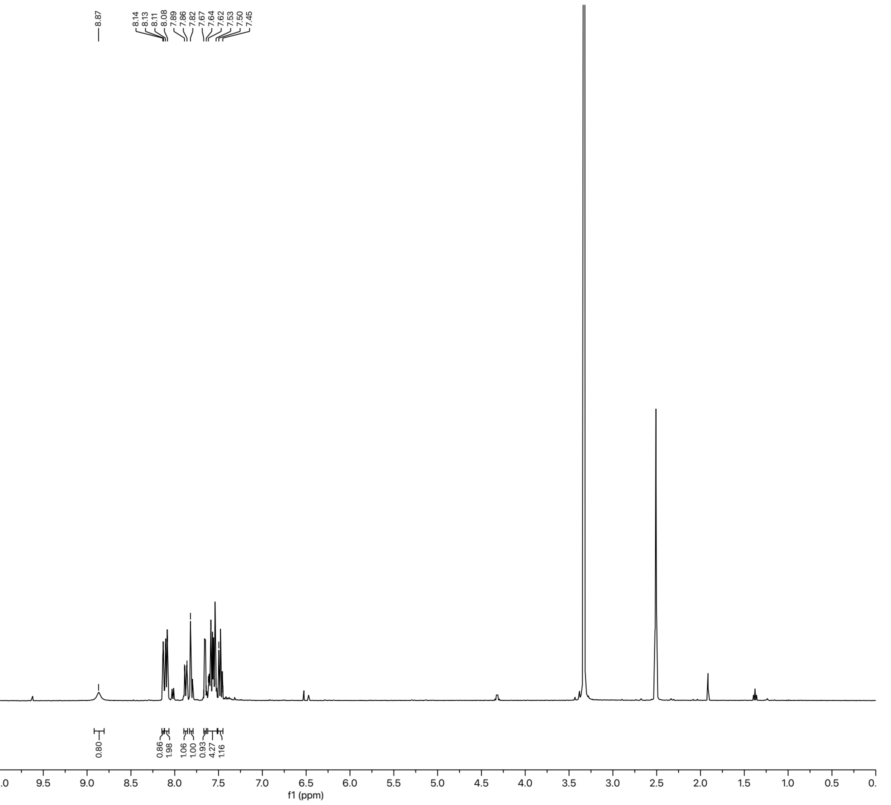

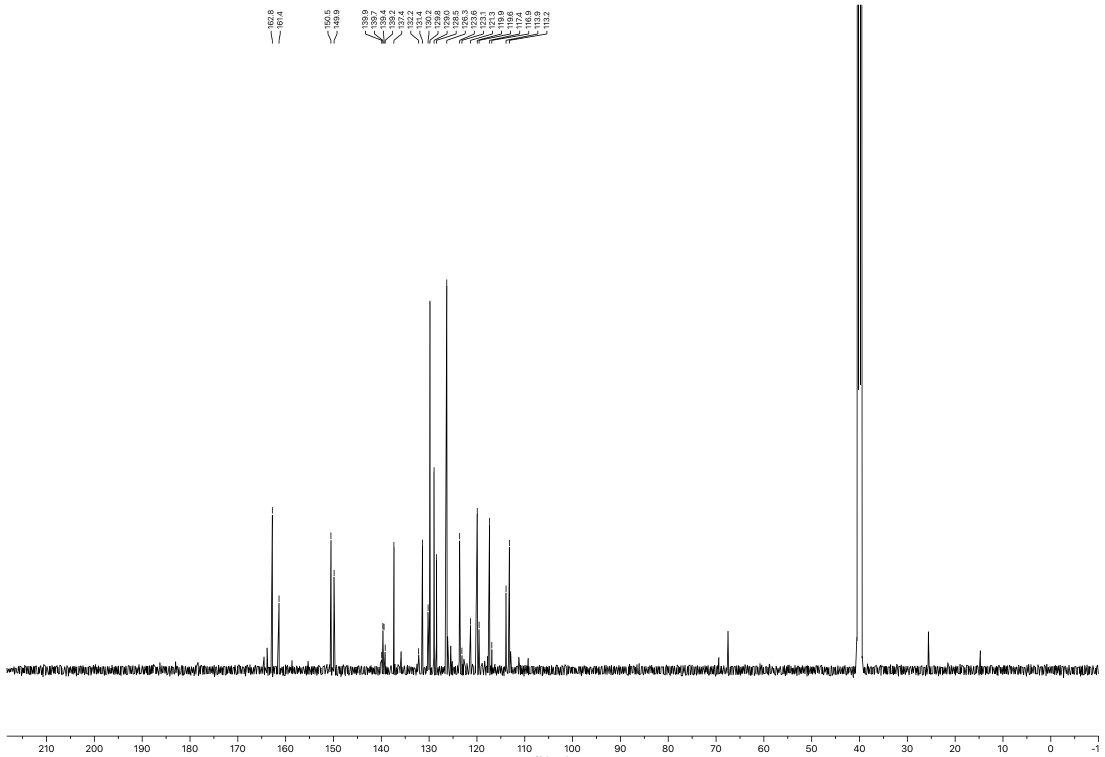

^{1}H-NMR and ^{13}C-NMR of (E)-2-chloro-5-(4-((5-(3-fluorophenyl)furan-2-yl)methylene)-5-oxo-3-(trifluoromethyl)-4,5-dihydro-1H-pyrazol-1-yl)benzoic acid (I_{36})

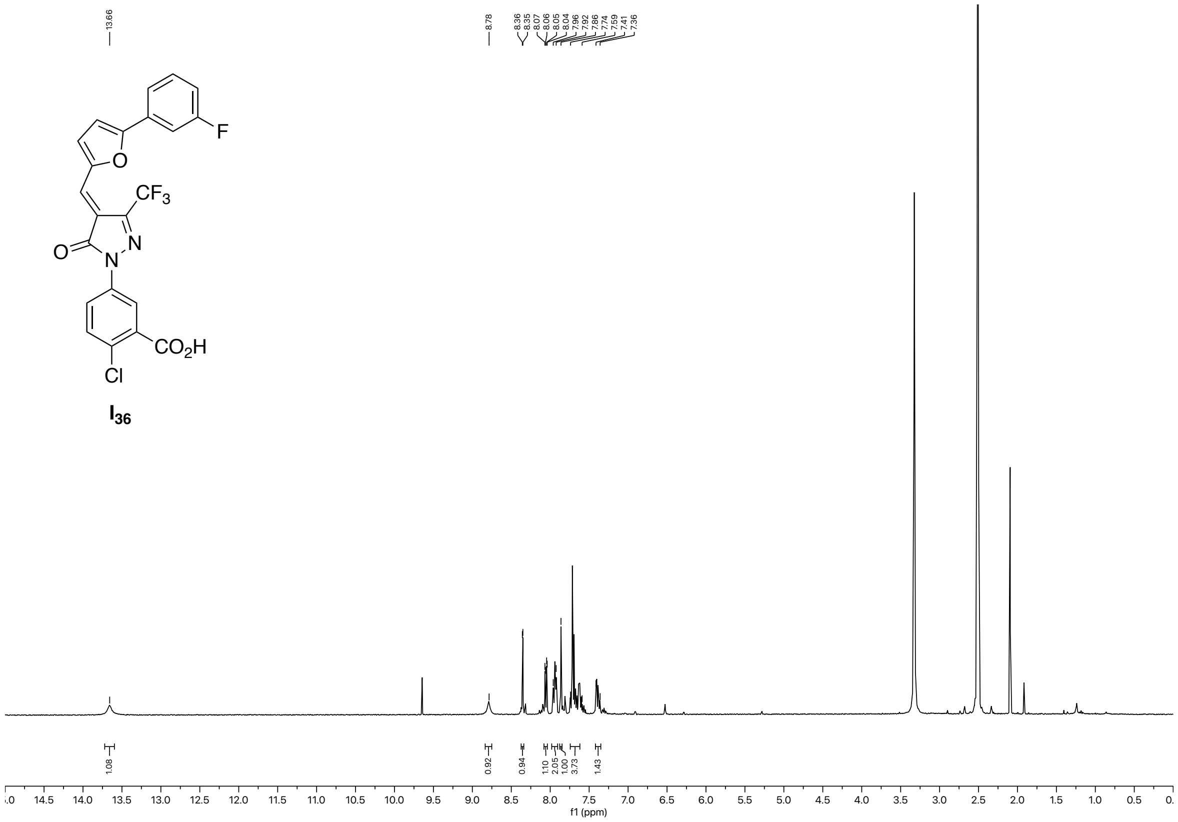

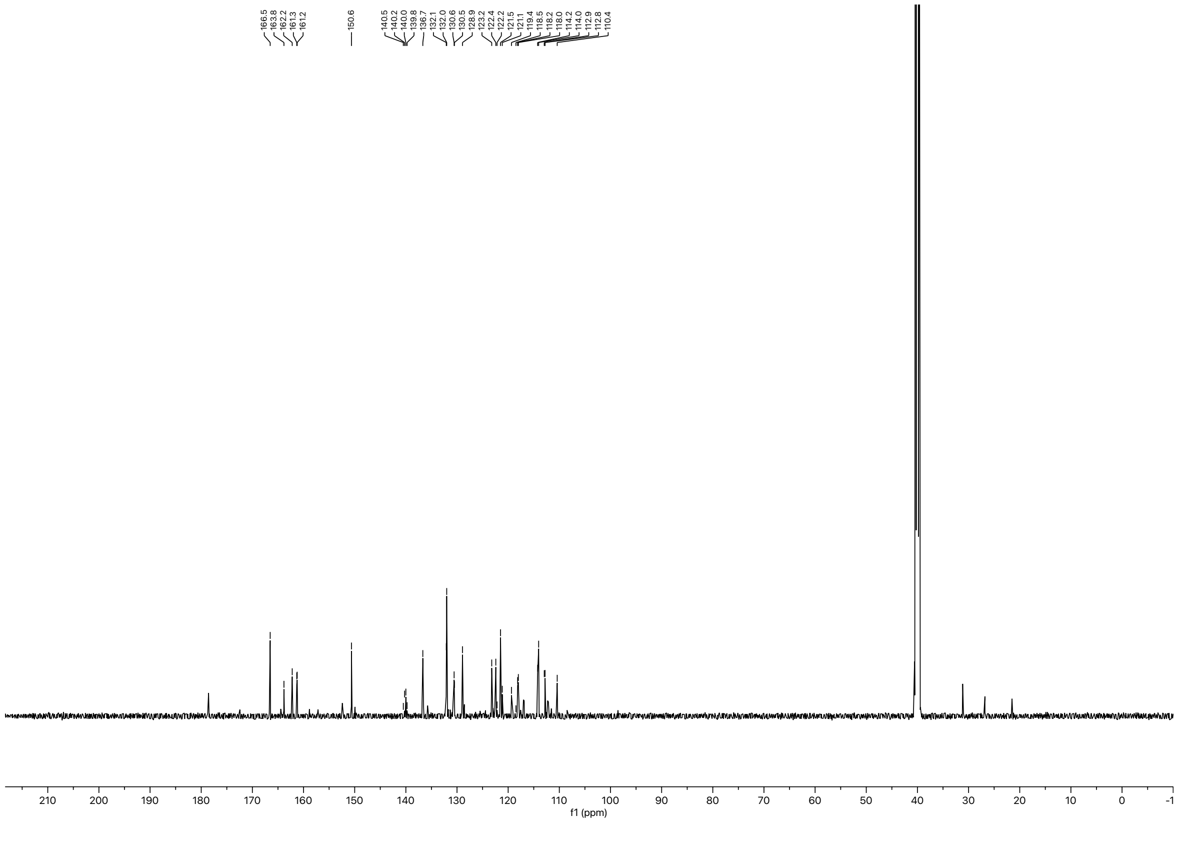

^{1}H-NMR and ^{13}C-NMR of (E)-2-chloro-5-(5-oxo-3-(trifluoromethyl)-4-((5-(3-(trifluoromethyl)phenyl)furan-2-yl)methylene)-4,5-dihydro-1H-pyrazol-1-yl)benzoic acid (I_{37})

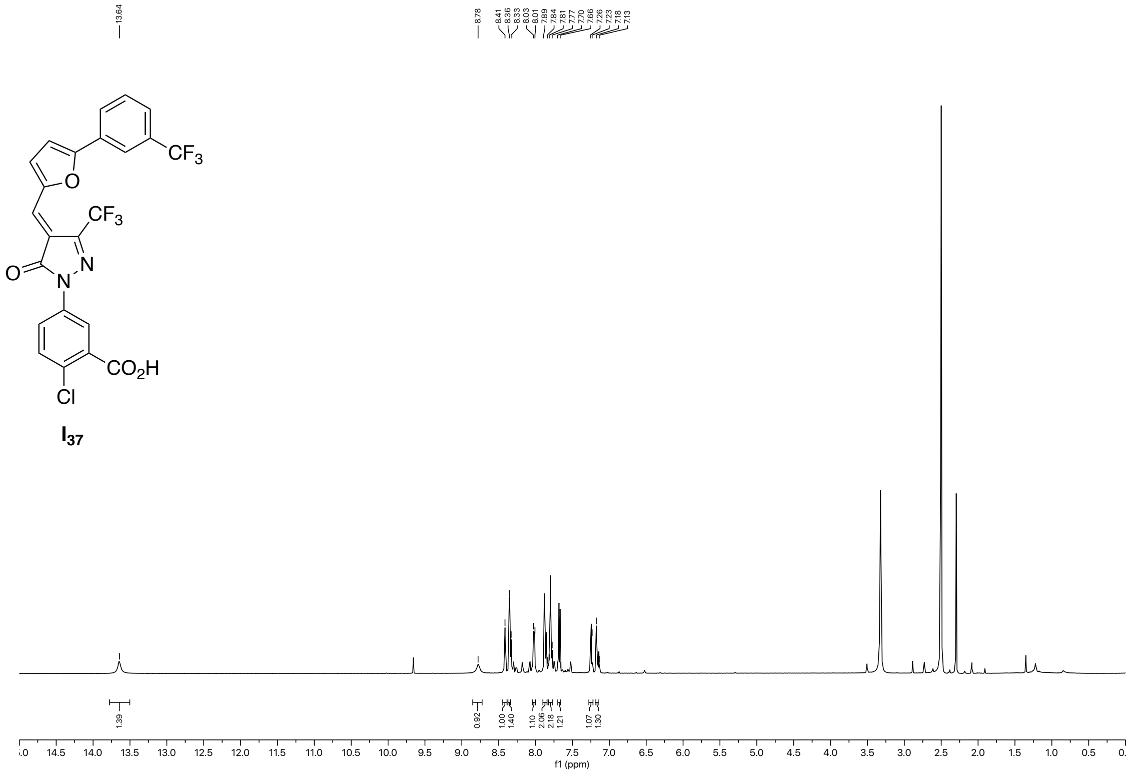

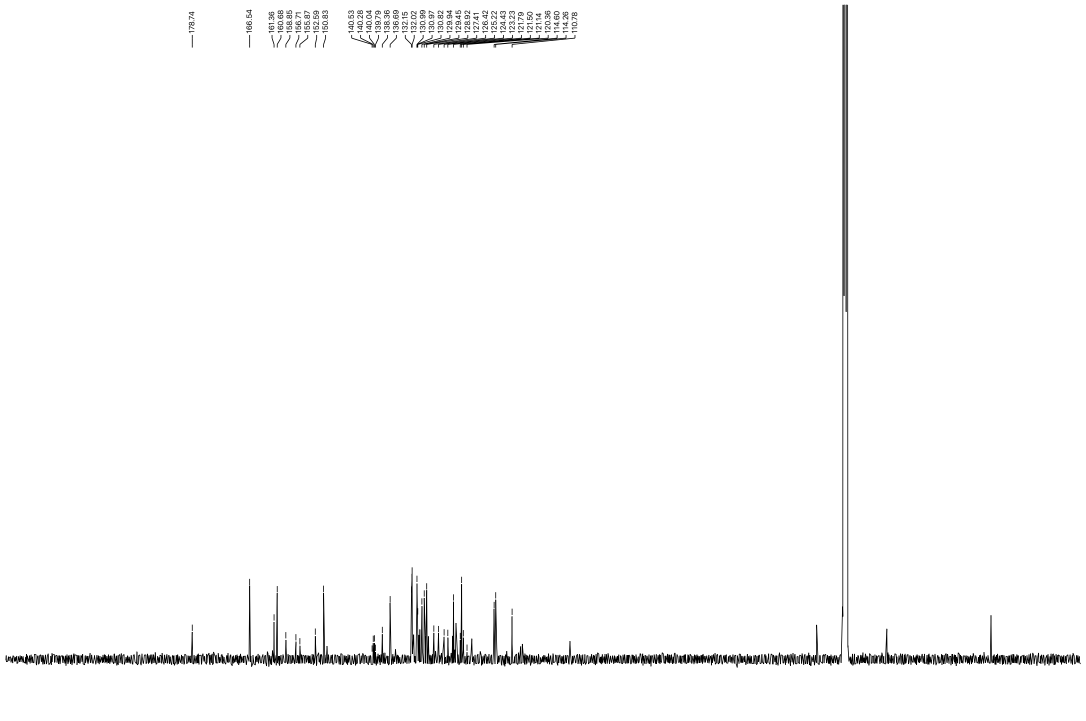

^{1}H-NMR and ^{13}C-NMR of (E)-2-chloro-5-(4-((5-(4-fluorophenyl)furan-2-yl)methylene)-5-oxo-3-(trifluoromethyl)-4,5-dihydro-1H-pyrazol-1-yl)benzoic acid (I_{38})

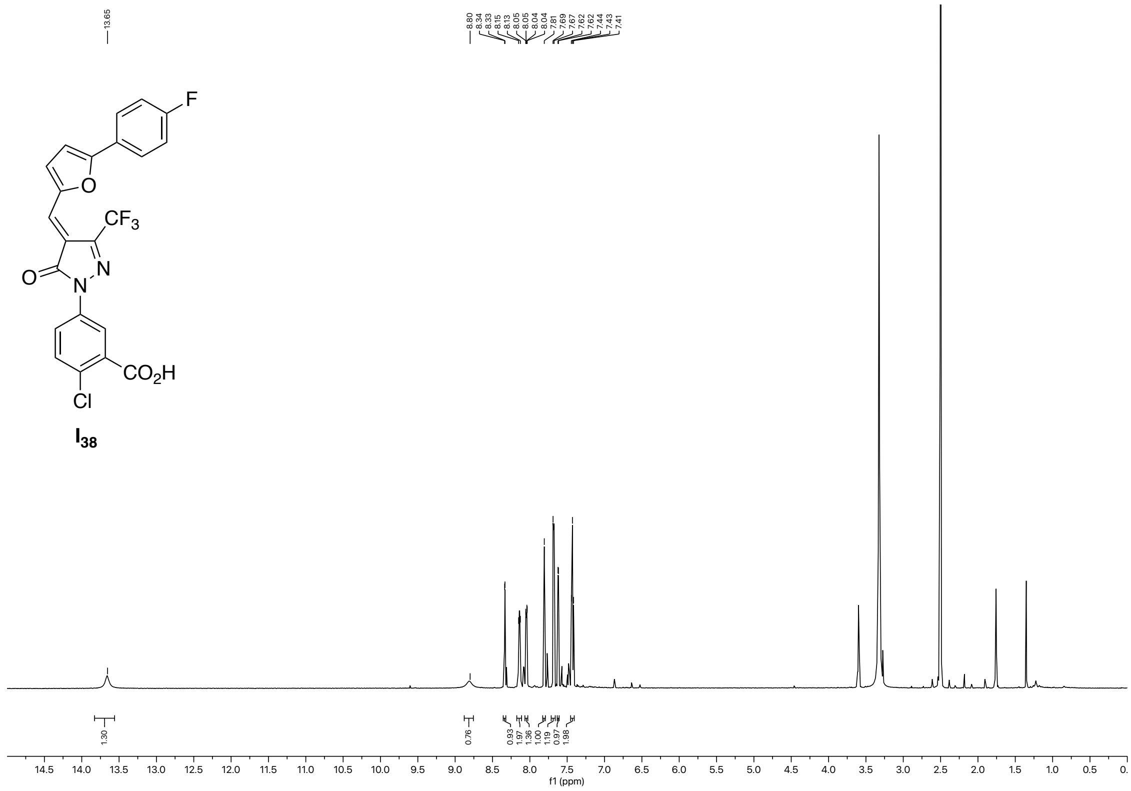

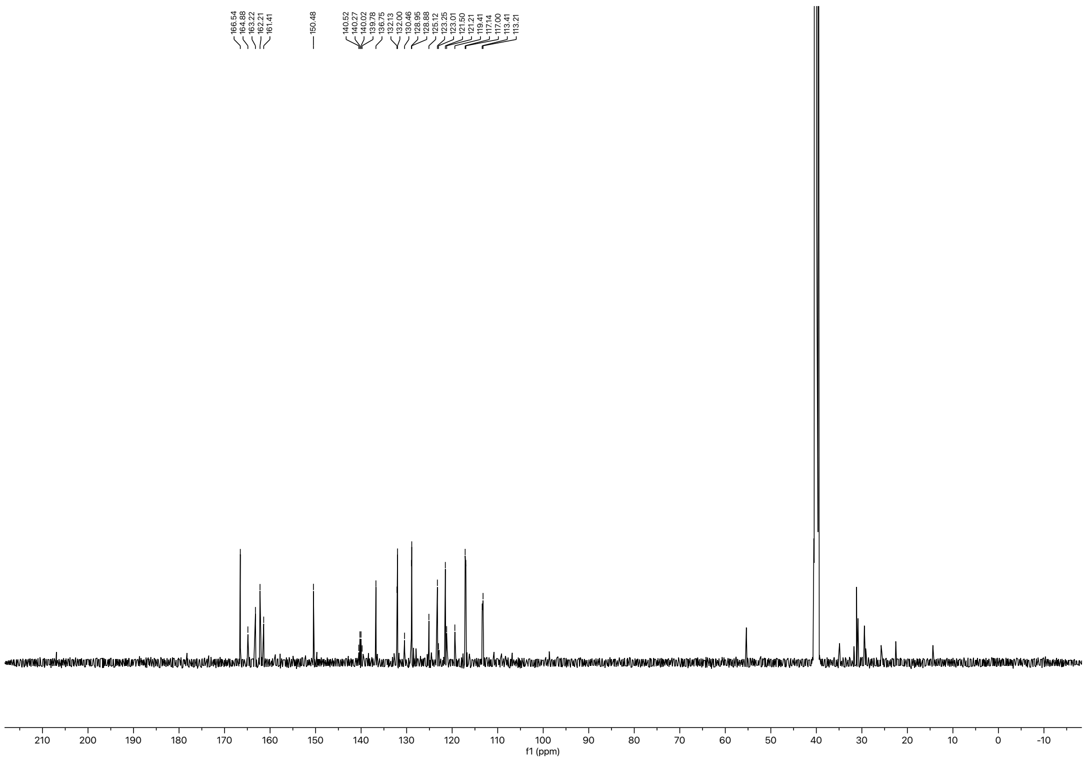

^{1}H-NMR and ^{13}C-NMR of (E)-2-chloro-5-(4-((5-(3-methoxyphenyl)furan-2-yl)methylene)-5-oxo-3 (trifluoromethyl)-4,5-dihydro-1H-pyrazol-1-yl)benzoic acid (I$_{39}$)

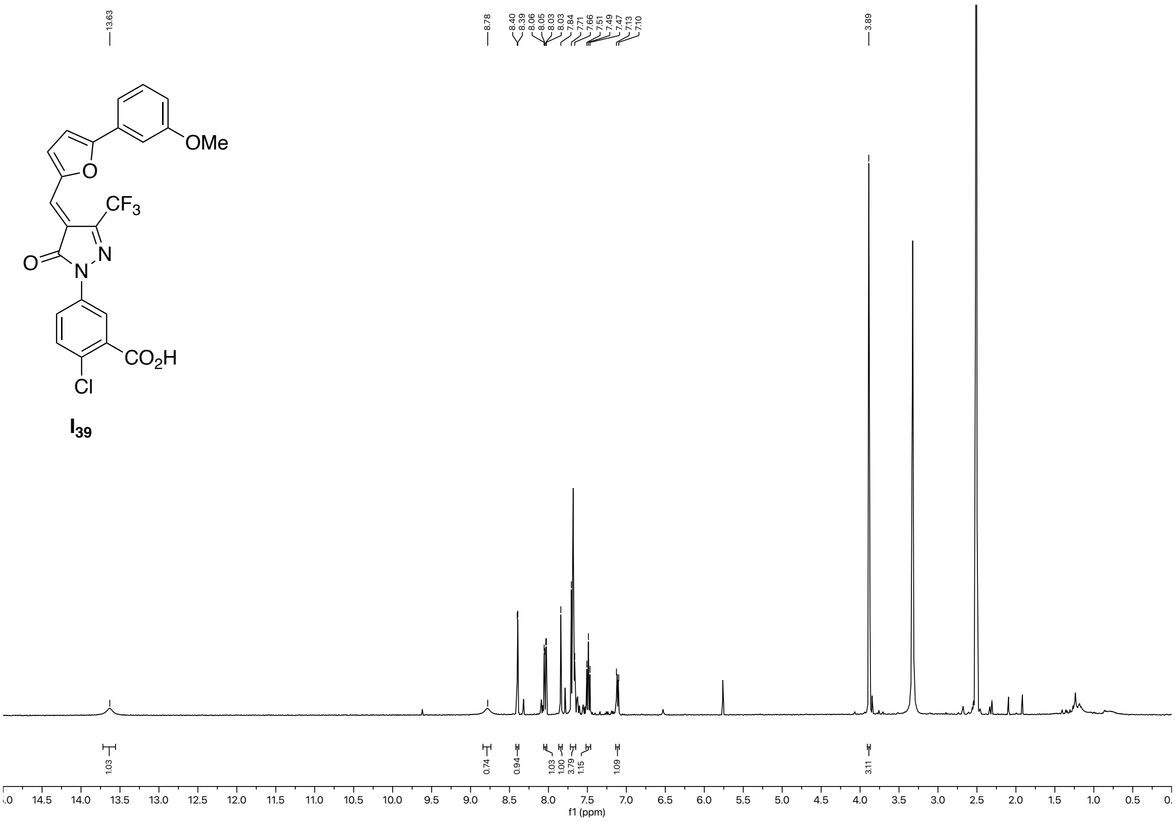

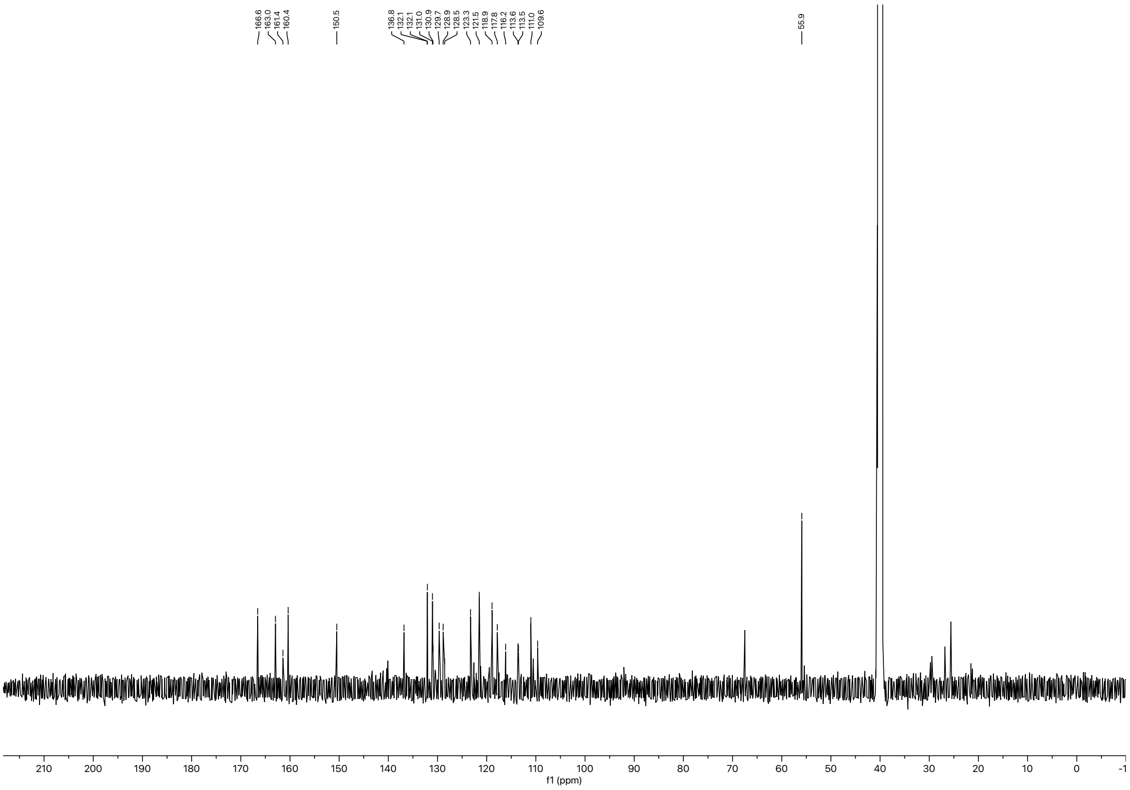

^{1}H-NMR and ^{13}C-NMR of (E)-2-chloro-5-(5-oxo-4-((5-phenylthiophen-2-yl)methylene)-3-(trifluoromethyl)-4,5-dihydro-1H-pyrazol-1-yl)benzoic acid (I_{40})

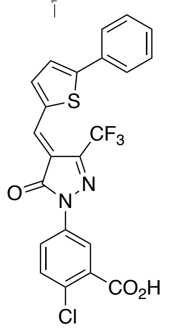

I_{40}

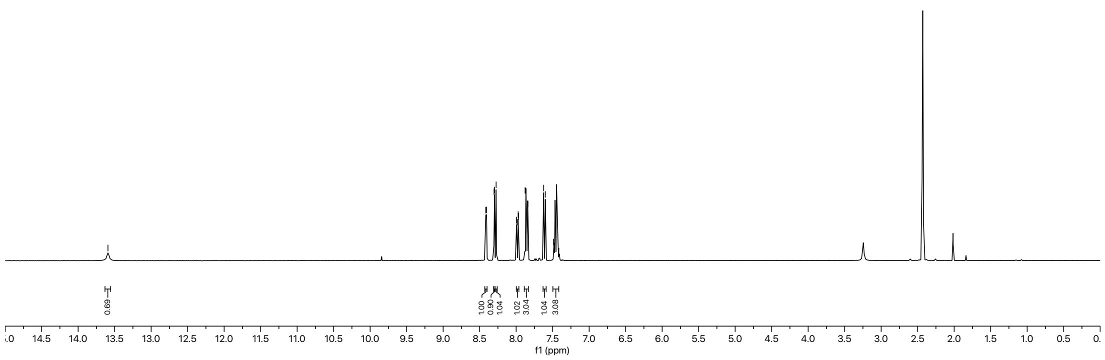

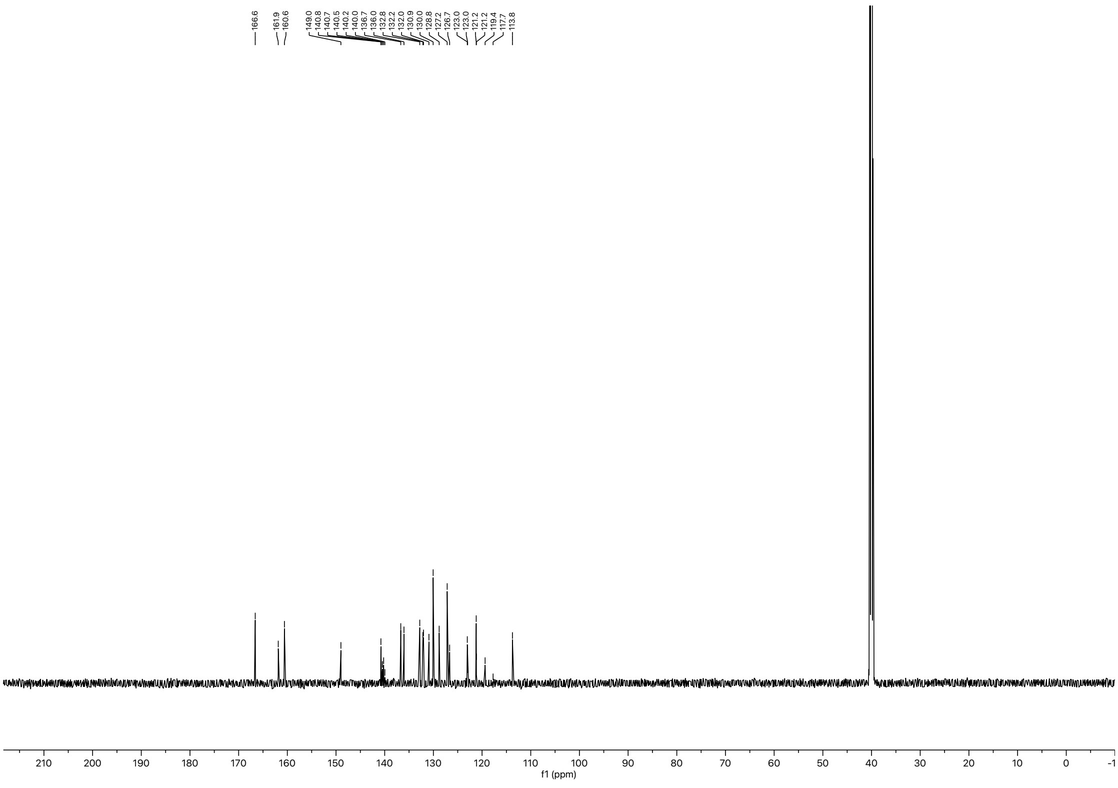

^{1}H-NMR and ^{13}C-NMR of acetic acid, (E)-5-(3-(5-oxo-4-((5-phenylfuran-2-yl)methylene)-3-(trifluoromethyl)-4,5-dihydro-1H-pyrazol-1-yl)phenyl)-1H-tetrazol-1-ium salt (I_{35})

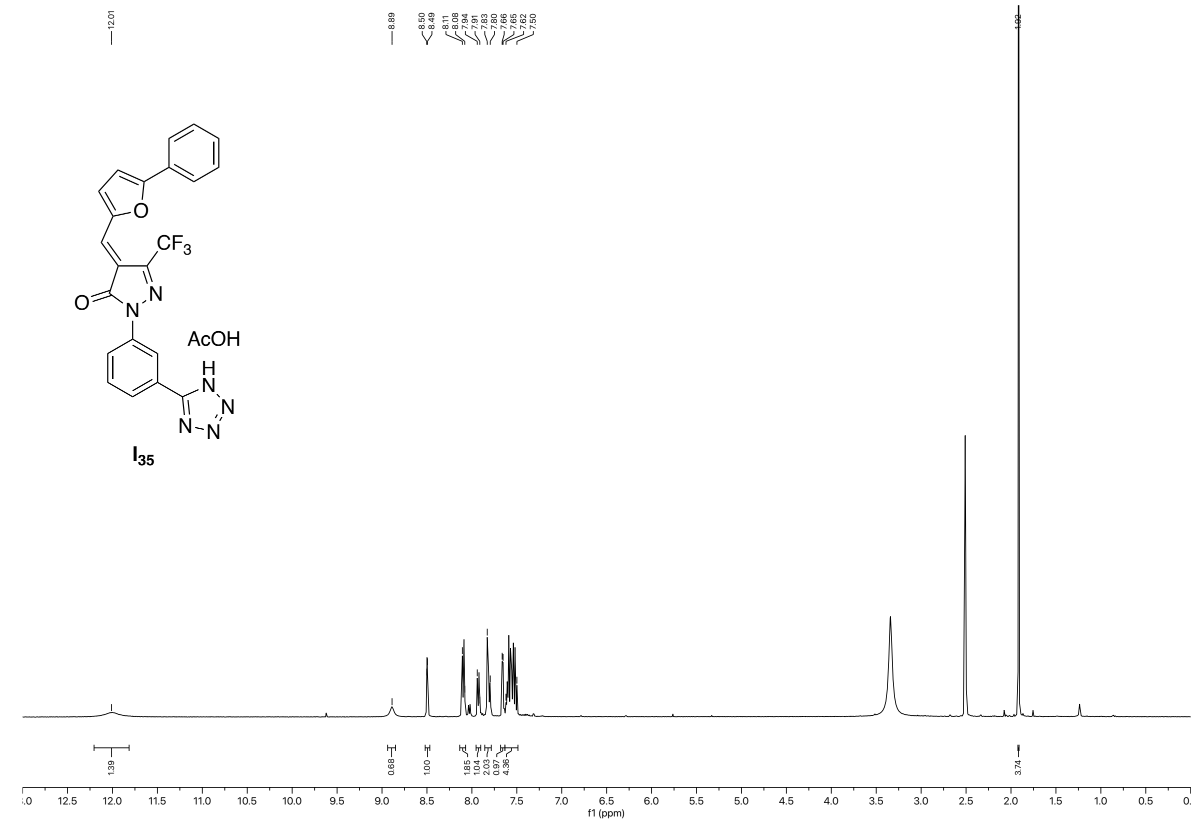

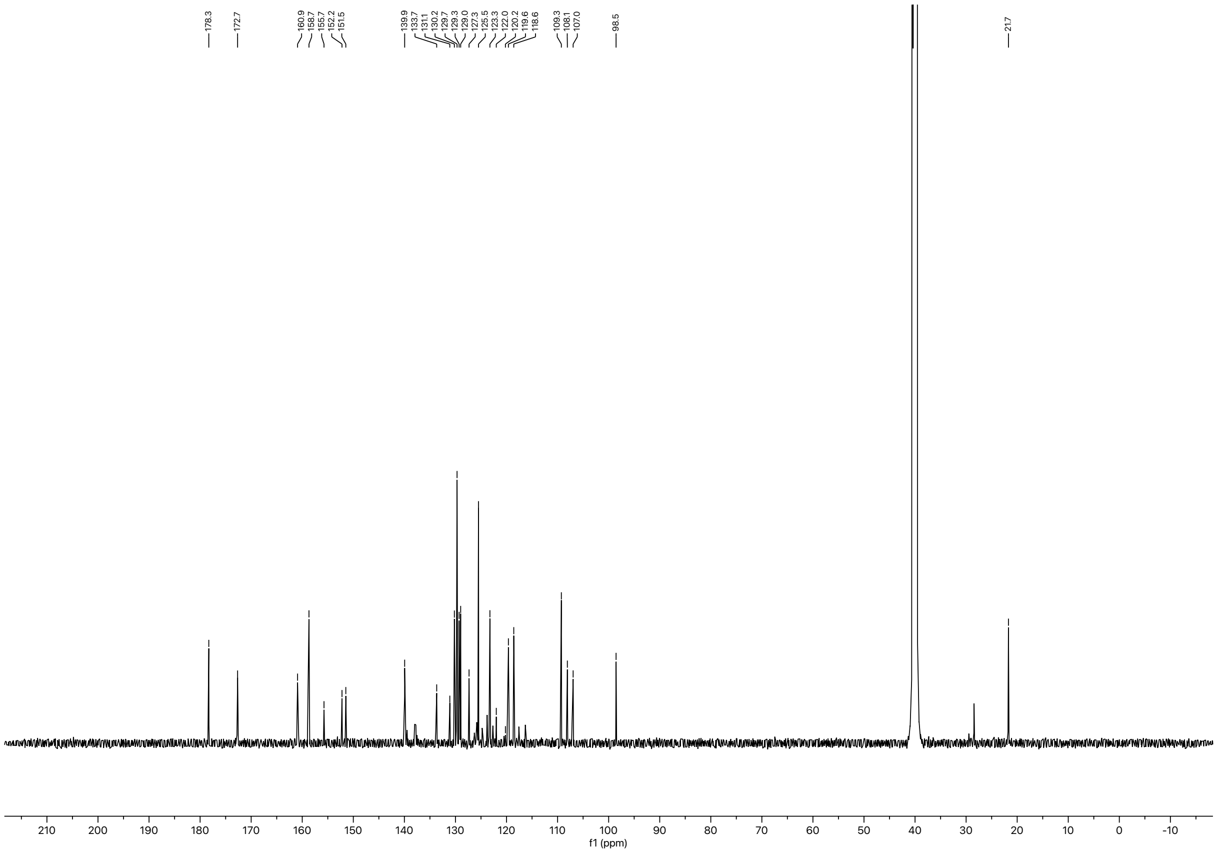

^{1}H-NMR of (S)-5-amino-5-carboxypentan-1-aminium (E)-2-chloro-5-(5-oxo-4-((5-phenylfuran-2-yl)methylene)-3-(trifluoromethyl)-4,5-dihydro-1H-pyrazol-1-yl)benzoate (EN460-LYS)

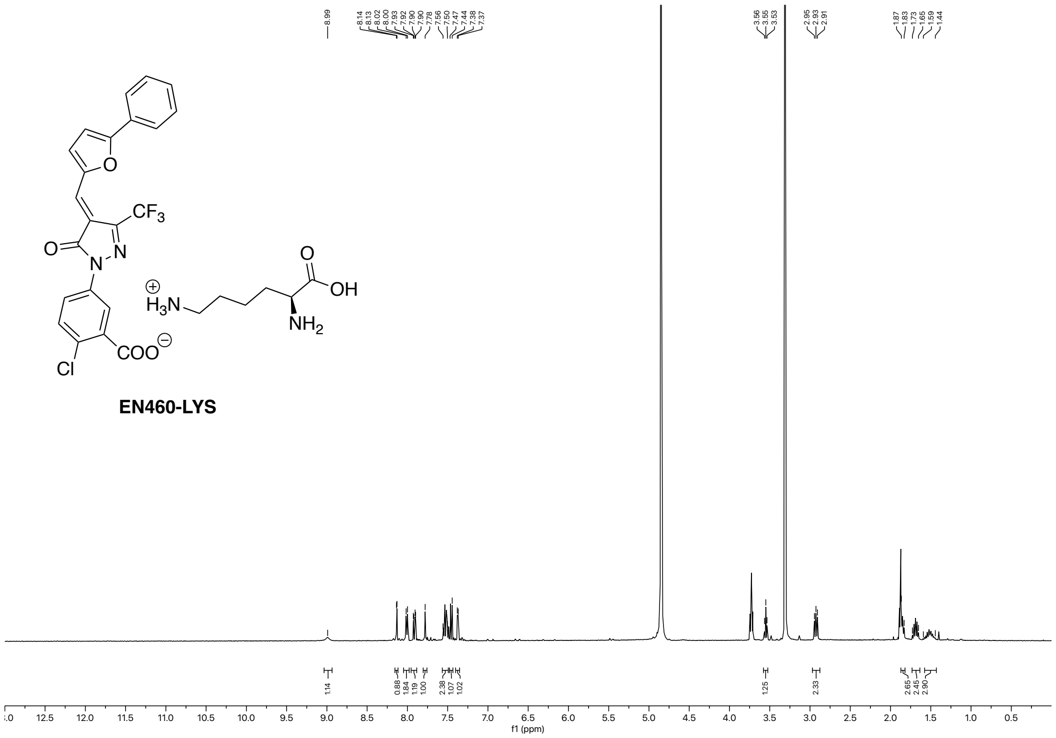

^{1}H-NMR of (S)-5-amino-5-carboxypentan-1-aminium (E)-2-chloro-5-(5-oxo-4-((5-phenylfuran-2-yl)methylene)-3-(trifluoromethyl)-4,5-dihydro-1H-pyrazol-1-yl)benzoate (EN460-ARG)

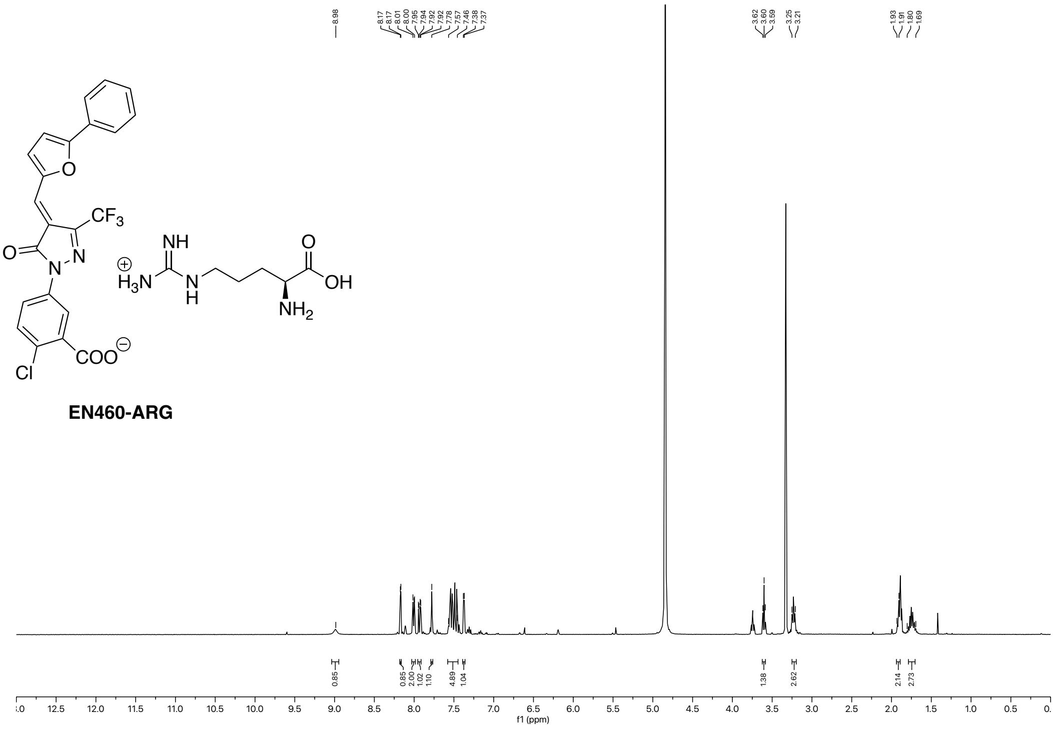

^{1}H-NMR of bis(2-hydroxyethyl)ammonium (E)-2-chloro-5-(5-oxo-4-((5-phenylfuran-2-yl)methylene)-3-(trifluoromethyl)-4,5-dihydro-1H-pyrazol-1-yl)benzoate (EN460-DEA)

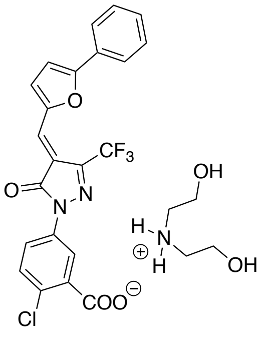

EN460-DEA

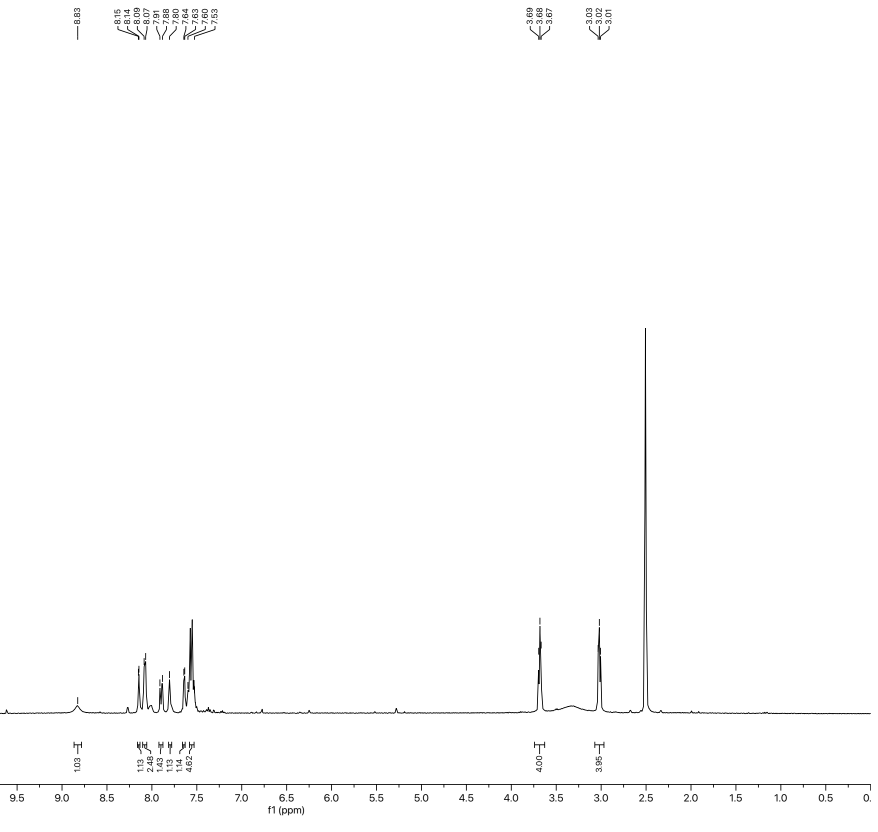

Figure Supplementary 1 linked to Figure 3

A) Time course of AUR fluorescence (RFU) in reactions containing ERO1, PDIA1, EN460 and the compounds (I24-I40) dissolved in DMSO. B) As above, time course of AUR fluorescence (RFU) of EN460 and I34 dissolved in in either DMSO or aqueous solution (physiological solution). C) Inhibition plots of EN460 and all the compounds (I24-I40): The percentage of ERO1 inhibition is plotted against the logarithm of compound concentration.

Figure Supplementary 2 linked to Figure 3

A) Non-reducing Immunoblot of endogenous ERO1 in lysates of vehicle-treated MDAMB231 cells or exposed to DTT, EN460 and the different salts (Arg, Lys and Diet) at 20 μM and ERO1 Knock out (KO) MDAMB231 cells. ERO1 red. stands for ERO1 reduced, ERO1 ox. for oxidized. B) Non-reducing and reducing immunoblots of endogenous ERO1 in lysates of vehicle-treated WT and ERO1 KO MDAMB231 or exposed to DTT or the indicated different doses of EN460 and its Arginine salt.

Figure Supplementary 3 linked to Figure 7

Traces of $\Delta F/F_0$ TMRM fluorescence in WT and SEPN1 KO myotubes treated with EN460 or I29, followed by sequential addition of oligomycin and FCCP.

Figure Supplementary 1 linked to Figure 3

A) Time course of AUR fluorescence (RFU) in reactions containing ERO1, PDIA1, EN460 and the compounds (I24-I40) dissolved in DMSO. B) As above, time course of AUR fluorescence (RFU) of EN460 and I34 dissolved in in either DMSO or aqueous solution (physiological solution). C) Inhibition plots of EN460 and all the compounds (I24-I40): The percentage of ERO1 inhibition is plotted against the logarithm of compound concentration.

Figure Supplementary 2 linked to Figure 3

A) Non-reducing Immunoblot of endogenous ERO1 in lysates of vehicle-treated MDAMB231 cells or exposed to DTT, EN460 and the different salts (Arg, Lys and Diet) at 20 µM and ERO1 Knock out (KO) MDAMB231 cells. ERO1 red. stands for ERO1 reduced, ERO1 ox. for oxidized. B) Non-reducing and reducing immunoblots of endogenous ERO1 in lysates of vehicle-treated WT and ERO1 KO MDAMB231 or exposed to DTT or the indicated different doses of EN460 and its Arginine salt.

Figure Supplementary 3 linked to Figure 7

Traces of $\Delta F/F_0$ TMRM fluorescence in WT and SEPN1 KO myotubes treated with EN460 or I29, followed by sequential addition of oligomycin and FCCP.

Figure Sup.1

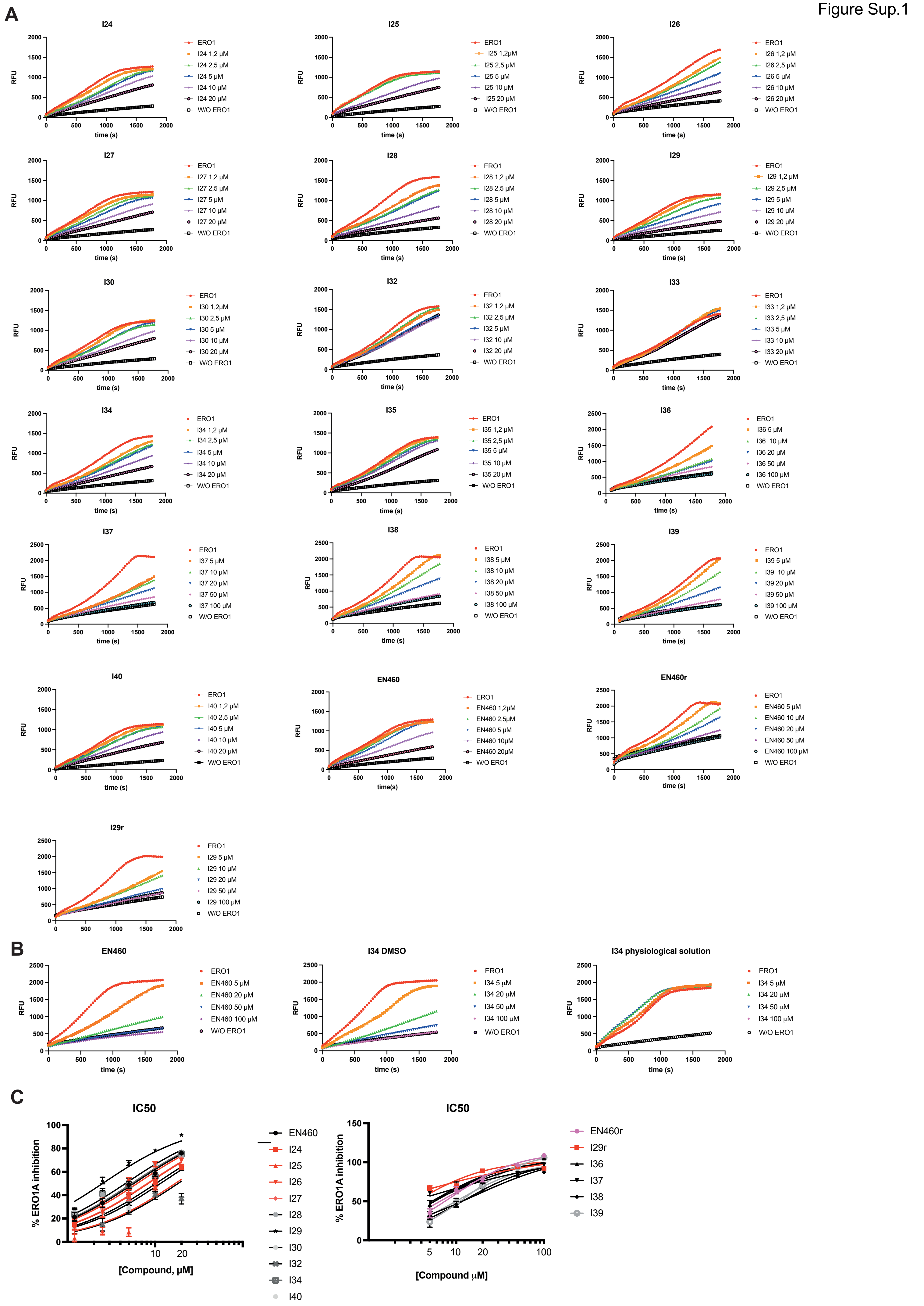
A
I24
ERO1
I24 1,2 µM
I24 2,5 µM
I24 5 µM
I24 10 µM
I24 20 µM
W/O ERO1
I25
I25 1,2µM
I25 2,5 µM
I25 5 µM
I25 10 µM
I25 20 µM
I26
I26 1,2 µM
I26 2,5 µM
I26 5 µM
I26 10 µM
I26 20 µM
I27
I27 1,2 µM
I27 2,5 µM
I27 5 µM
I27 10 µM
I27 20 µM
I28
I28 1,2 µM
I28 2,5 µM
I28 5 µM
I28 10 µM
I28 20 µM
I29
I29 1,2 µM
I29 2,5 µM
I29 5 µM
I29 10 µM
I29 20 µM
I30
I30 1,2µM
I30 2,5 µM
I30 5 µM
I30 10 µM
I30 20 µM
I32
I32 1,2 µM
I32 2,5 µM
I32 5 µM
I32 10 µM
I32 20 µM
I33
I33 1,2 µM
I33 2,5 µM
I33 5 µM
I33 10 µM
I33 20 µM
I34
I34 1,2 µM
I34 2,5 µM
I34 5 µM
I34 10 µM
I34 20 µM
I35
I35 1,2 µM
I35 2,5 µM
I35 5 µM
I35 10 µM
I35 20 µM
I36
I36 5 µM
I36 10 µM
I36 20 µM
I36 50 µM
I36 100 µM
I37
I37 5 µM
I37 10 µM
I37 20 µM
I37 50 µM
I37 100 µM
I38
I38 5 µM
I38 10 µM
I38 20 µM
I38 50 µM
I38 100 µM
I39
I39 5 µM
I39 10 µM
I39 20 µM
I39 50 µM
I39 100 µM
I40
I40 1,2 µM
I40 2,5 µM
I40 5 µM
I40 10 µM
I40 20 µM
EN460
EN460 1,2µM
EN460 2,5µM
EN460 5 µM
EN460 10µM
EN460 20µM
EN460r
EN460 5 µM
EN460 10 µM
EN460 20 µM
EN460 50 µM
EN460 100 µM
I29r
I29 5 µM
I29 10 µM
I29 20 µM
I29 50 µM
I29 100 µM
RFU
time (s)
time(s)
B
EN460
EN460 5 µM
EN460 20 µM
EN460 50 µM
EN460 100 µM
I34 DMSO
I34 5 µM
I34 20 µM
I34 50 µM
I34 100 µM
I34 physiological solution
C
IC50
% ERO1A inhibition
[Compound, µM]
EN460
I24
I25
I26
I27
I28
I29
I30
I32
I34
I40
[Compound µM]
EN460r
I29r
I36
I37
I38
I39

A

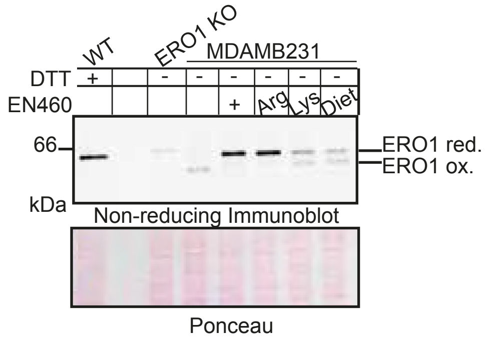
WT
ERO1 KO
MDAMB231
DTT
EN460
+
Arg
Lys
Diet
66
kDa
ERO1 red.
ERO1 ox.
Non-reducing Immunoblot
Ponceau

B

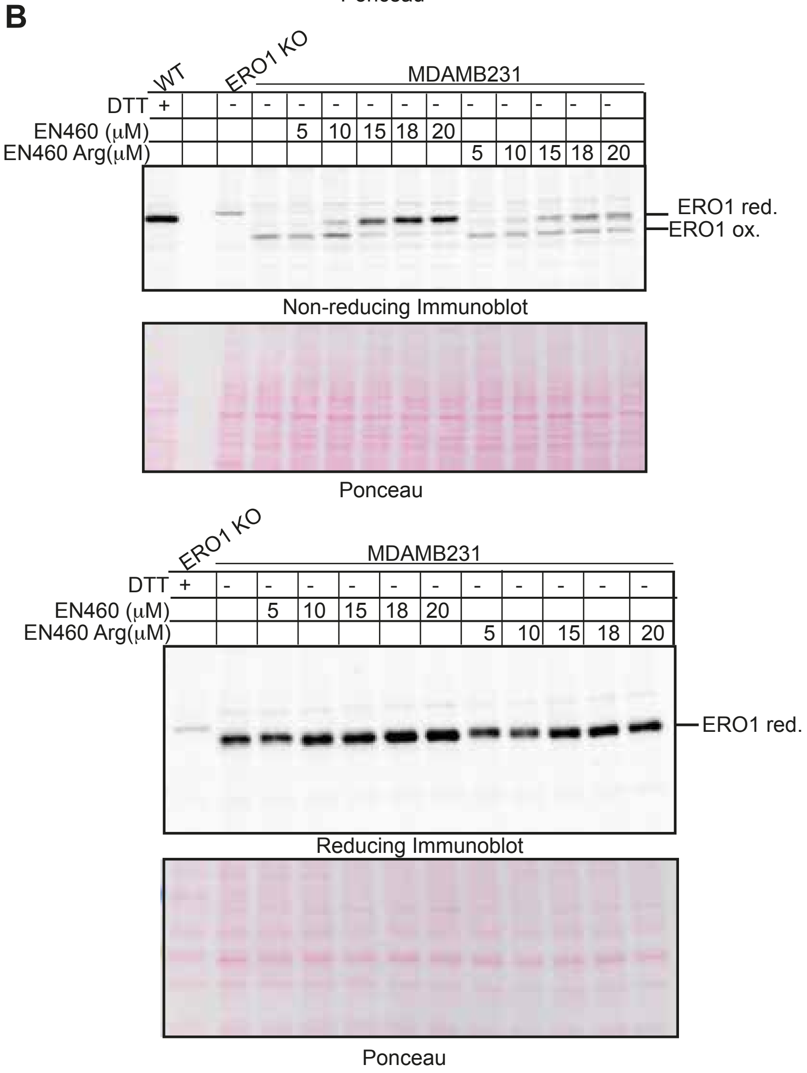
WT
ERO1 KO
MDAMB231
DTT
EN460 (μM)
EN460 Arg(μM)
5
10
15
18
20
ERO1 red.
ERO1 ox.
Non-reducing Immunoblot
Ponceau
ERO1 KO
MDAMB231
DTT
EN460 (μM)
EN460 Arg(μM)
ERO1 red.
Reducing Immunoblot
Ponceau

Figure Supplementary 3

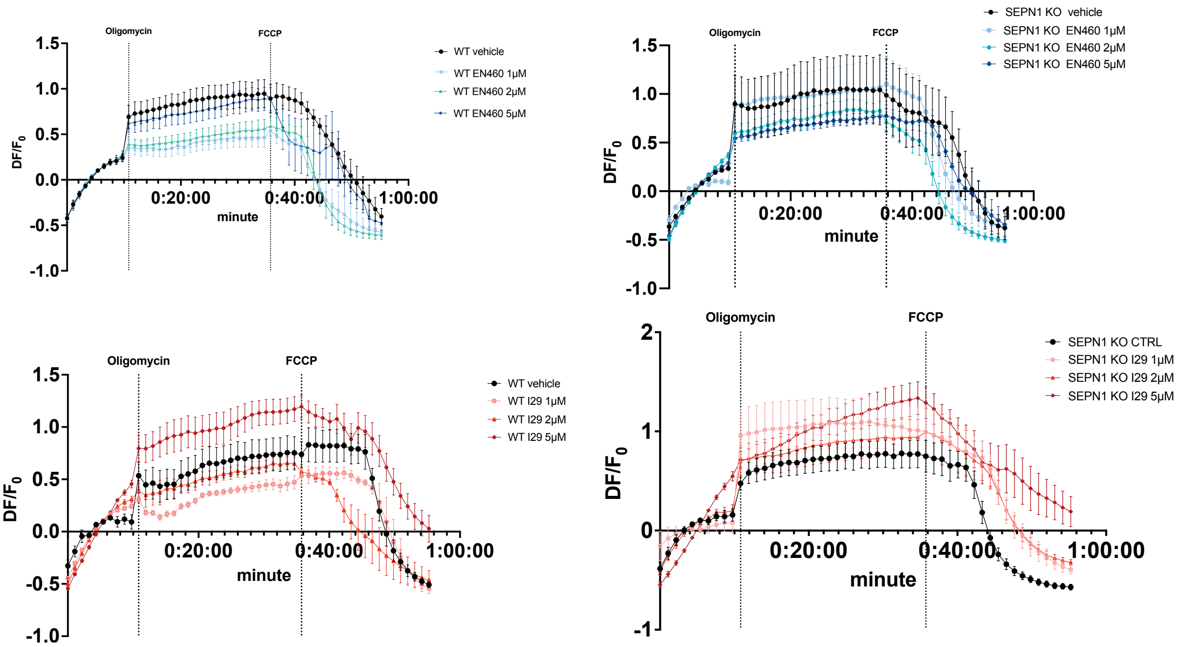

Supplement: Supplementary material [file NIHMS2170937-supplement-Supplementary_material.pdf]
